# Colistin resistance in Gram-negative bacteria analysed by five phenotypic assays and inference of the underlying genomic mechanisms

**DOI:** 10.1186/s12866-021-02388-8

**Published:** 2021-11-20

**Authors:** Diana Albertos Torres, Helena M. B. Seth-Smith, Nicole Joosse, Claudia Lang, Olivier Dubuis, Magdalena Nüesch-Inderbinen, Vladimira Hinic, Adrian Egli

**Affiliations:** 1 Applied Microbiology Research, Department of Biomedicine, University of Basel, Basel, Switzerland; 2 Clinical Bacteriology and Mycology, University Hospital Basel, Basel, Switzerland; 3 Unilabs Bern-Mittelland, Bern, Switzerland; 4 Clinical Microbiology, Viollier AG, Allschwil, Switzerland; 5 Institute for Food Safety and Hygiene, University of Zurich, Zurich, Switzerland

**Keywords:** Colistin, Resistance, Antimicrobial susceptibility testing, WGS, Antimicrobial resistance genes

## Abstract

**Background:**

Colistin is used against multi-drug resistant pathogens, yet resistance emerges through dissemination of plasmid-mediated genes (*mcr*) or chromosomal mutation of genes involved in lipopolysaccharide synthesis (i.e. *mgrB, phoPQ, pmrCAB).* Phenotypic susceptibility testing is challenging due to poor diffusion of colistin in agar media, leading to an underestimation of resistance. Performance of five phenotypic approaches was compared in the context of different molecular mechanisms of resistance. We evaluated Vitek 2® (bioMérieux, AST N242), Colistin MIC Test Strip (Liofilchem Diagnostici), UMIC (Biocentric), and Rapid Polymyxin™ NP test (ELITechGroup) against the standard broth microdilution (BMD) method. We used whole genome sequencing (WGS) to infer molecular resistance mechanisms. We analysed 97 *Enterobacterales* and non-fermenting bacterial isolates, largely clinical isolates collected up to 2018. Data was analysed by comparing susceptibility categories (susceptible or resistant) and minimal inhibitory concentrations (MIC). Susceptibility category concordance is the percentage of test results sharing the same category to BMD. MIC concordance was calculated similarly but considering ±1 MIC titre error range. We determined genomic diversity by core genome multi locus sequencing typing (cgMLST) and identified putative antimicrobial resistance genes using NCBI and CARD databases, and manual annotation.

**Results:**

Of 97 isolates, 54 (56%) were resistant with standard BMD. Highest susceptibility category concordance was achieved by Rapid Polymyxin™ NP (98.8%) followed by UMIC (97.9%), Colistin E-test MIC strip (96.9%) and Vitek 2® (95.6%). Highest MIC concordance was achieved by UMIC (80.4%), followed by Vitek 2® (72.5%) and Colistin E-test MIC strip (62.9%). Among resistant isolates, 23/54 (43%) were intrinsically resistant to colistin, whereas 31/54 (57%) isolates had acquired colistin resistance. Of these, *mcr-1* was detected in four isolates and *mcr-2* in one isolate. Non-synonymous mutations in *mgrB, phoQ, pmrA, pmrB*, and *pmrC* genes were encountered in *Klebsiella pneumoniae, Escherichia coli,* and *Acinetobacter bereziniae* resistant isolates. Mutations found in *mgrB* and *pmrB* were only identified in isolates exhibiting MICs of ≥16 mg/L.

**Conclusions:**

The Rapid Polymyxin™ NP test showed highest categorical concordance and the UMIC test provided MIC values with high concordance to BMD. We found colistin resistance in diverse species occurred predominantly through spontaneous chromosomal mutation rather than plasmid-mediated resistance.

**Supplementary Information:**

The online version contains supplementary material available at 10.1186/s12866-021-02388-8.

## Background

Colistin is an antimicrobial agent of the polymyxin class. Although still widely used in veterinary medicine, colistin usage in human medicine was initially restricted to topical administrations due to its nephrotoxic and neurotoxic properties if given systemically [1]. However, due to the recent dissemination of multidrug resistant (MDR) bacteria around the world, colistin has been increasingly used as a last resort antimicrobial for treatment of difficult-to-treat infections caused by MDR Gram-negative pathogens [1–3].

Colistin is a cationic polypeptide containing an acylated tripeptide chain at its N-terminus responsible for the toxicity of colistin. The mechanism of action relies on the interaction of the hydrophobic region of the fatty acid and phosphate groups of the lipid A of the lipopolysaccharide (LPS). This interaction displaces the divalent cations that naturally stabilize the outer bacterial membranes leading to leakage of cellular compounds and, ultimately, cell death [4–6]. Although this is the main mode of action, other mechanisms have been described such as inhibition of respiratory enzymes NDH-2 [5, 7, 8] and neutralization of the LPS, which may help prevent septic shock [9]. Due to its mechanism of action, colistin is highly effective against most *Enterobacterales* species and non-fermenting Gram-negative bacteria such as *Acinetobacter baumannii* and *Pseudomonas aeruginosa*. Conversely, colistin is not active against Gram-positive bacteria, Gram-negative cocci, and anaerobic bacteria. Some *Enterobacterales* species are intrinsically resistant to colistin, such as *Serratia marcescens*, *Morganella morganii*, *Proteus mirabilis*, and *Burkholderia* spp. due to the constitutive expression of genes (i.e. *eptB*) that lead to the modification of the LPS and an increase in its charge [4, 5, 10–14]. *Hafnia* spp. have also been suggested to be intrinsically resistant [14, 15].

Bacteria have developed resistance mechanisms against colistin mainly through the modification of the LPS. This is achieved by the addition of 4-amino-4-deoxy-L-arabinose (L-Ara-4 N) or phosphoethanolamine (pEtN) to lipid A that increases the positive charge of LPS and thus reduces its affinity to colistin [5, 6, 16]. The synthesis and addition of L-Ara-4 N and pEtN is mediated by the *PmrAB* and *PhoPQ* two-component system genes and its regulators genes (i.e. *mgrB*) but also through plasmid-mediated genes like mobile colistin resistance (*mcr*) [5, 16–18]. Colistin resistance by alteration of LPS has been widely described in several species like *K. pneumoniae* and *E. coli* [4, 5, 10, 19–22]. Other species, like *A. baumannii* acquire resistance due to the complete loss of LPS by inactivation of the lipid A biosynthesis genes (*lpxA, lpxC* and *lpxD*) [5, 23], or by alteration the expression of genes related to LPS synthesis or genes related to electrostatic modifications of the cell surface (*pmrAB*, *adeRS*) [24, 25].

In view of concerns around the emergence of resistance, it is of critical importance that reliable tools for susceptibility testing are available. However, susceptibility testing is a challenge due to the cationic nature of colistin, which causes it to adhere to the negatively charged polystyrene surfaces used in routine laboratory plates [26], but also due to its poor diffusion in agar because of its large molecular size. In 2016 EUCAST warned about the difficulties of colistin testing using disk diffusion and gradient test, as these methods seem to underestimate resistance. EUCAST recommends broth microdilution (BMD) as the only valid method, and that it should be performed with sulphate salts of polymyxins in cation-adjusted Mueller-Hinton broth, without any additives like polysorbate-80 (P-80) in trays made of polystyrene [27]. Nevertheless, this method may be difficult to implement in a routine diagnostic laboratory since other assays, such as disk diffusion or automated testing like Vitek 2® or BD Phoenix™, are commonly used for colistin susceptibility testing and are often part of laboratory automation.

The goal of our study was to first compare the performance of four different diagnostic assays for colistin susceptibility testing against BMD: Vitek 2® (bioMérieux, AST N242), Colistin E-Test MIC Strip (Liofilchem Diagnostici), UMIC (Biocentric), and Rapid Polymyxin™ NP test (ELITechGroup). We aimed to find the most accurate, robust and easy-to-perform assay suitable for the daily usage in the routine microbiology laboratory. Our second aim was to determine the underlying genetic mechanisms of resistance using whole genome sequencing and compare this against the minimal inhibitory concentrations (MICs).

## Results

### Phenotypic colistin resistance in the strain collection

**Fig. 1:**
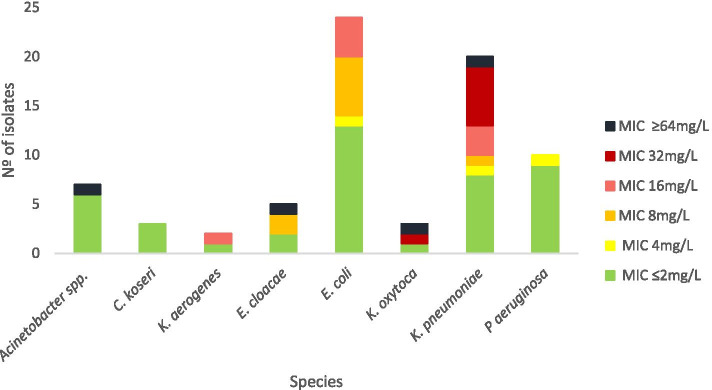
Colistin resistance in non-intrinsically resistant bacteria. MICs were determined by broth microdilution methods (BMD) and interpreted according to EUCAST breakpoints (Version 10.0, 2020)

Of the 97 isolates tested, 54 (56%) were resistant to colistin by the standard BMD testing and 43 (44%) were susceptible. Among the resistant isolates, 23 (43%) belonged to the bacterial species *P. vulgaris, P. mirabilis, S. marcescens* and *H. alvei* and thus possess intrinsic resistance. Whereas 31 (57%) belonged to *Acinetobacter spp., E. cloacae, E. coli, K. aerogenes, K. pneumoniae, K. oxytoca,* and *P. aeruginosa* which display various mechanisms of acquired resistance. Among these latter non-intrinsically resistant isolates, three (10%) had a MIC value of 4 mg/L, nine (29%) had a MIC of 8 mg/L, eight (26%) had a MIC of 16 mg/L, seven (23%) had a MIC value of 32 mg/L, and four (13%) isolates displayed a MIC of ≥64 mg/L (Fig. 1). A total of 19 (20%) isolates had a MIC value ≥16 mg/L, belonging to *Acinetobacter spp*. (*n* = 1), *E. cloacae* (*n* = 1), *E. coli* (*n* = 4), *K. aerogenes* (*n* = 1), *K. oxytoca* (*n* = 2), and *K. pneumoniae* (*n* = 10). Due to the high number of isolates exhibiting a MIC between 4 and 8 mg/L compared to the few isolates with higher levels of resistance, we considered ≥16 mg/L to be higher MIC values in this study. Additionally, these isolates exhibited specific genomic traits explained in more detail below.

### Rapid Polymyxin™ NP test shows highest concordance with susceptibility category and UMIC with MIC measurements

Susceptibility category concordance (susceptible or resistant) between the BMD gold standard and the test protocols (Vitek 2®, Colistin MIC Test Strip, UMIC, and Rapid Polymyxin™ NP test) is shown in Table 1. The highest overall susceptibility category concordance was achieved with Rapid Polymyxin™ NP test (98.8%), followed by UMIC (97.9%), Colistin E-test MIC strip (96.9%), and Vitek 2® (95.6%). We calculated the sensitivity and specificity for the susceptibility category according to the BMD reference standard in all isolates. The highest sensitivity was shown for the Rapid Polymyxin™ NP test (98.8%) with a 100% specificity.

**Table 1:** Susceptibility category (susceptible or resistant) concordance of different assays compared to reference BMD method

Method	BMD	Vitek 2®^**a**^		Rapid PolymyxinTM NP^**b**^		UMIC		Colistin E-test MIC strip	
Species	S/R	S/R	% concordance	S/R	% concordance	S/R	% concordance	S/R	% concordance
***Acinetobacter spp.***	6/1	4/0	100	1/1	50	6/1	100	6/1	100
***C. koseri***	3/0	3/0	100	3/0	100	3/0	100	3/0	100
***K. aerogenes***	1/1	1/1	100	1/1	100	1/1	100	1/1	100
***E. cloacae***	2/3	3/2	80	2/3	100	2/3	100	3/2	80
***E. coli***	13/11	13/11	100	13/11	100	13/11	100	13/11	100
***Hafnia spp.***	0/15	1/14	93.3	0/15	100	0/15	100	0/15	100
***K. oxytoca***	1/2	1/2	100	1/2	100	1/2	100	1/2	100
***K. pneumoniae***	8/12	9/10	94.7	9/11	95	9/11	95	9/11	95
***M. morganii***	0/3	0/3	100	0/3	100	0/3	100	0/3	100
***P. mirabilis***	0/2	0/2	100	0/2	100	0/2	100	0/2	100
***P. vulgaris***	0/1	0/1	100	0/1	100	0/1	100	0/1	100
***P. aeruginosa***	9/1	8/0	87.5	NA	NA	10/0	90	10/0	90
***S. marcescens***	0/2	0/2	100	0/2	100	0/2	100	0/2	100
**Total**	**43/54**	**43/48**	**95.6**	**30/52**	**98.8**	**45/52**	**97.9**	**46/51**	**96.9**

*BMD* Broth microdilution, *S* Susceptible, *R* Resistant, *NA* Not applicable

^a^A total of 91 isolates were tested by Vitek 2® method. Isolates not tested by Vitek 2® test was due to low growth, but were excluded from the comparison analysis with BMD

^b^A total of 82 were tested by the Rapid PolymyxinTM NP method. Isolates not tested by Rapid PolymyxinTM NP test were excluded from the comparison analysis with gold standard

Exploring subsets of bacterial isolates, the test that performed better compared to the gold standard in *Enterobacterales* was the UMIC test, with a concordance of 98.7%. The Rapid Polymyxin™ NP test also achieved a high concordance level (98.5%), whereas the Vitek 2® and the Colistin E-test MIC strip were concordant only in 96.2% of the tested isolates in both tests. In susceptibility testing for non-fermenting bacteria, the highest concordance to BMD was the UMIC (94.1%) and Colistin E-test MIC strip (94.4%) followed by Vitek 2® (90.9%). The Rapid Polymyxin™ NP test is specifically designed to detect polymyxin resistance among *Enterobacterales* [28]. The susceptibility concordance with BMD was high for the subset of *Enterobacterales* isolates included in this study (98.5%). However, and as expected, the performance for non-fermenting bacteria was poor, reaching a concordance percentage of only 50% in the *Acinetobacter* spp. No differences were observed in the capability to detect resistant isolates in non-fermenting and fermenting *Enterobacterales* between the different assays used in this study (Additional file 1).

MIC concordance of the different assays compared to BMD is shown in Table 2. Concordance was established as the same MIC value or ± 1 titre difference as that of the gold standard. All intrinsic resistant species were excluded from the analysis since no MIC value was obtained from the reference standard method as they were automatically considered as resistant isolates. The highest concordance was achieved with UMIC test (80.4%), followed by Vitek 2® (72.5%). The Colistin E-test MIC strip had the lowest concordance (62.9%) to BMD.

**Table 2:** MIC concordance of different assays compared to the reference BMD method

Method	BMD	Vitek 2®		UMIC		Colistin E-test MIC strip	
Specie	No. Isolates tested	No. Isolates tested	No. of concordant isolates [%]^**a**^	No. Isolates tested	No. of concordant isolates [%]^**a**^	No. Isolates tested	No. of concordant isolates [%]^**a**^
***Acinetobacter spp.***	7	4	4 [100.0]	7	7 [100.0]	7	6 [85.7]
***C. koseri***	3	3	3 [100.0]	3	2 [66.7]	3	2 [66.7]
***K. aerogenes***	2	2	1 [50.0]	2	1 [50.0]	2	2 [100.0]
***E. cloacae***	5	5	3 [60.0]	5	5 [100.0]	5	3 [60.0]
***E. coli***	24	24	17 [70.8]	24	19 [79.2]	24	13 [54.2]
***Hafnia spp.***	15	15	14 [93.3]	15	14 [93.3]	15	13 [86.7]
***K. oxytoca***	3	3	1 [33.3]	3	3 [100.0]	3	1 [33.3]
***K. pneumoniae***	20	19	16 [84.2]	20	17 [85.0]	20	13 [65.0]
***P. aeruginosa***	10	8	7 [87.5]	10	10 [100.0]	10	8 [80.0]
**Total**	**97**	**91**	**66 [72.5]**	**97**	**78 [80.4]**	**97**	**61 [62.9]**

*BMD* Broth microdilution method

^a^Concordance was considered as the same MIC value or as one titre difference to that of the reference value obtained by BMD

**Fig. 2:**
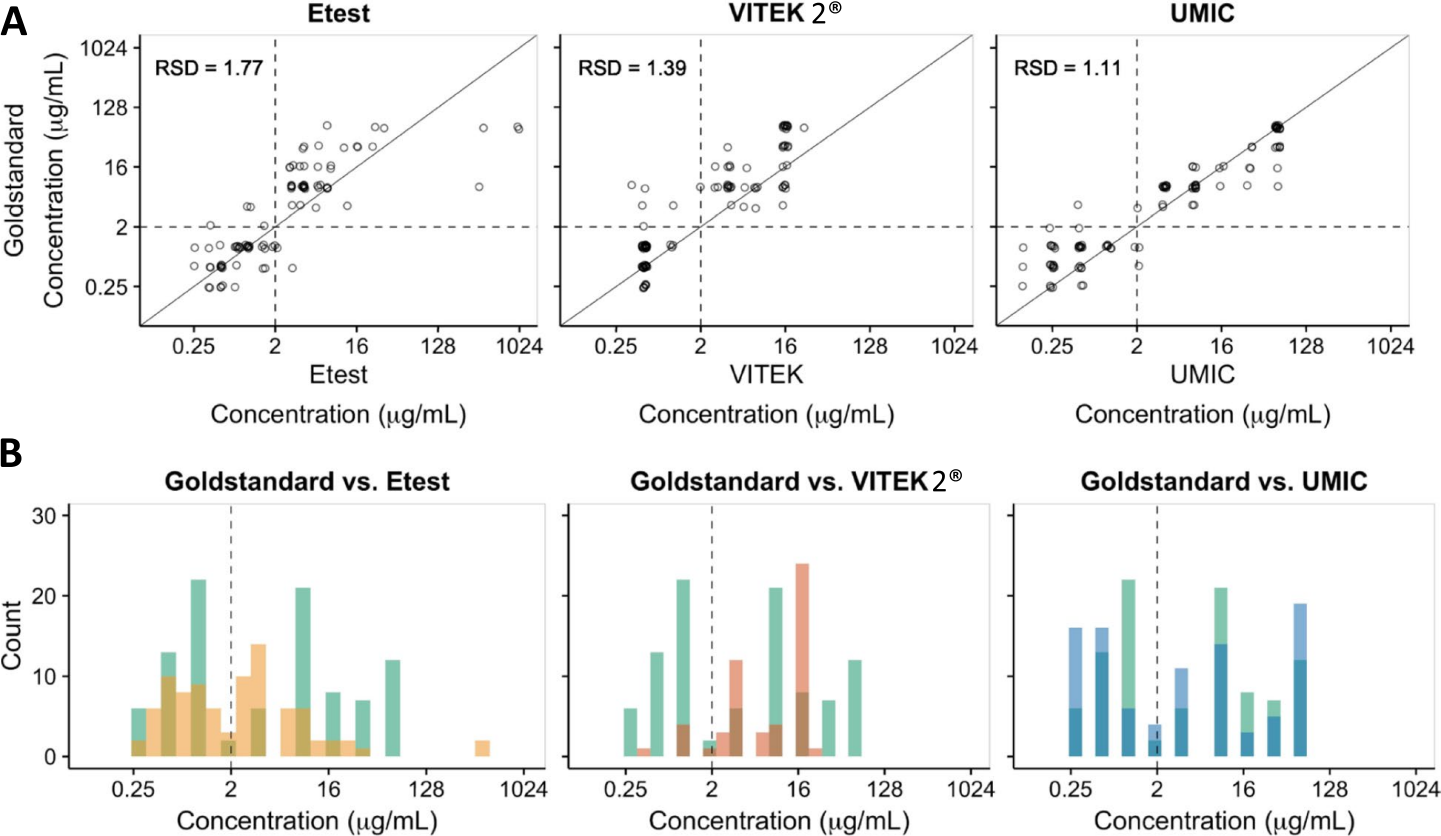
Distribution of MICs in BMD vs. respective phenotypic test. **A** MIC correlation of Colistin E-test MIC strip, Vitek 2®, and UMIC against the reference BMD. RSD, relative standard deviation. **B** Number of isolates per MIC tested by Colistin E-test MIC strip (yellow bars), Vitek 2® (orange bars), and UMIC (blue bars) compared to the number of isolates per MIC tested by the reference BMD. Dark coloured bars indicate concordant results

Additionally, for *Enterobacterales* species the most concordant test to the gold standard was UMIC (76.25%), followed by Vitek 2® (69.62%) and Colistin E-test MIC strip (58.75%). Similarly, the highest concordance for non-fermenting bacteria was found in the UMIC test (100%). The Vitek 2® test and the Colistin E-test MIC strip achieved a concordance to the gold standard of 91.67 and 82.35%, respectively. Figure 2 shows the MIC distribution of the BMD vs. each method and the MIC distribution for all isolates.

### Heterogenous molecular causes of colistin resistance

**Fig. 3:**
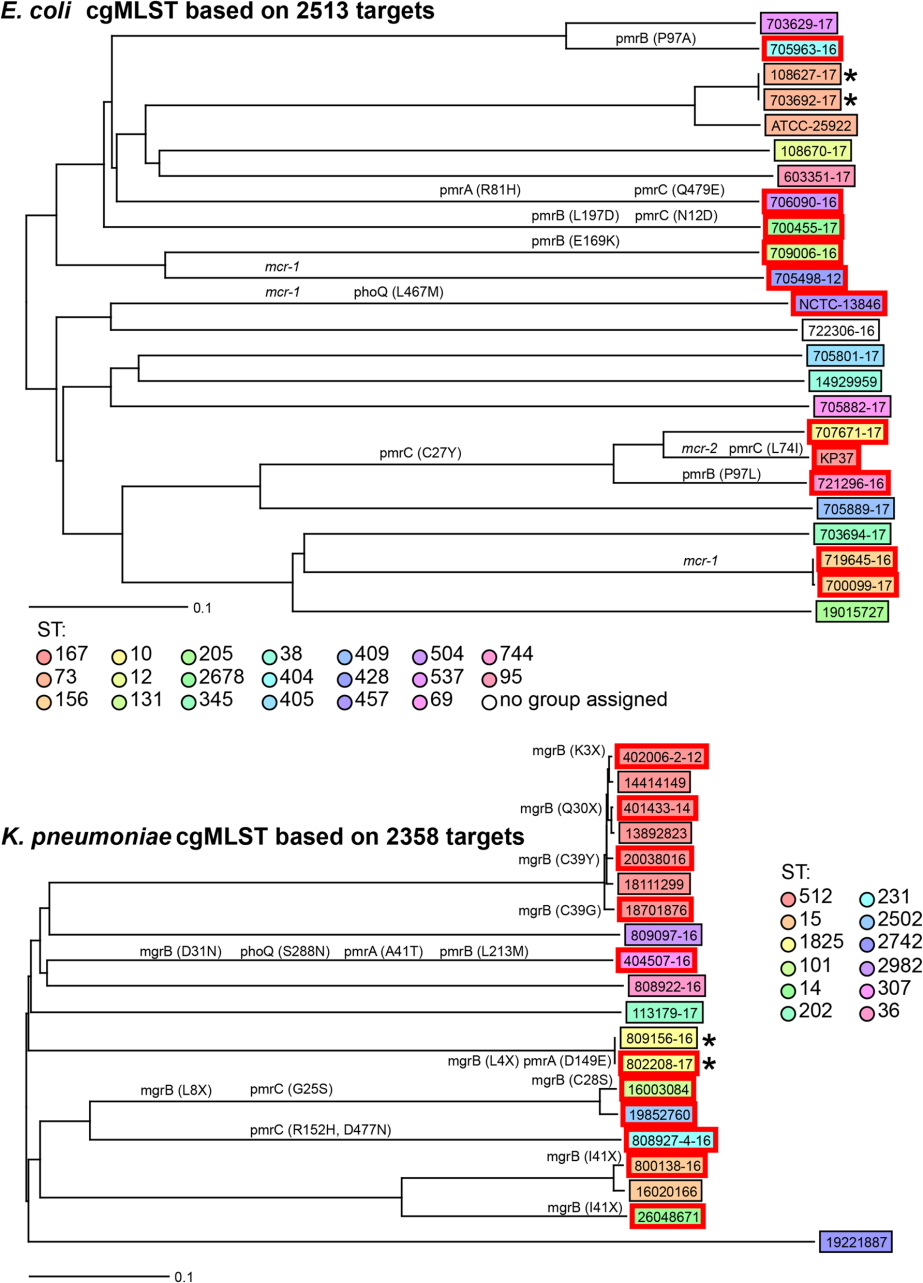
Core genome MLST Neighbour Joining trees of *E. coli* and *K. pneumoniae*. MLST sequence types are shown. Scale bar indicates the number of variant alleles relative to the total number of targets for that species. * indicates isolates from the same patient. Red boxes around isolate names indicate colistin resistance. Mutations associated with colistin resistance are shown on the relevant branches. Acquired plasmid-mediated genes associated with resistance are also shown on the relevant branches in *italics*

MLST sequence type (ST) designation and core genome MLST (cgMLST) analyses were used to explore the genetic diversity between bacterial isolates. The STs of isolates for which species MLST schemes exist was determined (Additional file 2). cgMLST comparison could only be performed on species with more than two isolates. The diversity within *E. coli* and *K. pneumoniae* are shown in Fig. 3. Isolates were genomically diverse in the cgMLST, with the exception of isolates from the same patients (indicated with * in the figures): in some cases multiple isolates belonged to the same ST, such as *K. pneumoniae* ST512 (*n* = 7), *K. pneumoniae* ST1825 (*n* = 2), *E. coli* ST73 (*n* = 3), and *E. coli* ST156 (*n* = 2).

Genes encoding colistin resistance were identified first by comparing genome assemblies against known databases. This identified *mcr-1* in four isolates (NCTC-13846 as the control, 700,099-17, 719,645-16 and 705,498-12) and *mcr-2* in one isolate (KP-37 as expected), but these results did not explain all the phenotypic resistance.

Individual genomic analysis to determine the underlying colistin resistance mechanisms was performed, looking at genes previously described as being involved in colistin resistance. These genes were extracted from the genome and compared between sensitive and resistant isolates. This could only be performed on isolates within species with sufficient numbers of each, namely *K. pneumoniae* (*n* = 20) and *E. coli* (*n* = 24). The nucleotide sequences and derived protein sequences from *phoPQ* and *pmrCAB* in both species, and additionally *mgrB* in *K. pneumoniae* isolates were compared between resistant and susceptible isolates (Additional files 3 and 4). Variations unique to the resistant isolates are described in Table 3.

**Table 3:** Mutations in associated colistin-resistance proteins in *E. coli*, *K. pneumoniae* and *A. bereziniae* resistant isolates

Species	Isolate	MIC (mg/L)^**a**^	Amino acid change						Plasmid mediated resistance
			MgrB	PmrB	PmrA	PmrC	PhoP	PhoQ	***mcr***
***K. pneumoniae***	404,507-16	≥64	D31N	L213M	A41T			S288N	
	4,002,006-2	32	K3*						
	16,003,084	32	C28S		A217V	G25S			
	20,038,016	32	C39Y						
	808,927-16	32	L8*			R152H			
						D477N			
	26,048,671	32	I41* (ISKpn26)^b^						
	800,138-16	32	I41* (ISEcp1)^b^						
	401,433-14	16	Q30*						
	802,208-17	16	L4*		D149E				
	187,701,876	16	C39G						
	19,852,760	8			A217V	G25S			
	809,156-16	4			D149E				
***E. coli***	721,296-16	16		P97L		C27Y			
	700,455-17	16		L197D		N12D			
	709,006-16	16		E169K					
	705,963-16	16		P97A					
	700,099-17	8							*mcr 1.1*
	706,090-16	8			R81H	Q479E			
	NCTC-13846	8						L467M	*mcr 1.1*
	KP-37-MCR-2-18	8				C27Y			*mcr 2*
						L74I			
	707,671-17	8				C27Y			
	719,645-16	8							*mcr 1.1*
	705,498-12	4							*mcr 1.1*
***A. bereziniae***	502,814-14	≥64		Q242R					

^a^MIC values obtained by the reference broth microdilution method

^b^Nucleotide sequence interrupted by insertion sequence. * indicates premature stop codons or termination in the amino acid sequence indicates premature stop codons or termination in the amino acid sequence

None of the analysed *K. pneumoniae* isolates were carriers of *mcr* genes. A key finding within *K. pneumoniae* isolates was the presence of mutations in *mgrB* causing amino acid substitutions, premature stop codons, or termination resulting from insertion sequences *ISEcp1* (IS138 family) and *ISkpn26* (IS5 family). This applied to all resistant isolates with MIC values of ≥16 mg/L, whereas sensitive isolates have intact versions of *mgrB*. Further mutations leading to amino acid substitutions were found in other genes: the isolate with the highest MIC (≥64 mg/L) possesses additional amino acid substitutions in the PhoQ, PmrA, and PmrB proteins compared to those from sensitive isolates. Similarly, two isolates with MIC of 32 mg/L have amino acid substitutions in the PmrA and/or PmrC proteins in addition to MgrB. Two resistant isolates with MICs of 4 mg/L and 8 mg/L have an unaltered *mgrB* gene but possesses non-synonymous mutations in the *pmrA* and/or *pmrC* genes.

Only four of the resistant *E. coli* isolates, with MICs between 4 and 8 mg/L were carriers of the *mcr-1.1* gene. Gene comparisons within *E. coli* showed that those with a MIC of 16 mg/L have amino acid changes in the PmrB protein sequence, whereas the isolates with a lower MIC (4-8 mg/L) possess PmrB identical to those in sensitive isolates. Two isolates with a MIC of 8 mg/L that did not harbour *mcr* genes had an unaltered PmrB protein sequence compared to the susceptible isolates but had amino acid substitutions in the protein sequences of PmrA or PmrC*.*

All the *A. bereziniae* isolates (*n* = 4) were isolated from the same patient, and were subjected to analysis of *pmrAB* and *phoPQ* genes, as well as *lpxA, lpxD, lpxC* genes since mutations in the latter genes produce a total loss of lipid A leading to colistin resistance in *Acinetobacter* spp. [5]. The single resistant isolate (MIC 64 mg/L) had a mutation causing amino acid change Q242R in PmrB (Additional file 5). No mutations were found in the other genes assessed.

## Discussion

We compared the performance of four commercial assays for colistin susceptibility testing to the BMD gold standard. We used a panel of different bacterial species to reflect common situations found in routine diagnostics.

Numerous comparative studies dealing with colistin susceptibility testing have been published. Several studies find the highest rate of very major errors (VMEs), considered as discrepancy in the susceptibility category between a commercial kit and the reference method, with Colistin E-test MIC strip colistin [6, 29, 30], which was also confirmed with our study. Vitek 2® has been reported as reliable in some studies, but not in others [6, 31, 32]. In our study, the Rapid Polymyxin™ NP test showed the highest concordance with the gold standard in the susceptibility category agreement. However, in our experience, the main drawback is lack of MIC values and difficulties in interpretation of the colorimetric test. Additionally, the Rapid Polymyxin™ NP test showed a low concordance (50%) to the reference method when assessing the susceptibility of *P.aeruginosa* and *Acinetobacter* spp*.,* but a high concordance for *Enterobacterales*. The Rapid Polymyxin™ NP test used in this study was clinically validated only with the most representative species of *Enterobacterales* [33]. Here we show that the concordance was low for the *P.aeruginosa* (*n* = 10) and *Acinetobacter* spp*.* (*n* = 7) isolates included in this study and, therefore, it would be interesting to analyse the performance of this test with a larger subset of isolates. The UMIC test was easy to perform and showed highest categorical and MIC agreement. This has, however, not been confirmed in other studies. Due to the high performance of the UMIC assay, this assay was established into routine diagnostics at the University Hospital Basel.

For this study we included 54 (56%) colistin resistant isolates, of which 23 (43%) were intrinsically resistant whereas 31 (57%) had an acquired resistance mechanism. All the isolates were collected up to 2018 but the susceptibility or resistance to colistin was determined following the EUCAST Version 10.02020 [23]. The breakpoints for *Enterobacterales* in the 2020 EUCAST version remained unaltered compared to previous versions from before 2018 [34] that applied when the isolates were collected, whereas the breakpoint for *Pseudomonas spp.* changed to lower MIC values (S ≤ 2 mg/L, R > 2 mg/L). However, these cut off changes did not affect the percentage of resistant isolates included in this study. Four *E. coli* isolates were carriers of the *mcr-1* gene, and one isolate was positive for *mcr-2.* As expected, none of the *mcr*-positive isolates displayed a high MIC value, considered in this study as ≥16 mg/L [35, 36].

Several chromosomal mutations have been reported to be linked to colistin resistance in various species [4, 5, 16, 37, 38]. Most of the reported mutations have been found in genes involved in signalling pathways that lead to modification or loss of lipid A from the LPS. Mutations in genes related to efflux pumps have also been described [39, 40]. The most commonly reported colistin-related mutations are encountered in the *pmrA/pmrB* and *phoP/phoQ* genes encoding two-component systems in several Gram-negative bacteria such as *E. coli, K. pneumoniae, P. aeruginosa* and *A. baumannii* [5]. The *mgrB* gene encodes a negative regulator of the PhoPQ system. *mgrB* inactivation or disruption have been associated with colistin resistance in *K. pneumoniae* [19, 41]. Although these are the most commonly described genes the chromosomal mechanisms leading to resistance are highly diverse and involve numerous and different mutations and genes [5].

In this study, we found that all the *K. pneumoniae* isolates displaying a high MIC (≥16 mg/L) had a disrupted or altered *mgrB* gene and an altered protein sequence, whereas isolates with MICs ≤8 mg/L displayed a wild type *mgrB*. Some of the amino acid alterations encountered in MgrB during this study (truncations at K3 and Q30, substitutions at C28S, C39Y/G and disruption of the gene by *ISKpn26* and *ISEcp1*) have been described in other studies [41, 42]. However, to our best knowledge, the truncations at L4 and L8, and the D31N amino acid change are novel to this study. Noteworthy, the cgMLST analysis identified several closely related *K. pneumoniae* isolates (ST512) with different susceptibilities to colistin. Within this ST type, all resistant isolates carried mutations in the *mgrB* gene, leading to altered MgrB protein sequences, and in no other colistin resistance-related protein sequences we analysed. However, these alterations were unique in each isolate and appear to have occurred independently. Additionally, we identified further mutations in the *mgrB* gene in isolates from other STs, which also led to high MIC values. These results suggest that mutations of *mgrB* in any of these locations can lead to high colistin resistance in *K. pneumoniae* and that there is not a unique *mgrB* mutation associated with a specific ST type. The single *K. pneumoniae* isolate with a MIC ≥64 mg/L also carried altered *pmrB* and *phoQ* in addition to an *mgrB* mutant (causing D31N). The mutation in *pmrB* (causing L213M) has already been associated with colistin resistance in *K. pneumoniae* [41]. This may indicate that association between *pmrB* and/or *phoQ* mutations with *mgrB* mutations confer a higher resistance than these mutations on their own. Together this data suggests that mutation or inactivation of the *mgrB* gene leads to MICs ≥16 mg/L, with further mutations in pathway genes able to synergistically increase MICs further.

Similarly, all the resistant *E. coli* isolates with MIC of 16 mg/L carried amino acid changes in PmrB. Of these amino acid substitutions (P97L, P97A, E169K and L197D), only that at position 97 has been reported previously, in a clinical colistin resistant isolate from a Lebanese hospital, also displaying a MIC of 16 mg/L [43], although the amino acid change in this position was different to the ones in our study. As far as we are aware, the other two PmrB alterations have not been described, although other alterations within the same domains, namely the HAMP domain (covering residues 92-144) and histidine-kinase domain (residues 145-205) have been reported [5, 10, 37]. Further amino acid changes were found in PmrA, PmrC and PhoQ in isolates with MIC between 4 and 8 mg/L, suggesting that alterations in these proteins may confer a lower level of resistance. Of these, only the amino acid change at position 81 in PmrA from *E. coli* has been previously characterized, in an isolate from swine origin and with a colistin MIC of 4 mg/L [22]. In this published case, it was not determined whether the mutation in PmrA was the sole cause of colistin resistance, as other mutations were also identified within this isolate.

Total loss of lipid A of the LPS by alteration of *lxpA, lxpC* and *lpxD* genes has been described as a colistin resistance mechanism in *Acinetobacter baumannii* [5]. Mutations in *pmrAB* also lead to colistin resistance in this species [23, 44, 45]. A published comparison between susceptible and resistant *A. baumannii* isolates after in vivo exposure in three different patients found different but unique mutations in *pmrB* that led to resistance levels of 16 mg/L [45]. Similarly, we found that the only difference between the susceptible and resistant *A. bereziniae* isolates from the same patient was a single amino acid change (Q242R) in the PmrB protein. Interestingly, the resistant isolate was highly resistant to colistin (MIC ≥64 mg/L). Detection of an alteration at this same position has so far not been reported. This may suggest that alterations in PmrB in *Acinetobacter* spp. and/or the specific PmrB alteration encountered in this study (Q242R) are putative mechanisms that confer high levels colistin resistance.

Typing isolates shows that resistance can occur in diverse isolates within the species we analysed. Our data also suggests the impact of selective pressure, with stochastic presence of resistance throughout the phylogenies, and resistant isolates of *A. bereziniae* and *K. pneumoniae* (ST512) closely related to sensitive isolates.

This study has several limitations. Only small number of isolates (*n* = 10) showed MICs close to the breakpoint. Future studies should include more isolates close to the breakpoint. Similarly, a lower number of non-fermenting bacterial isolates were analysed in this study compared to the number of *Enterobacterales* members included in this study. This may have affected the real performance and concordance to BMD. Again, studies including a higher number of non-fermenting bacteria should be carried out to better evaluate the performance of these tests. Secondly, we were not able to investigate putative resistance mechanisms in all species due to the low number of isolates. Thirdly, we have investigated putative resistance mechanisms in diverse clinical isolates and have not confirmed the effects of the observed mutations in isogenic backgrounds.

## Conclusions

In summary, in our clinical setup MIC values provide important information. The UMIC assay provided the highest concordance on MIC values with the reference method. For a categorical assessment the Rapid Polymyxin™ NP test provided highly concordant results. Our genetic study identified highly heterogenous putative causes of resistance. Whereas some resistance assays may cause only small differences in MICs determined, sensitive and precise phenotypic assays are important in routine diagnostics.

## Materials and methods

### Ethical statement

All strains were collected as part of quality control purposes and establishment of new diagnostic assays. All strains were used in anonymized way and no clinical data was collected. For these quality control studies no ethical approval is necessary according to the Human Research Act in Switzerland.

### Clinical isolates and culture conditions

We used 97 isolates from the *Enterobacterales* order and non-fermenting bacteria: 93 from clinical samples in the period from 2008 to 2018 at the clinical microbiology laboratories of the University Hospital Basel, Switzerland; Cantonal Hospital Lucerne, Switzerland and laboratory Viollier in Allschwil, Switzerland (Additional file 6), and four reference strains: *E. coli* ATCC-25922, *E. coli* NCTC-13846 (*mcr-1*), *P. mirabilis* ATCC-25933 and *E. coli* KP-37 (*mcr-2*) [46]. All colistin resistant isolates tested with Vitek 2® (bioMérieux, Marcy’l Etoile, France) from the University Hospital Basel were included in the study. The strain collection included 43 colistin sensitive isolates.

Species identification was performed at the time of diagnosis with matrix-assisted laser desorption ionization time of flight mass spectrometry (MALDI-TOF MS; Bruker, Bremen, Germany) by using the mass-spectrum library and the MALDI Biotyper 3 software (OC 3.1, Bruker Daltonics) at standard conditions. All bacterial isolates were frozen at − 70 °C in cryogenic Microbank™ vials (Pro-Lab Diagnostics, Birkenhead, UK). Prior to testing, the strains were cultured on Columbia agar supplemented with 5% sheep blood (BD Diagnostic Systems, Allschwil, Switzerland) with subsequent subculture after 24 h.

### Assays for colistin susceptibility testing

Standard BMD was performed according to EUCAST recommendations by using 11 concentrations ranging from 0.06 to 64 mg/L including a growth control without colistin [47]. Colistin susceptibility testing with Vitek 2® (bioMérieux) was performed by using the AST N242 card. For calculation of colistin MIC, the following dilutions were tested: 4 mg/L, 16 mg/L and 32 mg/L. MIC determination was performed with Colistin E-test MIC Strip (Liofilchem Diagnostici, Roseto degli Abruzzi, Te, Italy) (Additional file 7). UMIC is a manual broth microdilution test and was performed according to the manufacturer’s instructions. Briefly, a 1:200 dilution of a 0.5 Mc Farland solution of bacteria in Mueller-Hinton II broth was inoculated in the UMIC strips and incubated in a humid atmosphere at 35-37 °C for 18 h. The MICs were read visually (turbid = growth, clear = no growth) (Additional file 7). Rapid Polymyxin™ NP test (ELITechGroup) is based on the colourimetric detection of rapid glucose metabolism associated with bacterial growth, through a pH indicator colour change from orange to yellow, in the presence of a defined concentration of colistin. The test was read after 2 and 3 h of incubation and the results were recorded as either colistin susceptible or colistin resistant (without MIC value) (Additional file 7). Reading of all assays was performed with two independent persons in a blinded fashion. If the results were discrepant, the testing was repeated. Susceptibility (susceptible or resistant) category concordance was considered if there was a categorical agreement to the standard BMD. The MIC variation of ±1 titre range compared to reference MIC was considered as concordant. All MICs were interpreted according to EUCAST Version 10.0, 2020 [48].

### Whole genome sequencing and antimicrobial resistance gene detection

All isolates underwent whole genome sequencing on an Illumina Miseq 2x300bp or NextSeq 2x150bp after NexteraXT library preparation to mean coverage over 35x. All data is available under project number PRJEB47075. Assembly was performed, after trimming with trimmomatic v 0.38 [49], with Unicycler v0.3.0b [50] using standard settings. The assemblies were annotated with Prokka v1.13 [51] and ABRicate v0.8.10 [52] was used to search for antimicrobial resistance genes using the NCBI or CARD AMR gene databases. Species were identified using ribosomal MLST [53] and Average Nucleotide Identity (ANI) comparisons for *Klebsiella* spp. Where discrepancies in species classification between the original MALDI-TOF MS identification and ribosomal MLST identification from whole genome sequencing data occurred, the ribosomal MLST was taken as accurate. In particular this was important for the classification of isolates with known low resolution in MALDI-TOF MS such as *Klebsiella oxytoca* or *Klebsiella michiganensis*, and between species belonging to the *E. cloacae* complex- namely *E. cloacae*, *E. homaechei* and *E. bugandensis.* Assemblies were typed by core genome multi-locus sequencing typing (cgMLST) within Seqphere+ (Ridom, Münster, Germany) using relevant published schemes where available [54] or ad hoc schemes where unavailable for that species. Detailed searches of genome assemblies from resistant isolates and also from susceptible isolates as negative controls, were performed using visualization in Artemis v 18.1.0 [55], with alignments in Jalview v 2.11.1.0 [56]. Where assemblies required checking by remapping, this was done in CLC Genomics Workbench v20.0.2, using default mapping parameters. Insertion sequence families were determined using ISFinder [57].

## Supplementary Information

**Additional file 1.** Susceptibility category (susceptible or resistant) concordance of different assays compared to the reference broth microdilution method in *Enterobacterales* species and non-fermenting bacteria.xlsx showing the percentage of concordance for different test in *Enterobacterales* and non-fermenting bacteria.

**Additional file 2.** Species identification by MALDI-TOF MS and rMLST from whole genome sequencing data.docx showing the difference in species identification by MALDI-TOF MS and rMLST as well as the ST types of every isolate included in this study.

**Additional file 3.*** Klebsiella pneumoniae* protein sequence alignments.pdf showing the protein sequences alignments of all colistin resistance-related proteins analyzed in this study.

**Additional file 4.*** Escherichia coli* protein sequence alignments.pdf showing the protein sequences alignments of all colistin resistance-related proteins analyzed in this study.

**Additional file 5.*** Acinetobacter bereziniae* protein sequence alignments.pdf showing the protein sequences alignments of all colistin resistance-related proteins analyzed in this study.

**Additional file 6.** Species distribution as determined by MALDI-TOF MS. A total of 97 isolates were investigated, including 93 collected collected from the Basel University Hospital (Basel, Switzerland), Cantonal Hospital Luzerne (Luzerne, Switzerland) and Laboratory Viollier (Allschwil, Switzerland)

**Additional file 7.** Different phenotypic assays used for colistin susceptibility testing: UMIC, Colistin E-Test MIC strip and Rapid Polymyxin NP Test.docx showing example images of the phenotypic assays using in this study.

## Data Availability

The dataset(s) supporting the conclusions of this article are included within the article and its additional files, or submitted to the ENA under project PRJEB47075.

## Abbreviations

LPS: Lipopolysaccharide
L-Ara-4 N: 4-amino-4-deoxy-L-arabinose
pEtN: Phosphoethanolamine
MIC: Minimum inhibitory concentration
CPS: Capsular polysaccharide
*pmrA*: Two-component regulator system response regulator PmrA
*pmrB*: Two-component regulator system response regulator PmrB
*phoP*: Response regulator in two-component regulatory system with PhoQ
*phoQ*: Two-component system sensor histidine kinase PhoQ
*mgrB*: PhoP/PhoQ regulator MgrB
*mcr*: Mobilized colistin resistance
*lpxA*: UDP-N-acetylglucosamine acyltransferase
*lpxC*: UDP-3-O-acyl-N-acetylglucosamine deacetylase
lpxD: UDP-3-O-(3-hydroxymyristoyl)glucosamine N-acyltransferase
EUCAST: European Committee on Antimicrobial Susceptibility Testing
BMD: Broth microdilution
MLST: Multilocus sequence typing
cgMLST: Core genome Multilocus sequence typing
NCBI: National Center for Biotechnology Information
CARD AMR: The Comprehensive Antibiotic Resistance Database
ANI: Average Nucleotide Identity
MALDI-TOF MS: Matrix-Assisted Laser Desorption Ionization T-Of-Flight Mass Spectrometry
VME: Very major errors
HAMP: Histidine kinases, adenylyl cyclases, methyl binding proteins, and phosphatases
