# Supplementary material for: Colistin resistance in Gram-negative bacteria analysed by five phenotypic assays and inference of the underlying genomic mechanisms
Source: BMC Microbiol. 2021 Nov 20;21:321. doi: 10.1186/s12866-021-02388-8 (PMC8605564; doi:10.1186/s12866-021-02388-8)
Supplement: Supplementary file 2 — Additional file 2. Species identification by MALDI-TOF MS and rMLST from whole genome sequencing data.docx showing the difference in species identification by MALDI-TOF MS and rMLST as well as the ST types of every isolate included in this study. [file 12866_2021_2388_MOESM2_ESM.docx]

Additional file 2. Species identification by MALDI-TOF MS and rMLST from whole genome sequencing data

| Isolate ID | MALDI-TOF MS | rMLST | | ST^1^ |
| --- | --- | --- | --- | --- |
|  | **Species** | **Species** | **rST** |  |
| 403121-15 | Acinetobacter calcoaceticus | Acinetobacter calcoaceticus | rST8700 | - |
| 501072-15 | Acinetobacter nosocomialis | Acinetobacter pittii | rST8770 | - |
| 403467-15 | Acinetobacter pittii | Acinetobacter pittii | rST8375 | - |
| 502814-15 | Acinetobacter sp. | Acinetobacter bereziniae | rST8307 | - |
| 503361-5-15 | Acinetobacter sp. | Acinetobacter bereziniae | rST8307 | - |
| 503777-15 | Acinetobacter sp. | Acinetobacter bereziniae | rST8307 | - |
| 503137-15 | Acinetobacter sp. | Acinetobacter bereziniae | rST8307 | - |
| 705884-17 | Citrobacter koseri | Citrobacter koseri | rST37964 | - |
| 808775-16 | Citrobacter koseri | Citrobacter koseri | rST37964 | - |
| 808887-16 | Citrobacter koseri | Citrobacter koseri | rST37964 | - |
| 707113-16 | Enterobacter aerogenes | Klebsiella aerogenes | rST44231 | ST-93 |
| 706868-17 | Enterobacter aerogenes | Klebsiella aerogenes | rST44228 | NA |
| 15897738 | Enterobacter cloacae | Enterobacter hormaechei | rST56609 | ST-511 |
| 26051866-KSLU | Enterobacter cloacae | Enterobacter cloacae | rST56625 | NA |
| 27007455-KSLU | Enterobacter cloacae | Enterobacter bugandensis | rST56642 | ST-921 |
| 706953-17 | Enterobacter cloacae | Enterobacter hormaechei | rST56646 | ST-1116 |
| 707208-3-17 | Enterobacter cloacae | Enterobacter hormaechei | rST56600 | ST-50 |
| 705498-12 | Escherichia coli | Escherichia coli | rST1645 | ST-428 |
| 703694-17 | Escherichia coli | Escherichia coli | rST30462 | ST-345 |
| 703692-17 | Escherichia coli | Escherichia coli | rST1920 | ST-73 |
| 108627-17 | Escherichia coli | Escherichia coli | rST1920 | ST-73 |
| 703629-17 | Escherichia coli | Escherichia coli | rST42569 | ST-537 |
| 108670-17 | Escherichia coli | Escherichia coli | rST1513 | ST-12 |
| 721296-16 | Escherichia coli | Escherichia coli | rST2011 | ST-744 |
| 700099-17 | Escherichia coli | Escherichia coli | rST2238 | ST-156 |
| 722306-16 | Escherichia coli | Escherichia coli | rST14798 | ST-254 |
| 700455-17 | Escherichia coli | Escherichia coli | rST30605 | ST-205 |
| 709006-16 | Escherichia coli | Escherichia coli | rST1503 | ST-131 |
| 705882-17 | Escherichia coli | Escherichia coli | rST2135 | ST-69 |
| 603351-17 | Escherichia coli | Escherichia coli | rST2185 | ST-95 |
| 705889-17 | Escherichia coli | Escherichia coli | rST1977 | ST-409 |
| 705801-17 | Escherichia coli | Escherichia coli | rST1676 | ST-405 |
| 19015727 | Escherichia coli | Escherichia coli | rST15332 | ST-2678 |
| 14929959 | Escherichia coli | Escherichia coli | rST1653 | ST-38 |
| ATCC-25922 | Escherichia coli | Escherichia coli | rST1923 | ST-73 |
| 706090-16 | Escherichia coli | Escherichia coli | rST59293 | ST-504 |
| 705963-16 | Escherichia coli | Escherichia coli | rST59294 | ST-404 |
| NCTC-13846 | Escherichia coli | Escherichia coli | rST30509 | ST-457 |
| KP-37-MCR-2-18 | Escherichia coli | Escherichia coli | rST1985 | ST-167 |
| 707671-17 | Escherichia coli | Escherichia coli | rST15570 | ST-10 |
| 719645-16 | Escherichia coli | Escherichia coli | rST2238 | ST-156 |
| 720516-15 | Hafnia alvei | Hafnia alvei | rST39580 | - |
| 703990-17 | Hafnia alvei | Hafnia alvei | rST39580 | - |
| 400640-17 | Hafnia alvei | Hafnia alvei | rST39583 | - |
| 501588-16 | Hafnia alvei | Hafnia alvei | rST39578 | - |
| 700538-16 | Hafnia alvei | Hafnia alvei | rST39588 | - |
| 606807-16 | Hafnia alvei | Hafnia alvei | rST39588 | - |
| 606809-16 | Hafnia alvei | Hafnia alvei | rST39588 | - |
| 500377-16 | Hafnia alvei | Hafnia alvei | rST39578 | - |
| 500445-16 | Hafnia alvei | Hafnia alvei | rST39578 | - |
| 504918-16 | Hafnia alvei | Hafnia alvei | rST39578 | - |
| 502060-16 | Hafnia alvei | Hafnia alvei | rST39578 | - |
| 605267-16 | Hafnia alvei | Hafnia alvei | rST39590 | - |
| 503671-16 | Hafnia alvei | Hafnia alvei | rST39578 | - |
| 501687-17 | Hafnia alvei | Hafnia alvei | rST39590 | - |
| 26057331-KSLU | Hafnia alvei | Hafnia alvei | rST39586 | - |
| 706508-16 | Klebsiella oxytoca | Klebsiella michiganensis | rST65600 | ST-253 |
| 500137-17 | Klebsiella oxytoca | Klebsiella oxytoca | rST63220 | ST-226 |
| 501450-17 | Klebsiella oxytoca | Klebsiella oxytoca | rST60014 | ST-176 |
| 402006-2-12 | Klebsiella pneumoniae | Klebsiella pneumoniae | rST31218 | ST-512 |
| 401433-14 | Klebsiella pneumoniae | Klebsiella pneumoniae | rST31218 | ST-512 |
| 809097-16 | Klebsiella pneumoniae | Klebsiella pneumoniae | rST43360 | ST-2982 |
| 809156-16 | Klebsiella pneumoniae | Klebsiella pneumoniae | rST43358 | ST-1825 |
| 113179-17 | Klebsiella pneumoniae | Klebsiella pneumoniae | rST43643 | ST-202 |
| 802208-17 | Klebsiella pneumoniae | Klebsiella pneumoniae | rST43358 | ST-1825 |
| 18701876 | Klebsiella pneumoniae | Klebsiella pneumoniae | rST31218 | ST-512 |
| 16003084 | Klebsiella pneumoniae | Klebsiella pneumoniae | rST43294 | ST-101 |
| 20038016 | Klebsiella pneumoniae | Klebsiella pneumoniae | rST31218 | ST-512 |
| 19852760 | Klebsiella pneumoniae | Klebsiella pneumoniae | rST19266 | ST-2502 |
| 19221887 | Klebsiella pneumoniae | Klebsiella quasipneumoniae | rST58581 | ST-2742 |
| 18111299 | Klebsiella pneumoniae | Klebsiella pneumoniae | rST31218 | ST-512 |
| 13892823 | Klebsiella pneumoniae | Klebsiella pneumoniae | rST31218 | ST-512 |
| 16020166 | Klebsiella pneumoniae | Klebsiella pneumoniae | rST19202 | ST-15 |
| 14414149 | Klebsiella pneumoniae | Klebsiella pneumoniae | rST31218 | ST-512 |
| 404507-16 | Klebsiella pneumoniae | Klebsiella pneumoniae | rST19275 | ST-307 |
| 808927-16 | Klebsiella pneumoniae | Klebsiella pneumoniae | rST18944 | ST-231 |
| 808922-16 | Klebsiella pneumoniae | Klebsiella pneumoniae | rST19272 | ST-36 |
| 26048671-KSLU | Klebsiella pneumoniae | Klebsiella pneumoniae | rST19208 | ST-14 |
| 800138-16 | Klebsiella pneumoniae | Klebsiella pneumoniae | rST19202 | ST-15 |
| 703693-17 | Morganella morganii | Morganella morganii | rST39714 | - |
| 501167-17 | Morganella morganii | Morganella morganii | rST39711 | - |
| 801470 -16 | Morganella morganii | Morganella morganii | rST39703 | - |
| ATCC-25933 | Proteus mirabilis | Proteus mirabilis | rST37030 | - |
| 715378-16 | Proteus mirabilis | Proteus mirabilis | rST37037 | - |
| 703689-17 | Proteus mirabilis | Proteus vulgaris | rST37046 | - |
| 501533-08 | Pseudomonas aeruginosa | Pseudomonas aeruginosa | rST20592 | ST-235 |
| 703687-17 | Pseudomonas aeruginosa | Pseudomonas aeruginosa | rST20735 | ST-1763 |
| 400761-17 | Pseudomonas aeruginosa | Pseudomonas aeruginosa | rST20633 | ST-395 |
| 703547-17 | Pseudomonas aeruginosa | Pseudomonas aeruginosa | rST15625 | ST-1405 |
| 17674722 | Pseudomonas aeruginosa | Pseudomonas aeruginosa | rST18939 | NA |
| 19628438 | Pseudomonas aeruginosa | Pseudomonas aeruginosa | rST20609 | ST-644 |
| 19953214 | Pseudomonas aeruginosa | Pseudomonas aeruginosa | rST20838 | ST-260 |
| ATCC-27853 | Pseudomonas aeruginosa | Pseudomonas aeruginosa | rST20748 | ST-155 |
| 27004390 -KSLU | Pseudomonas aeruginosa | Pseudomonas aeruginosa | rST20964 | ST-244 |
| 27004418-KSLU | Pseudomonas aeruginosa | Pseudomonas aeruginosa | rST20646 | ST-760 |
| 26057596-KSLU | Serratia marcescens | Serratia marcescens | rST19799 | - |
| 716683-16 | Serratia marcescens | Serratia marcescens | rST19786 | - |

*NA, Sequence type not assigned.*

*^1^ST schemes do not exist for Acinetobacter, Citrobacter, Hafnia, Morganella, Proteus and Serratia genus.*
