# Supplementary material for: Colistin resistance in Gram-negative bacteria analysed by five phenotypic assays and inference of the underlying genomic mechanisms
Source: BMC Microbiol. 2021 Nov 20;21:321. doi: 10.1186/s12866-021-02388-8 (PMC8605564; doi:10.1186/s12866-021-02388-8)
Supplement: Supplementary file 3 — Additional file 3. Klebsiella pneumoniae protein sequence alignments.pdf showing the protein sequences alignments of all colistin resistance-related proteins analyzed in this study. [file 12866_2021_2388_MOESM3_ESM.pdf]

Alignment: D:\Documentos BASILEA\AMR LAB\Colistin resistance project\Genes\K. pneumoniae\mgrB\K
Seaview [blocks=10 fontsize=10 A4] on Wed Jun 03 20:32:06 2020

```
                         1
ref_MH368667/1-48        VKKLRWVLLI VIIAGCLLLW TQMLNVMCDQ DVQFFSGICT INKFIPWE
113179-17_S_mgrB/1-48    VKKLRWVLLI VIIAGCLLLW TQMLNVMCDQ DVQFFSGICT INKFIPWX
809097-16_S_mgrB/1-48    VKKLRWVLLI VIIAGCLLLW TQMLNVMCDQ DVQFFSGICT INKFIPWX
13892823_S_mgrB/1-48     VKKLRWVLLI VIIAGCLLLW TQMLNVMCDQ DVQFFSGICT INKFIPWX
14414149_S_mgrB/1-48     VKKLRWVLLI VIIAGCLLLW TQMLNVMCDQ DVQFFSGICT INKFIPWX
16020166_S_mgrB/1-48     VKKLRWVLLI VIIAGCLLLW TQMLNVMCDQ DVQFFSGICT INKFIPWX
18111299_S_mgrB/1-48     VKKLRWVLLI VIIAGCLLLW TQMLNVMCDQ DVQFFSGICT INKFIPWX
19221887_S_mgrB/1-48     VKKLRWVLLI VIIAGCLLLW TQMLNVMCDQ DVQFFSGICT INKFIPWX
808922-16_S_mgrB/1-48    VKKLRWVLLI VIIAGCLLLW TQMLNVMCDQ DVQFFSGICT INKFIPWX
16003084_R_mgrB/1-48     VKKLRWVLLI VIIAGCLLLW TQMLNVMSDQ DVQFFSGICT INKFIPWX
18701876_R_mgrB/1-48     VKKLRWVLLI VIIAGCLLLW TQMLNVMCDQ DVQFFSGIGT INKFIPWX
20038016_R_mgrB/1-48     VKKLRWVLLI VIIAGCLLLW TQMLNVMCDQ DVQFFSGIYT INKFIPWX
26048671_R_mgrB/1-48     VKKLRWVLLI VIIAGCLLLW TQMLNVMCDQ DVQFFSGICT XNKFIPWX
401433-14_R_mgrB/1-48    VKKLRWVLLI VIIAGCLLLW TQMLNVMCDX DVQFFSGICT INKFIPWX
402006-2-12_R_mgrB/1-48  VKXLRWVLLI VIIAGCLLLW TQMLNVMCDQ DVQFFSGICT INKFIPWX
404507-16_R_mgrB/1-48    VKKLRWVLLI VIIAGCLLLW TQMLNVMCDQ NVQFFSGICT INKFIPWX
800138-16_R_mgrB/1-48    VKKLRWVLLI VIIAGCLLLW TQMLNVMCDQ DVQFFSGICT XNKFIPWX
802208-17_R_mgrB/1-46    V--XRWVLLI VIIAGCLLLW TQMLNVMCDQ DVQFFSGICT INKFIPWX
808927-16_R_mgrB/1-48    VKKLRWVXLI VIIAGCLLLW TQMLNVMCDQ DVQFFSGICT INKFIPWX
809156-16_R_mgrB/1-48    VKKLRWVLLI VIIAGCLLLW TQMLNVMCDQ DVQFFSGICT INKFIPWX
```

Alignment: D:\Documentos BASILEA\AMR LAB\Colistin resistance project\Genes\K. pneumoniae\PhoP\K
Seaview [blocks=10 fontsize=10 A4] on Wed Jun 03 19:38:29 2020

```
                                  1
113179-17_04093_S_phoP/1-224      MRVLVVEDNA LLRHHLKVQL QELGHQVDAA EDAREADYYL GEHLPDIAIV
16020166_04232_S_phoP/1-224       MRVLVVEDNA LLRHHLKVQL QELGHQVDAA EDAREADYYL GEHLPDIAIV
14414149_01061_S_phoP/1-224       MRVLVVEDNA LLRHHLKVQL QELGHQVDAA EDAREADYYL GEHLPDIAIV
18111299_04293_S_phoP/1-224       MRVLVVEDNA LLRHHLKVQL QELGHQVDAA EDAREADYYL GEHLPDIAIV
19221887_02546_S_phoP/1-224       MRVLVVEDNA LLRHHLKVQL QELGHQVDAA EDAKEADYYL GEHLPDIAIV
809097-16_S_phoP/1-224            MRVLVVEDNA LLRHHLKVQL QELGHQVDAA EDAREADYYL GEHLPDIAIV
808922-16_S_phoP/1-224            MRVLVVEDNA LLRHHLKVQL QELGHQVDAA EDAREADYYL GEHLPDIAIV
13892823_00475_S_phoP/1-224       MRVLVVEDNA LLRHHLKVQL QELGHQVDAA EDAREADYYL GEHLPDIAIV
809156-16_R_phoP/1-224            MRVLVVEDNA LLRHHLKVQL QELGHQVDAA EDAREADYYL GEHLPDIAIV
26048671-KSLU_03006_R_phoP/1-224  MRVLVVEDNA LLRHHLKVQL QELGHQVDAA EDAREADYYL GEHLPDIAIV
401433-14_00643_R_phoP/1-224      MRVLVVEDNA LLRHHLKVQL QELGHQVDAA EDAREADYYL GEHLPDIAIV
402006-2-12_00289_R_phoP/1-224    MRVLVVEDNA LLRHHLKVQL QELGHQVDAA EDAREADYYL GEHLPDIAIV
404507-16_02523_R_phoP/1-224      MRVLVVEDNA LLRHHLKVQL QELGHQVDAA EDAREADYYL GEHLPDIAIV
800138-16_04647_R_phoP/1-224      MRVLVVEDNA LLRHHLKVQL QELGHQVDAA EDAREADYYL GEHLPDIAIV
802208-17_04555_R_phoP/1-224      MRVLVVEDNA LLRHHLKVQL QELGHQVDAA EDAREADYYL GEHLPDIAIV
808927-16_04543_R_phoP/1-224      MRVLVVEDNA LLRHHLKVQL QELGHQVDAA EDAREADYYL GEHLPDIAIV
16003084_01266_R_phoP/1-224       MRVLVVEDNA LLRHHLKVQL QELGHQVDAA EDAREADYYL GEHLPDIAIV
18701876_00836_R_phoP/1-224       MRVLVVEDNA LLRHHLKVQL QELGHQVDAA EDAREADYYL GEHLPDIAIV
19852760_02288_R_phoP/1-224       MRVLVVEDNA LLRHHLKVQL QELGHQVDAA EDAREADYYL GEHLPDIAIV
20038016-KSLU_01415_R_phoP/1-224  MRVLVVEDNA LLRHHLKVQL QELGHQVDAA EDAREADYYL GEHLPDIAIV

                                 51
113179-17_04093_S_phoP/1-224      DLGLPDEDGL SLIRRWRSHD VSLPVLVLTA REGWQDKVEV LSAGADDYVT
16020166_04232_S_phoP/1-224       DLGLPDEDGL SLIRRWRSHD VSLPVLVLTA REGWQDKVEV LSAGADDYVT
14414149_01061_S_phoP/1-224       DLGLPDEDGL SLIRRWRSHD VSLPVLVLTA REGWQDKVEV LSAGADDYVT
18111299_04293_S_phoP/1-224       DLGLPDEDGL SLIRRWRSHD VSLPVLVLTA REGWQDKVEV LSAGADDYVT
19221887_02546_S_phoP/1-224       DLGLPDEDGL SLIRRWRSHD VSLPVLVLTA REGWQDKVEV LSAGADDYVT
809097-16_S_phoP/1-224            DLGLPDEDGL SLIRRWRSHD VSLPVLVLTA REGWQDKVEV LSAGADDYVT
808922-16_S_phoP/1-224            DLGLPDEDGL SLIRRWRSHD VSLPVLVLTA REGWQDKVEV LSAGADDYVT
13892823_00475_S_phoP/1-224       DLGLPDEDGL SLIRRWRSHD VSLPVLVLTA REGWQDKVEV LSAGADDYVT
809156-16_R_phoP/1-224            DLGLPDEDGL SLIRRWRSHD VSLPVLVLTA REGWQDKVEV LSAGADDYVT
26048671-KSLU_03006_R_phoP/1-224  DLGLPDEDGL SLIRRWRSHD VSLPVLVLTA REGWQDKVEV LSAGADDYVT
401433-14_00643_R_phoP/1-224      DLGLPDEDGL SLIRRWRSHD VSLPVLVLTA REGWQDKVEV LSAGADDYVT
402006-2-12_00289_R_phoP/1-224    DLGLPDEDGL SLIRRWRSHD VSLPVLVLTA REGWQDKVEV LSAGADDYVT
404507-16_02523_R_phoP/1-224      DLGLPDEDGL SLIRRWRSHD VSLPVLVLTA REGWQDKVEV LSAGADDYVT
800138-16_04647_R_phoP/1-224      DLGLPDEDGL SLIRRWRSHD VSLPVLVLTA REGWQDKVEV LSAGADDYVT
802208-17_04555_R_phoP/1-224      DLGLPDEDGL SLIRRWRSHD VSLPVLVLTA REGWQDKVEV LSAGADDYVT
808927-16_04543_R_phoP/1-224      DLGLPDEDGL SLIRRWRSHD VSLPVLVLTA REGWQDKVEV LSAGADDYVT
16003084_01266_R_phoP/1-224       DLGLPDEDGL SLIRRWRSHD VSLPVLVLTA REGWQDKVEV LSAGADDYVT
18701876_00836_R_phoP/1-224       DLGLPDEDGL SLIRRWRSHD VSLPVLVLTA REGWQDKVEV LSAGADDYVT
19852760_02288_R_phoP/1-224       DLGLPDEDGL SLIRRWRSHD VSLPVLVLTA REGWQDKVEV LSAGADDYVT
20038016-KSLU_01415_R_phoP/1-224  DLGLPDEDGL SLIRRWRSHD VSLPVLVLTA REGWQDKVEV LSAGADDYVT

                                101
113179-17_04093_S_phoP/1-224      KPFHIEEVAA RMQALLRRNS GLASQVISLP PFQVDLSRRE LSVNDQPIKL
16020166_04232_S_phoP/1-224       KPFHIEEVAA RMQALLRRNS GLASQVISLP PFQVDLSRRE LSVNDQPIKL
14414149_01061_S_phoP/1-224       KPFHIEEVAA RMQALLRRNS GLASQVISLP PFQVDLSRRE LSVNDQPIKL
18111299_04293_S_phoP/1-224       KPFHIEEVAA RMQALLRRNS GLASQVISLP PFQVDLSRRE LSVNDQPIKL
19221887_02546_S_phoP/1-224       KPFHIEEVAA RMQALLRRNS GLASQVISLP PFQVDLSRRE LSVNDQPIKL
809097-16_S_phoP/1-224            KPFHIEEVAA RMQALLRRNS GLASQVISLP PFQVDLSRRE LSVNDQPIKL
808922-16_S_phoP/1-224            KPFHIEEVAA RMQALLRRNS GLASQVISLP PFQVDLSRRE LSVNDQPIKL
13892823_00475_S_phoP/1-224       KPFHIEEVAA RMQALLRRNS GLASQVISLP PFQVDLSRRE LSVNDQPIKL
809156-16_R_phoP/1-224            KPFHIEEVAA RMQALLRRNS GLASQVISLP PFQVDLSRRE LSVNDQPIKL
26048671-KSLU_03006_R_phoP/1-224  KPFHIEEVAA RMQALLRRNS GLASQVISLP PFQVDLSRRE LSVNDQPIKL
401433-14_00643_R_phoP/1-224      KPFHIEEVAA RMQALLRRNS GLASQVISLP PFQVDLSRRE LSVNDQPIKL
402006-2-12_00289_R_phoP/1-224    KPFHIEEVAA RMQALLRRNS GLASQVISLP PFQVDLSRRE LSVNDQPIKL
404507-16_02523_R_phoP/1-224      KPFHIEEVAA RMQALLRRNS GLASQVISLP PFQVDLSRRE LSVNDQPIKL
800138-16_04647_R_phoP/1-224      KPFHIEEVAA RMQALLRRNS GLASQVISLP PFQVDLSRRE LSVNDQPIKL
802208-17_04555_R_phoP/1-224      KPFHIEEVAA RMQALLRRNS GLASQVISLP PFQVDLSRRE LSVNDQPIKL
808927-16_04543_R_phoP/1-224      KPFHIEEVAA RMQALLRRNS GLASQVISLP PFQVDLSRRE LSVNDQPIKL
16003084_01266_R_phoP/1-224       KPFHIEEVAA RMQALLRRNS GLASQVISLP PFQVDLSRRE LSVNDQPIKL
18701876_00836_R_phoP/1-224       KPFHIEEVAA RMQALLRRNS GLASQVISLP PFQVDLSRRE LSVNDQPIKL
19852760_02288_R_phoP/1-224       KPFHIEEVAA RMQALLRRNS GLASQVISLP PFQVDLSRRE LSVNDQPIKL
20038016-KSLU_01415_R_phoP/1-224  KPFHIEEVAA RMQALLRRNS GLASQVISLP PFQVDLSRRE LSVNDQPIKL
```

```
                                           151
113179-17_04093_S_phoP/1-224        TAFEYTIMET LIRNRGKVVS KDSLMLQLYP DAELRESHTI DVLMGRLRKK
16020166_04232_S_phoP/1-224         TAFEYTIMET LIRNRGKVVS KDSLMLQLYP DAELRESHTI DVLMGRLRKK
14414149_01061_S_phoP/1-224         TAFEYTIMET LIRNRGKVVS KDSLMLQLYP DAELRESHTI DVLMGRLRKK
18111299_04293_S_phoP/1-224         TAFEYTIMET LIRNRGKVVS KDSLMLQLYP DAELRESHTI DVLMGRLRKK
19221887_02546_S_phoP/1-224         TAFEYTIMET LIRNRGKVVS KDSLMLQLYP DAELRESHTI DVLMGRLRKK
809097-16_S_phoP/1-224              TAFEYTIMET LIRNRGKVVS KDSLMLQLYP DAELRESHTI DVLMGRLRKK
808922-16_S_phoP/1-224              TAFEYTIMET LIRNRGKVVS KDSLMLQLYP DAELRESHTI DVLMGRLRKK
13892823_00475_S_phoP/1-224         TAFEYTIMET LIRNRGKVVS KDSLMLQLYP DAELRESHTI DVLMGRLRKK
809156-16_R_phoP/1-224              TAFEYTIMET LIRNRGKVVS KDSLMLQLYP DAELRESHTI DVLMGRLRKK
26048671-KSLU_03006_R_phoP/1-224    TAFEYTIMET LIRNRGKVVS KDSLMLQLYP DAELRESHTI DVLMGRLRKK
401433-14_00643_R_phoP/1-224        TAFEYTIMET LIRNRGKVVS KDSLMLQLYP DAELRESHTI DVLMGRLRKK
402006-2-12_00289_R_phoP/1-224      TAFEYTIMET LIRNRGKVVS KDSLMLQLYP DAELRESHTI DVLMGRLRKK
404507-16_02523_R_phoP/1-224        TAFEYTIMET LIRNRGKVVS KDSLMLQLYP DAELRESHTI DVLMGRLRKK
800138-16_04647_R_phoP/1-224        TAFEYTIMET LIRNRGKVVS KDSLMLQLYP DAELRESHTI DVLMGRLRKK
802208-17_04555_R_phoP/1-224        TAFEYTIMET LIRNRGKVVS KDSLMLQLYP DAELRESHTI DVLMGRLRKK
808927-16_04543_R_phoP/1-224        TAFEYTIMET LIRNRGKVVS KDSLMLQLYP DAELRESHTI DVLMGRLRKK
16003084_01266_R_phoP/1-224         TAFEYTIMET LIRNRGKVVS KDSLMLQLYP DAELRESHTI DVLMGRLRKK
18701876_00836_R_phoP/1-224         TAFEYTIMET LIRNRGKVVS KDSLMLQLYP DAELRESHTI DVLMGRLRKK
19852760_02288_R_phoP/1-224         TAFEYTIMET LIRNRGKVVS KDSLMLQLYP DAELRESHTI DVLMGRLRKK
20038016-KSLU_01415_R_phoP/1-224    TAFEYTIMET LIRNRGKVVS KDSLMLQLYP DAELRESHTI DVLMGRLRKK
```

```
                                           201
113179-17_04093_S_phoP/1-224        IQAEYPQDVI TTVRGQGYLF ELR*
16020166_04232_S_phoP/1-224         IQAEYPQDVI TTVRGQGYLF ELR*
14414149_01061_S_phoP/1-224         IQAEYPQDVI TTVRGQGYLF ELR*
18111299_04293_S_phoP/1-224         IQAEYPQDVI TTVRGQGYLF ELR*
19221887_02546_S_phoP/1-224         IQAEYPQDVI TTVRGQGYLF ELR*
809097-16_S_phoP/1-224              IQAEYPQDVI TTVRGQGYLF ELR*
808922-16_S_phoP/1-224              IQAEYPQDVI TTVRGQGYLF ELR*
13892823_00475_S_phoP/1-224         IQAEYPQDVI TTVRGQGYLF ELR*
809156-16_R_phoP/1-224              IQAEYPQDVI TTVRGQGYLF ELR*
26048671-KSLU_03006_R_phoP/1-224    IQAEYPQDVI TTVRGQGYLF ELR*
401433-14_00643_R_phoP/1-224        IQAEYPQDVI TTVRGQGYLF ELR*
402006-2-12_00289_R_phoP/1-224      IQAEYPQDVI TTVRGQGYLF ELR*
404507-16_02523_R_phoP/1-224        IQAEYPQDVI TTVRGQGYLF ELR*
800138-16_04647_R_phoP/1-224        IQAEYPQDVI TTVRGQGYLF ELR*
802208-17_04555_R_phoP/1-224        IQAEYPQDVI TTVRGQGYLF ELR*
808927-16_04543_R_phoP/1-224        IQAEYPQDVI TTVRGQGYLF ELR*
16003084_01266_R_phoP/1-224         IQAEYPQDVI TTVRGQGYLF ELR*
18701876_00836_R_phoP/1-224         IQAEYPQDVI TTVRGQGYLF ELR*
19852760_02288_R_phoP/1-224         IQAEYPQDVI TTVRGQGYLF ELR*
20038016-KSLU_01415_R_phoP/1-224    IQAEYPQDVI TTVRGQGYLF ELR*
```

Alignment: D:\Documentos BASILEA\AMR LAB\Colistin resistance project\Genes\K. pneumoniae\phoQ\K
Seaview [blocks=10 fontsize=10 A4] on Wed Jun 03 19:49:02 2020

	1				
113179-17_04092_S_phoQ/1-489	MKGLLRHIFP	LSLRVRFLLA	TAGVVLVLSL	AYGMVALVGY	SVSFDKTTFR
16020166_04233_S_phoQ/1-489	MKGLLRHIFP	LSLRVRFLLA	TAGVVLVLSL	AYGMVALVGY	SVSFDKTTFR
19221887_02545_S_phoQ/1-489	MKGLLRHIFP	LSLRVRFLLA	TAGVVLVLSL	AYGMVALVGY	SVSFDKTTFR
18111299_04292_S_phoQ/1-489	MKGLLRHIFP	LSLRVRFLLA	TAGVVLVLSL	AYGMVALVGY	SVSFDKTTFR
809097-16_05020_S_phoQ/1-489	MKGLLRHIFP	LSLRVRFLLA	TAGVVLVLSL	AYGMVALVGY	SVSFDKTTFR
808922-16_03828_S_phoQ/1-489	MKGLLRHIFP	LSLRVRFLLA	TAGVVLVLSL	AYGMVALVGY	SVSFDKTTFR
13892823_00476_S_phoQ/1-489	MKGLLRHIFP	LSLRVRFLLA	TAGVVLVLSL	AYGMVALVGY	SVSFDKTTFR
14414149_01062_S_phoQ/1-489	MKGLLRHIFP	LSLRVRFLLA	TAGVVLVLSL	AYGMVALVGY	SVSFDKTTFR
809156-16_05046_R_phoQ/1-489	MKGLLRHIFP	LSLRVRFLLA	TAGVVLVLSL	AYGMVALVGY	SVSFDKTTFR
26048671-KSLU_03005_R_phoQ/1-489	MKGLLRHIFP	LSLRVRFLLA	TAGVVLVLSL	AYGMVALVGY	SVSFDKTTFR
19852760_02287_R_phoQ/1-489	MKGLLRHIFP	LSLRVRFLLA	TAGVVLVLSL	AYGMVALVGY	SVSFDKTTFR
401433-14_00644_R_phoQ/1-489	MKGLLRHIFP	LSLRVRFLLA	TAGVVLVLSL	AYGMVALVGY	SVSFDKTTFR
18701876_00837_R_phoQ/1-489	MKGLLRHIFP	LSLRVRFLLA	TAGVVLVLSL	AYGMVALVGY	SVSFDKTTFR
808927-16_04544_R_phoQ/1-489	MKGLLRHIFP	LSLRVRFLLA	TAGVVLVLSL	AYGMVALVGY	SVSFDKTTFR
800138-16_04646_R_phoQ/1-489	MKGLLRHIFP	LSLRVRFLLA	TAGVVLVLSL	AYGMVALVGY	SVSFDKTTFR
802208-17_04556_R_phoQ/1-489	MKGLLRHIFP	LSLRVRFLLA	TAGVVLVLSL	AYGMVALVGY	SVSFDKTTFR
20038016-KSLU_01414_R_phoQ/1-489	MKGLLRHIFP	LSLRVRFLLA	TAGVVLVLSL	AYGMVALVGY	SVSFDKTTFR
404507-16_02522_R_phoQ/1-489	MKGLLRHIFP	LSLRVRFLLA	TAGVVLVLSL	AYGMVALVGY	SVSFDKTTFR
402006-2-12_00288_R_phoQ/1-489	MKGLLRHIFP	LSLRVRFLLA	TAGVVLVLSL	AYGMVALVGY	SVSFDKTTFR
16003084_01265_R_phoQ/1-489	MKGLLRHIFP	LSLRVRFLLA	TAGVVLVLSL	AYGMVALVGY	SVSFDKTTFR

	51				
113179-17_04092_S_phoQ/1-489	LLRGESNLFY	MLARWENGAI	DVDIPENLNM	ESPTVTLIYD	EQGKLLWAQR
16020166_04233_S_phoQ/1-489	LLRGESNLFY	MLARWENGAI	DVDIPENLNM	ESPTVTLIYD	EQGKLLWAQR
19221887_02545_S_phoQ/1-489	LLRGESNLFY	MLAKWENGAI	DVDIPENLNM	ESPTVTLIYD	EKGKLLWAQR
18111299_04292_S_phoQ/1-489	LLRGESNLFY	MLARWENGAI	DVDIPENLNM	ESPTVTLIYD	EQGKLLWAQR
809097-16_05020_S_phoQ/1-489	LLRGESNLFY	MLARWENGAI	DVDIPENLNM	ESPTVTLIYD	EQGKLLWAQR
808922-16_03828_S_phoQ/1-489	LLRGESNLFY	MLARWENGAI	DVDIPENLNM	ESPTVTLIYD	EQGKLLWAQR
13892823_00476_S_phoQ/1-489	LLRGESNLFY	MLARWENGAI	DVDIPENLNM	ESPTVTLIYD	EQGKLLWAQR
14414149_01062_S_phoQ/1-489	LLRGESNLFY	MLARWENGAI	DVDIPENLNM	ESPTVTLIYD	EQGKLLWAQR
809156-16_05046_R_phoQ/1-489	LLRGESNLFY	MLARWENGAI	DVDIPENLNM	ESPTVTLIYD	EQGKLLWAQR
26048671-KSLU_03005_R_phoQ/1-489	LLRGESNLFY	MLARWENGAI	DVDIPENLNM	ESPTVTLIYD	EQGKLLWAQR
19852760_02287_R_phoQ/1-489	LLRGESNLFY	MLARWENGAI	DVDIPENLNM	ESPTVTLIYD	EQGKLLWAQR
401433-14_00644_R_phoQ/1-489	LLRGESNLFY	MLARWENGAI	DVDIPENLNM	ESPTVTLIYD	EQGKLLWAQR
18701876_00837_R_phoQ/1-489	LLRGESNLFY	MLARWENGAI	DVDIPENLNM	ESPTVTLIYD	EQGKLLWAQR
808927-16_04544_R_phoQ/1-489	LLRGESNLFY	MLARWENGAI	DVDIPENLNM	ESPTVTLIYD	EQGKLLWAQR
800138-16_04646_R_phoQ/1-489	LLRGESNLFY	MLARWENGAI	DVDIPENLNM	ESPTVTLIYD	EQGKLLWAQR
802208-17_04556_R_phoQ/1-489	LLRGESNLFY	MLARWENGAI	DVDIPENLNM	ESPTVTLIYD	EQGKLLWAQR
20038016-KSLU_01414_R_phoQ/1-489	LLRGESNLFY	MLARWENGAI	DVDIPENLNM	ESPTVTLIYD	EQGKLLWAQR
404507-16_02522_R_phoQ/1-489	LLRGESNLFY	MLARWENGAI	DVDIPENLNM	ESPTVTLIYD	EQGKLLWAQR
402006-2-12_00288_R_phoQ/1-489	LLRGESNLFY	MLARWENGAI	DVDIPENLNM	ESPTVTLIYD	EQGKLLWAQR
16003084_01265_R_phoQ/1-489	LLRGESNLFY	MLARWENGAI	DVDIPENLNM	ESPTVTLIYD	EQGKLLWAQR

	101				
113179-17_04092_S_phoQ/1-489	DVPWLAKRIQ	PEWLKRNGFH	EIEADVDSSS	MLLRNNHEIQ	EQLDAIREQG
16020166_04233_S_phoQ/1-489	DVPWLAKRIQ	PEWLKRNGFH	EIEADVDSSS	MLLRNNHEIQ	EQLDAIREQG
19221887_02545_S_phoQ/1-489	DVPWLTKRIQ	PDWLKRNGFH	EIEADVDSSS	MLLRNNHEVQ	EQLDAIREQG
18111299_04292_S_phoQ/1-489	DVPWLAKRIQ	PEWLKRNGFH	EIEADVDSSS	MLLRNNHEIQ	EQLDAIREQG
809097-16_05020_S_phoQ/1-489	DVPWLAKRIQ	PEWLKRNGFH	EIEADVDSSS	MLLRNNHEIQ	EQLDAIREQG
808922-16_03828_S_phoQ/1-489	DVPWLAKRIQ	PEWLKRNGFH	EIEADVDSSS	MLLRNNHEIQ	EQLDAIREQG
13892823_00476_S_phoQ/1-489	DVPWLAKRIQ	PEWLKRNGFH	EIEADVDSSS	MLLRNNHEIQ	EQLDAIREQG
14414149_01062_S_phoQ/1-489	DVPWLAKRIQ	PEWLKRNGFH	EIEADVDSSS	MLLRNNHEIQ	EQLDAIREQG
809156-16_05046_R_phoQ/1-489	DVPWLAKRIQ	PEWLKRNGFH	EIEADVDSSS	MLLRNNHEIQ	EQLDAIREQG
26048671-KSLU_03005_R_phoQ/1-489	DVPWLAKRIQ	PEWLKRNGFH	EIEADVDSSS	MLLRNNHEIQ	EQLDAIREQG
19852760_02287_R_phoQ/1-489	DVPWLAKRIQ	PEWLKRNGFH	EIEADVDSSS	MLLRNNHEIQ	EQLDAIREQG
401433-14_00644_R_phoQ/1-489	DVPWLAKRIQ	PEWLKRNGFH	EIEADVDSSS	MLLRNNHEIQ	EQLDAIREQG
18701876_00837_R_phoQ/1-489	DVPWLAKRIQ	PEWLKRNGFH	EIEADVDSSS	MLLRNNHEIQ	EQLDAIREQG
808927-16_04544_R_phoQ/1-489	DVPWLAKRIQ	PEWLKRNGFH	EIEADVDSSS	MLLRNNHEIQ	EQLDAIREQG
800138-16_04646_R_phoQ/1-489	DVPWLAKRIQ	PEWLKRNGFH	EIEADVDSSS	MLLRNNHEIQ	EQLDAIREQG
802208-17_04556_R_phoQ/1-489	DVPWLAKRIQ	PEWLKRNGFH	EIEADVDSSS	MLLRNNHEIQ	EQLDAIREQG
20038016-KSLU_01414_R_phoQ/1-489	DVPWLAKRIQ	PEWLKRNGFH	EIEADVDSSS	MLLRNNHEIQ	EQLDAIREQG
404507-16_02522_R_phoQ/1-489	DVPWLAKRIQ	PEWLKRNGFH	EIEADVDSSS	MLLRNNHEIQ	EQLDAIREQG
402006-2-12_00288_R_phoQ/1-489	DVPWLAKRIQ	PEWLKRNGFH	EIEADVDSSS	MLLRNNHEIQ	EQLDAIREQG
16003084_01265_R_phoQ/1-489	DVPWLAKRIQ	PEWLKRNGFH	EIEADVDSSS	MLLRNNHEIQ	EQLDAIREQG

```
                                      151
113179-17_04092_S_phoQ/1-489          DDSEMTHSVA INLYPATSKM PQLSIVVVDT IPVELKRSYM VWSWFVYVLA
16020166_04233_S_phoQ/1-489           DDSEMTHSVA INLYPATSKM PQLSIVVVDT IPVELKRSYM VWSWFVYVLA
19221887_02545_S_phoQ/1-489           DDSEMTHSVA INFYPATSKM PQLSIVVVDT IPVELKRSYM VWSWFIYVLA
18111299_04292_S_phoQ/1-489           DDSEMTHSVA INLYPATSKM PQLSIVVVDT IPVELKRSYM VWSWFVYVLA
809097-16_05020_S_phoQ/1-489          DDSEMTHSVA INLYPATSKM PQLSIVVVDT IPVELKRSYM VWSWFVYVLA
808922-16_03828_S_phoQ/1-489          DDSEMTHSVA INLYPATSKM PQLSIVVVDT IPVELKRSYM VWSWFVYVLA
13892823_00476_S_phoQ/1-489           DDSEMTHSVA INLYPATSKM PQLSIVVVDT IPVELKRSYM VWSWFVYVLA
14414149_01062_S_phoQ/1-489           DDSEMTHSVA INLYPATSKM PQLSIVVVDT IPVELKRSYM VWSWFVYVLA
809156-16_05046_R_phoQ/1-489          DDSEMTHSVA INLYPATSKM PQLSIVVVDT IPVELKRSYM VWSWFVYVLA
26048671-KSLU_03005_R_phoQ/1-489      DDSEMTHSVA INLYPATSKM PQLSIVVVDT IPVELKRSYM VWSWFVYVLA
19852760_02287_R_phoQ/1-489           DDSEMTHSVA INLYPATSKM PQLSIVVVDT IPVELKRSYM VWSWFVYVLA
401433-14_00644_R_phoQ/1-489          DDSEMTHSVA INLYPATSKM PQLSIVVVDT IPVELKRSYM VWSWFVYVLA
18701876_00837_R_phoQ/1-489           DDSEMTHSVA INLYPATSKM PQLSIVVVDT IPVELKRSYM VWSWFVYVLA
808927-16_04544_R_phoQ/1-489          DDSEMTHSVA INLYPATSKM PQLSIVVVDT IPVELKRSYM VWSWFVYVLA
800138-16_04646_R_phoQ/1-489          DDSEMTHSVA INLYPATSKM PQLSIVVVDT IPVELKRSYM VWSWFVYVLA
802208-17_04556_R_phoQ/1-489          DDSEMTHSVA INLYPATSKM PQLSIVVVDT IPVELKRSYM VWSWFVYVLA
20038016-KSLU_01414_R_phoQ/1-489      DDSEMTHSVA INLYPATSKM PQLSIVVVDT IPVELKRSYM VWSWFVYVLA
404507-16_02522_R_phoQ/1-489          DDSEMTHSVA INLYPATSKM PQLSIVVVDT IPVELKRSYM VWSWFVYVLA
402006-2-12_00288_R_phoQ/1-489        DDSEMTHSVA INLYPATSKM PQLSIVVVDT IPVELKRSYM VWSWFVYVLA
16003084_01265_R_phoQ/1-489           DDSEMTHSVA INLYPATSKM PQLSIVVVDT IPVELKRSYM VWSWFVYVLA

                                      201
113179-17_04092_S_phoQ/1-489          ANLLLVIPLL WVAAWWSLRP IESLAKEVRE LEEHHREKLN PNTTRELTRL
16020166_04233_S_phoQ/1-489           ANLLLVIPLL WVAAWWSLRP IESLAKEVRE LEEHHREKLN PNTTRELTRL
19221887_02545_S_phoQ/1-489           ANLLLVIPLL WVAAWWSLRP IESLAKEVRE LEEHHREKLN PNTTRELTRL
18111299_04292_S_phoQ/1-489           ANLLLVIPLL WVAAWWSLRP IESLAKEVRE LEEHHREKLN PNTTRELTRL
809097-16_05020_S_phoQ/1-489          ANLLLVIPLL WVAAWWSLRP IESLAKEVRE LEEHHREKLN PNTTRELTRL
808922-16_03828_S_phoQ/1-489          ANLLLVIPLL WVAAWWSLRP IESLAKEVRE LEEHHREKLN PNTTRELTRL
13892823_00476_S_phoQ/1-489           ANLLLVIPLL WVAAWWSLRP IESLAKEVRE LEEHHREKLN PNTTRELTRL
14414149_01062_S_phoQ/1-489           ANLLLVIPLL WVAAWWSLRP IESLAKEVRE LEEHHREKLN PNTTRELTRL
809156-16_05046_R_phoQ/1-489          ANLLLVIPLL WVAAWWSLRP IESLAKEVRE LEEHHREKLN PNTTRELTRL
26048671-KSLU_03005_R_phoQ/1-489      ANLLLVIPLL WVAAWWSLRP IESLAKEVRE LEEHHREKLN PNTTRELTRL
19852760_02287_R_phoQ/1-489           ANLLLVIPLL WVAAWWSLRP IESLAKEVRE LEEHHREKLN PNTTRELTRL
401433-14_00644_R_phoQ/1-489          ANLLLVIPLL WVAAWWSLRP IESLAKEVRE LEEHHREKLN PNTTRELTRL
18701876_00837_R_phoQ/1-489           ANLLLVIPLL WVAAWWSLRP IESLAKEVRE LEEHHREKLN PNTTRELTRL
808927-16_04544_R_phoQ/1-489          ANLLLVIPLL WVAAWWSLRP IESLAKEVRE LEEHHREKLN PNTTRELTRL
800138-16_04646_R_phoQ/1-489          ANLLLVIPLL WVAAWWSLRP IESLAKEVRE LEEHHREKLN PNTTRELTRL
802208-17_04556_R_phoQ/1-489          ANLLLVIPLL WVAAWWSLRP IESLAKEVRE LEEHHREKLN PNTTRELTRL
20038016-KSLU_01414_R_phoQ/1-489      ANLLLVIPLL WVAAWWSLRP IESLAKEVRE LEEHHREKLN PNTTRELTRL
404507-16_02522_R_phoQ/1-489          ANLLLVIPLL WVAAWWSLRP IESLAKEVRE LEEHHREKLN PNTTRELTRL
402006-2-12_00288_R_phoQ/1-489        ANLLLVIPLL WVAAWWSLRP IESLAKEVRE LEEHHREKLN PNTTRELTRL
16003084_01265_R_phoQ/1-489           ANLLLVIPLL WVAAWWSLRP IESLAKEVRE LEEHHREKLN PNTTRELTRL

                                      251
113179-17_04092_S_phoQ/1-489          VSNLNRLVRS ERERYDKYRT TLTDLTHSLK TPLAVMQSTL RSLRGEKISV
16020166_04233_S_phoQ/1-489           VSNLNRLVRS ERERYDKYRT TLTDLTHSLK TPLAVMQSTL RSLRGEKISV
19221887_02545_S_phoQ/1-489           VSNLNRLVRS ERERYDKYRT TLTDLTHSLK TPLAVMQSTL RSLRGEKISV
18111299_04292_S_phoQ/1-489           VSNLNRLVRS ERERYDKYRT TLTDLTHSLK TPLAVMQSTL RSLRGEKISV
809097-16_05020_S_phoQ/1-489          VSNLNRLVRS ERERYDKYRT TLTDLTHSLK TPLAVMQSTL RSLRGEKISV
808922-16_03828_S_phoQ/1-489          VSNLNRLVRS ERERYDKYRT TLTDLTHSLK TPLAVMQSTL RSLRGEKISV
13892823_00476_S_phoQ/1-489           VSNLNRLVRS ERERYDKYRT TLTDLTHSLK TPLAVMQSTL RSLRGEKISV
14414149_01062_S_phoQ/1-489           VSNLNRLVRS ERERYDKYRT TLTDLTHSLK TPLAVMQSTL RSLRGEKISV
809156-16_05046_R_phoQ/1-489          VSNLNRLVRS ERERYDKYRT TLTDLTHSLK TPLAVMQSTL RSLRGEKISV
26048671-KSLU_03005_R_phoQ/1-489      VSNLNRLVRS ERERYDKYRT TLTDLTHSLK TPLAVMQSTL RSLRGEKISV
19852760_02287_R_phoQ/1-489           VSNLNRLVRS ERERYDKYRT TLTDLTHSLK TPLAVMQSTL RSLRGEKISV
401433-14_00644_R_phoQ/1-489          VSNLNRLVRS ERERYDKYRT TLTDLTHSLK TPLAVMQSTL RSLRGEKISV
18701876_00837_R_phoQ/1-489           VSNLNRLVRS ERERYDKYRT TLTDLTHSLK TPLAVMQSTL RSLRGEKISV
808927-16_04544_R_phoQ/1-489          VSNLNRLVRS ERERYDKYRT TLTDLTHSLK TPLAVMQSTL RSLRGEKISV
800138-16_04646_R_phoQ/1-489          VSNLNRLVRS ERERYDKYRT TLTDLTHSLK TPLAVMQSTL RSLRGEKISV
802208-17_04556_R_phoQ/1-489          VSNLNRLVRS ERERYDKYRT TLTDLTHSLK TPLAVMQSTL RSLRGEKISV
20038016-KSLU_01414_R_phoQ/1-489      VSNLNRLVRS ERERYDKYRT TLTDLTHSLK TPLAVMQSTL RSLRGEKISV
404507-16_02522_R_phoQ/1-489          VSNLNRLVRS ERERYDKYRT TLTDLTHSLK TPLAVMQNTL RSLRGEKISV
402006-2-12_00288_R_phoQ/1-489        VSNLNRLVRS ERERYDKYRT TLTDLTHSLK TPLAVMQSTL RSLRGEKISV
16003084_01265_R_phoQ/1-489           VSNLNRLVRS ERERYDKYRT TLTDLTHSLK TPLAVMQSTL RSLRGEKISV
```

301

113179-17_04092_S_phoQ/1-489	DEAEPVMLEQ	ISRISQQIGY	YLHRASMRSG	GTLLSRELHP	IAPLLDSLTS
16020166_04233_S_phoQ/1-489	DEAEPVMLEQ	ISRISQQIGY	YLHRASMRSG	GTLLSRELHP	IAPLLDSLTS
19221887_02545_S_phoQ/1-489	DEAEPVMLEQ	ISRISQQIGY	YLHRASMRSG	GTLLSRELHP	IAPLLDSLTS
18111299_04292_S_phoQ/1-489	DEAEPVMLEQ	ISRISQQIGY	YLHRASMRSG	GTLLSRELHP	IAPLLDSLTS
809097-16_05020_S_phoQ/1-489	DEAEPVMLEQ	ISRISQQIGY	YLHRASMRSG	GTLLSRELHP	IAPLLDSLTS
808922-16_03828_S_phoQ/1-489	DEAEPVMLEQ	ISRISQQIGY	YLHRASMRSG	GTLLSRELHP	IAPLLDSLTS
13892823_00476_S_phoQ/1-489	DEAEPVMLEQ	ISRISQQIGY	YLHRASMRSG	GTLLSRELHP	IAPLLDSLTS
14414149_01062_S_phoQ/1-489	DEAEPVMLEQ	ISRISQQIGY	YLHRASMRSG	GTLLSRELHP	IAPLLDSLTS
809156-16_05046_R_phoQ/1-489	DEAEPVMLEQ	ISRISQQIGY	YLHRASMRSG	GTLLSRELHP	IAPLLDSLTS
26048671-KSLU_03005_R_phoQ/1-489	DEAEPVMLEQ	ISRISQQIGY	YLHRASMRSG	GTLLSRELHP	IAPLLDSLTS
19852760_02287_R_phoQ/1-489	DEAEPVMLEQ	ISRISQQIGY	YLHRASMRSG	GTLLSRELHP	IAPLLDSLTS
401433-14_00644_R_phoQ/1-489	DEAEPVMLEQ	ISRISQQIGY	YLHRASMRSG	GTLLSRELHP	IAPLLDSLTS
18701876_00837_R_phoQ/1-489	DEAEPVMLEQ	ISRISQQIGY	YLHRASMRSG	GTLLSRELHP	IAPLLDSLTS
808927-16_04544_R_phoQ/1-489	DEAEPVMLEQ	ISRISQQIGY	YLHRASMRSG	GTLLSRELHP	IAPLLDSLTS
800138-16_04646_R_phoQ/1-489	DEAEPVMLEQ	ISRISQQIGY	YLHRASMRSG	GTLLSRELHP	IAPLLDSLTS
802208-17_04556_R_phoQ/1-489	DEAEPVMLEQ	ISRISQQIGY	YLHRASMRSG	GTLLSRELHP	IAPLLDSLTS
20038016-KSLU_01414_R_phoQ/1-489	DEAEPVMLEQ	ISRISQQIGY	YLHRASMRSG	GTLLSRELHP	IAPLLDSLTS
404507-16_02522_R_phoQ/1-489	DEAEPVMLEQ	ISRISQQIGY	YLHRASMRSG	GTLLSRELHP	IAPLLDSLTS
402006-2-12_00288_R_phoQ/1-489	DEAEPVMLEQ	ISRISQQIGY	YLHRASMRSG	GTLLSRELHP	IAPLLDSLTS
16003084_01265_R_phoQ/1-489	DEAEPVMLEQ	ISRISQQIGY	YLHRASMRSG	GTLLSRELHP	IAPLLDSLTS

351

113179-17_04092_S_phoQ/1-489	ALNKVYQRKG	VNISLDISPE	ITFVGEQNDF	MEVMGNVLDN	ACKYCLEFVE
16020166_04233_S_phoQ/1-489	ALNKVYQRKG	VNISLDISPE	ITFVGEQNDF	MEVMGNVLDN	ACKYCLEFVE
19221887_02545_S_phoQ/1-489	ALNKVYQRKG	VNISLDISPE	ISFVGEQNDF	MEVMGNVLDN	ACKYCLEFVE
18111299_04292_S_phoQ/1-489	ALNKVYQRKG	VNISLDISPE	ITFVGEQNDF	MEVMGNVLDN	ACKYCLEFVE
809097-16_05020_S_phoQ/1-489	ALNKVYQRKG	VNISLDISPE	ITFVGEQNDF	MEVMGNVLDN	ACKYCLEFVE
808922-16_03828_S_phoQ/1-489	ALNKVYQRKG	VNISLDISPE	ITFVGEQNDF	MEVMGNVLDN	ACKYCLEFVE
13892823_00476_S_phoQ/1-489	ALNKVYQRKG	VNISLDISPE	ITFVGEQNDF	MEVMGNVLDN	ACKYCLEFVE
14414149_01062_S_phoQ/1-489	ALNKVYQRKG	VNISLDISPE	ITFVGEQNDF	MEVMGNVLDN	ACKYCLEFVE
809156-16_05046_R_phoQ/1-489	ALNKVYQRKG	VNISLDISPE	ITFVGEQNDF	MEVMGNVLDN	ACKYCLEFVE
26048671-KSLU_03005_R_phoQ/1-489	ALNKVYQRKG	VNISLDISPE	ITFVGEQNDF	MEVMGNVLDN	ACKYCLEFVE
19852760_02287_R_phoQ/1-489	ALNKVYQRKG	VNISLDISPE	ITFVGEQNDF	MEVMGNVLDN	ACKYCLEFVE
401433-14_00644_R_phoQ/1-489	ALNKVYQRKG	VNISLDISPE	ITFVGEQNDF	MEVMGNVLDN	ACKYCLEFVE
18701876_00837_R_phoQ/1-489	ALNKVYQRKG	VNISLDISPE	ITFVGEQNDF	MEVMGNVLDN	ACKYCLEFVE
808927-16_04544_R_phoQ/1-489	ALNKVYQRKG	VNISLDISPE	ITFVGEQNDF	MEVMGNVLDN	ACKYCLEFVE
800138-16_04646_R_phoQ/1-489	ALNKVYQRKG	VNISLDISPE	ITFVGEQNDF	MEVMGNVLDN	ACKYCLEFVE
802208-17_04556_R_phoQ/1-489	ALNKVYQRKG	VNISLDISPE	ITFVGEQNDF	MEVMGNVLDN	ACKYCLEFVE
20038016-KSLU_01414_R_phoQ/1-489	ALNKVYQRKG	VNISLDISPE	ITFVGEQNDF	MEVMGNVLDN	ACKYCLEFVE
404507-16_02522_R_phoQ/1-489	ALNKVYQRKG	VNISLDISPE	ITFVGEQNDF	MEVMGNVLDN	ACKYCLEFVE
402006-2-12_00288_R_phoQ/1-489	ALNKVYQRKG	VNISLDISPE	ITFVGEQNDF	MEVMGNVLDN	ACKYCLEFVE
16003084_01265_R_phoQ/1-489	ALNKVYQRKG	VNISLDISPE	ITFVGEQNDF	MEVMGNVLDN	ACKYCLEFVE

401

113179-17_04092_S_phoQ/1-489	VSVRQTTDSH	LHILVEDDGP	GIPQSQRRAV	FDRGQRADTL	RPGQGVGLSV
16020166_04233_S_phoQ/1-489	VSVRQTTDSH	LHILVEDDGP	GIPQSQRRAV	FDRGQRADTL	RPGQGVGLSV
19221887_02545_S_phoQ/1-489	VSVRQTTDSH	LHILVEDDGP	GIPPSQRRAV	FDRGQRADTL	RPGQGVGLSV
18111299_04292_S_phoQ/1-489	VSVRQTTDSH	LHILVEDDGP	GIPQSQRRAV	FDRGQRADTL	RPGQGVGLSV
809097-16_05020_S_phoQ/1-489	VSVRQTTDSH	LHILVEDDGP	GIPQSQRRAV	FDRGQRADTL	RPGQGVGLSV
808922-16_03828_S_phoQ/1-489	VSVRQTTDSH	LHILVEDDGP	GIPQSQRRAV	FDRGQRADTL	RPGQGVGLSV
13892823_00476_S_phoQ/1-489	VSVRQTTDSH	LHILVEDDGP	GIPQSQRRAV	FDRGQRADTL	RPGQGVGLSV
14414149_01062_S_phoQ/1-489	VSVRQTTDSH	LHILVEDDGP	GIPQSQRRAV	FDRGQRADTL	RPGQGVGLSV
809156-16_05046_R_phoQ/1-489	VSVRQTTDSH	LHILVEDDGP	GIPQSQRRAV	FDRGQRADTL	RPGQGVGLSV
26048671-KSLU_03005_R_phoQ/1-489	VSVRQTTDSH	LHILVEDDGP	GIPQSQRRAV	FDRGQRADTL	RPGQGVGLSV
19852760_02287_R_phoQ/1-489	VSVRQTTDSH	LHILVEDDGP	GIPQSQRRAV	FDRGQRADTL	RPGQGVGLSV
401433-14_00644_R_phoQ/1-489	VSVRQTTDSH	LHILVEDDGP	GIPQSQRRAV	FDRGQRADTL	RPGQGVGLSV
18701876_00837_R_phoQ/1-489	VSVRQTTDSH	LHILVEDDGP	GIPQSQRRAV	FDRGQRADTL	RPGQGVGLSV
808927-16_04544_R_phoQ/1-489	VSVRQTTDSH	LHILVEDDGP	GIPQSQRRAV	FDRGQRADTL	RPGQGVGLSV
800138-16_04646_R_phoQ/1-489	VSVRQTTDSH	LHILVEDDGP	GIPQSQRRAV	FDRGQRADTL	RPGQGVGLSV
802208-17_04556_R_phoQ/1-489	VSVRQTTDSH	LHILVEDDGP	GIPQSQRRAV	FDRGQRADTL	RPGQGVGLSV
20038016-KSLU_01414_R_phoQ/1-489	VSVRQTTDSH	LHILVEDDGP	GIPQSQRRAV	FDRGQRADTL	RPGQGVGLSV
404507-16_02522_R_phoQ/1-489	VSVRQTTDSH	LHILVEDDGP	GIPQSQRRAV	FDRGQRADTL	RPGQGVGLSV
402006-2-12_00288_R_phoQ/1-489	VSVRQTTDSH	LHILVEDDGP	GIPQSQRRAV	FDRGQRADTL	RPGQGVGLSV
16003084_01265_R_phoQ/1-489	VSVRQTTDSH	LHILVEDDGP	GIPQSQRRAV	FDRGQRADTL	RPGQGVGLSV

	451			
113179-17_04092_S_phoQ/1-489	AREIVEQYDG	EIIAGESLLG	GACMEVVFGR	QQMEDKQS*
16020166_04233_S_phoQ/1-489	AREIVEQYDG	EIIAGESLLG	GACMEVVFGR	QQMEDKQS*
19221887_02545_S_phoQ/1-489	AREIVEQYDG	EIIAGESLLG	GACMEVVFGR	QLMEDKES*
18111299_04292_S_phoQ/1-489	AREIVEQYDG	EIIAGESLLG	GACMEVVFGR	QQMEDKQS*
809097-16_05020_S_phoQ/1-489	AREIVEQYDG	EIIAGESLLG	GACMEVVFGR	QQMEDKQS*
808922-16_03828_S_phoQ/1-489	AREIVEQYDG	EIIAGESLLG	GACMEVVFGR	QQMEDKQS*
13892823_00476_S_phoQ/1-489	AREIVEQYDG	EIIAGESLLG	GACMEVVFGR	QQMEDKQS*
14414149_01062_S_phoQ/1-489	AREIVEQYDG	EIIAGESLLG	GACMEVVFGR	QQMEDKQS*
809156-16_05046_R_phoQ/1-489	AREIVEQYDG	EIIAGESLLG	GACMEVVFGR	QQMEDKQS*
26048671-KSLU_03005_R_phoQ/1-489	AREIVEQYDG	EIIAGESLLG	GACMEVVFGR	QQMEDKQS*
19852760_02287_R_phoQ/1-489	AREIVEQYDG	EIIAGESLLG	GACMEVVFGR	QQMEDKQS*
401433-14_00644_R_phoQ/1-489	AREIVEQYDG	EIIAGESLLG	GACMEVVFGR	QQMEDKQS*
18701876_00837_R_phoQ/1-489	AREIVEQYDG	EIIAGESLLG	GACMEVVFGR	QQMEDKQS*
808927-16_04544_R_phoQ/1-489	AREIVEQYDG	EIIAGESLLG	GACMEVVFGR	QQMEDKQS*
800138-16_04646_R_phoQ/1-489	AREIVEQYDG	EIIAGESLLG	GACMEVVFGR	QQMEDKQS*
802208-17_04556_R_phoQ/1-489	AREIVEQYDG	EIIAGESLLG	GACMEVVFGR	QQMEDKQS*
20038016-KSLU_01414_R_phoQ/1-489	AREIVEQYDG	EIIAGESLLG	GACMEVVFGR	QQMEDKQS*
404507-16_02522_R_phoQ/1-489	AREIVEQYDG	EIIAGESLLG	GACMEVVFGR	QQMEDKQS*
402006-2-12_00288_R_phoQ/1-489	AREIVEQYDG	EIIAGESLLG	GACMEVVFGR	QQMEDKQS*
16003084_01265_R_phoQ/1-489	AREIVEQYDG	EIIAGESLLG	GACMEVVFGR	QQMEDKQS*

```
Alignment: D:\Documentos BASILEA\AMR LAB\Colistin resistance project\Genes\K. pneumoniae\basR (
Seaview [blocks=10 fontsize=10 A4] on Wed Jun 03 19:24:56 2020

                                   1
113179-17_02780_S_pmrA/1-224       MKILVIEDDA LLLQGLILAM QSEGYVCDGV STAHEAALSL ASNHYSLIVL
16020166_02425_S_pmrA/1-224        MKILVIEDDA LLLQGLILAM QSEGYVCDGV STAHEAALSL ASNHYSLIVL
808922-16_04524_S_pmrA/1-224       MKILVIEDDA LLLQGLILAM QSEGYVCDGV STAHEAALSL ASNHYSLIVL
809097-16_03283_S_pmrA/1-224       MKILVIEDDA LLLQGLILAM QSEGYVCDGV STAHEAALSL ASNHYSLIVL
13892823_03069_S_pmrA/1-224        MKILVIEDDA LLLQGLILAM QSEGYVCDGV STAHEAALSL ASNHYSLIVL
14414149_00076_S_pmrA/1-224        MKILVIEDDA LLLQGLILAM QSEGYVCDGV STAHEAALSL ASNHYSLIVL
18111299_00549_S_pmrA/1-224        MKILVIEDDA LLLQGLILAM QSEGYVCDGV STAHEAALSL ASNHYSLIVL
19221887_04213_S_pmrA/1-224        MKILVIEDDA LLLQGLILAM QSEGYVCDGV STAHEAALSL ASNHYSLIVL
809156-16_00535_R_pmrA/1-224       MKILVIEDDA LLLQGLILAM QSEGYVCDGV STAHEAALSL ASNHYSLIVL
404507-16_02723_R_pmrA/1-224       MKILVIEDDA LLLQGLILAM QSEGYVCDGV STAHEAALSL TSNHYSLIVL
800138-16_01732_R_pmrA/1-224       MKILVIEDDA LLLQGLILAM QSEGYVCDGV STAHEAALSL ASNHYSLIVL
802208-17_03227_R_pmrA/1-224       MKILVIEDDA LLLQGLILAM QSEGYVCDGV STAHEAALSL ASNHYSLIVL
808927-16_01792_R_pmrA/1-224       MKILVIEDDA LLLQGLILAM QSEGYVCDGV STAHEAALSL ASNHYSLIVL
16003084_04038_R_pmrA/1-224        MKILVIEDDA LLLQGLILAM QSEGYVCDGV STAHEAALSL ASNHYSLIVL
18701876_02743_R_pmrA/1-224        MKILVIEDDA LLLQGLILAM QSEGYVCDGV STAHEAALSL ASNHYSLIVL
19852760_00688_R_pmrA/1-224        MKILVIEDDA LLLQGLILAM QSEGYVCDGV STAHEAALSL ASNHYSLIVL
20038016-KSLU_00615_R_pmrA/1-224   MKILVIEDDA LLLQGLILAM QSEGYVCDGV STAHEAALSL ASNHYSLIVL
26048671-KSLU_03263_R_pmrA/1-224   MKILVIEDDA LLLQGLILAM QSEGYVCDGV STAHEAALSL ASNHYSLIVL
401433-14_00076_R_pmrA/1-224       MKILVIEDDA LLLQGLILAM QSEGYVCDGV STAHEAALSL ASNHYSLIVL
402006-2-12_00565_R_pmrA/1-224     MKILVIEDDA LLLQGLILAM QSEGYVCDGV STAHEAALSL ASNHYSLIVL

                                  51
113179-17_02780_S_pmrA/1-224       DLGLPDEDGL HFLSRMRREK MTQPVLILTA RDTLEDRISG LDTGADDYLV
16020166_02425_S_pmrA/1-224        DLGLPDEDGL HFLSRMRREK MTQPVLILTA RDTLEDRISG LDTGADDYLV
808922-16_04524_S_pmrA/1-224       DLGLPDEDGL HFLSRMRREK MTQPVLILTA RDTLEDRISG LDTGADDYLV
809097-16_03283_S_pmrA/1-224       DLGLPDEDGL HFLSRMRREK MTQPVLILTA RDTLEDRISG LDTGADDYLV
13892823_03069_S_pmrA/1-224        DLGLPDEDGL HFLSRMRREK MTQPVLILTA RDTLEDRISG LDTGADDYLV
14414149_00076_S_pmrA/1-224        DLGLPDEDGL HFLSRMRREK MTQPVLILTA RDTLEDRISG LDTGADDYLV
18111299_00549_S_pmrA/1-224        DLGLPDEDGL HFLSRMRREK MTQPVLILTA RDTLEDRISG LDTGADDYLV
19221887_04213_S_pmrA/1-224        DLGLPDEDGL HFLARMRREK MTQPVLILTA RDTLEDRISG LDTGADDYLV
809156-16_00535_R_pmrA/1-224       DLGLPDEDGL HFLSRMRREK MTQPVLILTA RDTLEDRISG LDTGADDYLV
404507-16_02723_R_pmrA/1-224       DLGLPDEDGL HFLSRMRREK MTQPVLILTA RDTLEDRISG LDTGADDYLV
800138-16_01732_R_pmrA/1-224       DLGLPDEDGL HFLSRMRREK MTQPVLILTA RDTLEDRISG LDTGADDYLV
802208-17_03227_R_pmrA/1-224       DLGLPDEDGL HFLSRMRREK MTQPVLILTA RDTLEDRISG LDTGADDYLV
808927-16_01792_R_pmrA/1-224       DLGLPDEDGL HFLSRMRREK MTQPVLILTA RDTLEDRISG LDTGADDYLV
16003084_04038_R_pmrA/1-224        DLGLPDEDGL HFLSRMRREK MTQPVLILTA RDTLEDRISG LDTGADDYLV
18701876_02743_R_pmrA/1-224        DLGLPDEDGL HFLSRMRREK MTQPVLILTA RDTLEDRISG LDTGADDYLV
19852760_00688_R_pmrA/1-224        DLGLPDEDGL HFLSRMRREK MTQPVLILTA RDTLEDRISG LDTGADDYLV
20038016-KSLU_00615_R_pmrA/1-224   DLGLPDEDGL HFLSRMRREK MTQPVLILTA RDTLEDRISG LDTGADDYLV
26048671-KSLU_03263_R_pmrA/1-224   DLGLPDEDGL HFLSRMRREK MTQPVLILTA RDTLEDRISG LDTGADDYLV
401433-14_00076_R_pmrA/1-224       DLGLPDEDGL HFLSRMRREK MTQPVLILTA RDTLEDRISG LDTGADDYLV
402006-2-12_00565_R_pmrA/1-224     DLGLPDEDGL HFLSRMRREK MTQPVLILTA RDTLEDRISG LDTGADDYLV

                                 101
113179-17_02780_S_pmrA/1-224       KPFALEELNA RIRALLRRHN NQGDNEISVG NLRLNVTRRL VCLGETALDL
16020166_02425_S_pmrA/1-224        KPFALEELNA RIRALLRRHN NQGDNEISVG NLRLNVTRRL VWLGETALDL
808922-16_04524_S_pmrA/1-224       KPFALEELNA RIRALLRRHN NQGDNEISVG NLRLNVTRRL VWLGETALDL
809097-16_03283_S_pmrA/1-224       KPFALEELNA RIRALLRRHN NQGDNEISVG NLRLNVTRRL VWLGETALDL
13892823_03069_S_pmrA/1-224        KPFALEELNA RIRALLRRHN NQGDNEISVG NLRLNVTRRL VWLGETALDL
14414149_00076_S_pmrA/1-224        KPFALEELNA RIRALLRRHN NQGDNEISVG NLRLNVTRRL VWLGETALDL
18111299_00549_S_pmrA/1-224        KPFALEELNA RIRALLRRHN NQGDNEISVG NLRLNVTRRL VWLGETALDL
19221887_04213_S_pmrA/1-224        KPFALEELNA RIRALLRRHN NQGDNEISVG DLRLNVTRRQ VWLGETALDL
809156-16_00535_R_pmrA/1-224       KPFALEELNA RIRALLRRHN NQGDNEISVG NLRLNVTRRL VWLGETALEL
404507-16_02723_R_pmrA/1-224       KPFALEELNA RIRALLRRHN NQGDNEISVG NLRLNVTRRL VWLGETALDL
800138-16_01732_R_pmrA/1-224       KPFALEELNA RIRALLRRHN NQGDNEISVG NLRLNVTRRL VWLGETALDL
802208-17_03227_R_pmrA/1-224       KPFALEELNA RIRALLRRHN NQGDNEISVG NLRLNVTRRL VWLGETALEL
808927-16_01792_R_pmrA/1-224       KPFALEELNA RIRALLRRHN NQGDNEISVG NLRLNVTRRL VWLGETALDL
16003084_04038_R_pmrA/1-224        KPFALEELNA RIRALLRRHN NQGDNEISVG NLRLNVTRRL VWLGETALDL
18701876_02743_R_pmrA/1-224        KPFALEELNA RIRALLRRHN NQGDNEISVG NLRLNVTRRL VWLGETALDL
19852760_00688_R_pmrA/1-224        KPFALEELNA RIRALLRRHN NQGDNEISVG NLRLNVTRRL VWLGETALDL
20038016-KSLU_00615_R_pmrA/1-224   KPFALEELNA RIRALLRRHN NQGDNEISVG NLRLNVTRRL VWLGETALDL
26048671-KSLU_03263_R_pmrA/1-224   KPFALEELNA RIRALLRRHN NQGDNEISVG NLRLNVTRRL VWLGETALDL
401433-14_00076_R_pmrA/1-224       KPFALEELNA RIRALLRRHN NQGDNEISVG NLRLNVTRRL VWLGETALDL
402006-2-12_00565_R_pmrA/1-224     KPFALEELNA RIRALLRRHN NQGDNEISVG NLRLNVTRRL VWLGETALDL
```

```
                                    151
113179-17_02780_S_pmrA/1-224          TPKEYALLSR LMMKAGSPVH REILYNDIYS WDNEPATNTL EVHIHNLREK
16020166_02425_S_pmrA/1-224           TPKEYALLSR LMMKAGSPVH REILYNDIYS WDNEPATNTL EVHIHNLREK
808922-16_04524_S_pmrA/1-224          TPKEYALLSR LMMKAGSPVH REILYNDIYS WDNEPATNTL EVHIHNLREK
809097-16_03283_S_pmrA/1-224          TPKEYALLSR LMMKAGSPVH REILYNDIYS WDNEPATNTL EVHIHNLREK
13892823_03069_S_pmrA/1-224           TPKEYALLSR LMMKAGSPVH REILYNDIYS WDNEPATNTL EVHIHNLREK
14414149_00076_S_pmrA/1-224           TPKEYALLSR LMMKAGSPVH REILYNDIYS WDNEPATNTL EVHIHNLREK
18111299_00549_S_pmrA/1-224           TPKEYALLSR LMMKAGSPVH REILYNDIYS WDNEPATNTL EVHIHNLREK
19221887_04213_S_pmrA/1-224           TPKEYALLSR LMMKAGSPVH REILYNDIYS WDNEPATNTL EVHIHNLRDK
809156-16_00535_R_pmrA/1-224          TPKEYALLSR LMMKAGSPVH REILYNDIYS WDNEPATNTL EVHIHNLREK
404507-16_02723_R_pmrA/1-224          TPKEYALLSR LMMKAGSPVH REILYNDIYS WDNEPATNTL EVHIHNLREK
800138-16_01732_R_pmrA/1-224          TPKEYALLSR LMMKAGSPVH REILYNDIYS WDNEPATNTL EVHIHNLREK
802208-17_03227_R_pmrA/1-224          TPKEYALLSR LMMKAGSPVH REILYNDIYS WDNEPATNTL EVHIHNLREK
808927-16_01792_R_pmrA/1-224          TPKEYALLSR LMMKAGSPVH REILYNDIYS WDNEPATNTL EVHIHNLREK
16003084_04038_R_pmrA/1-224           TPKEYALLSR LMMKAGSPVH REILYNDIYS WDNEPATNTL EVHIHNLREK
18701876_02743_R_pmrA/1-224           TPKEYALLSR LMMKAGSPVH REILYNDIYS WDNEPATNTL EVHIHNLREK
19852760_00688_R_pmrA/1-224           TPKEYALLSR LMMKAGSPVH REILYNDIYS WDNEPATNTL EVHIHNLREK
20038016-KSLU_00615_R_pmrA/1-224      TPKEYALLSR LMMKAGSPVH REILYNDIYS WDNEPATNTL EVHIHNLREK
26048671-KSLU_03263_R_pmrA/1-224      TPKEYALLSR LMMKAGSPVH REILYNDIYS WDNEPATNTL EVHIHNLREK
401433-14_00076_R_pmrA/1-224          TPKEYALLSR LMMKAGSPVH REILYNDIYS WDNEPATNTL EVHIHNLREK
402006-2-12_00565_R_pmrA/1-224        TPKEYALLSR LMMKAGSPVH REILYNDIYS WDNEPATNTL EVHIHNLREK

                                    201
113179-17_02780_S_pmrA/1-224          IGKSRIRTVR GFGYMLANNI DTE*
16020166_02425_S_pmrA/1-224           IGKSRIRTVR GFGYMLANNI DTE*
808922-16_04524_S_pmrA/1-224          IGKSRIRTVR GFGYMLANNI DTE*
809097-16_03283_S_pmrA/1-224          IGKSRIRTVR GFGYMLANNI DTE*
13892823_03069_S_pmrA/1-224           IGKSRIRTVR GFGYMLANNI DTE*
14414149_00076_S_pmrA/1-224           IGKSRIRTVR GFGYMLANNI DTE*
18111299_00549_S_pmrA/1-224           IGKSRIRTVR GFGYMLANNI DTE*
19221887_04213_S_pmrA/1-224           IGKSRIRTVR GFGYMLANHN ETE*
809156-16_00535_R_pmrA/1-224          IGKSRIRTVR GFGYMLANNI DTE*
404507-16_02723_R_pmrA/1-224          IGKSRIRTVR GFGYMLANNI DTE*
800138-16_01732_R_pmrA/1-224          IGKSRIRTVR GFGYMLANNI DTE*
802208-17_03227_R_pmrA/1-224          IGKSRIRTVR GFGYMLANNI DTE*
808927-16_01792_R_pmrA/1-224          IGKSRIRTVR GFGYMLANNI DTE*
16003084_04038_R_pmrA/1-224           IGKSRIRTVR GFGYMLVNNI DTE*
18701876_02743_R_pmrA/1-224           IGKSRIRTVR GFGYMLANNI DTE*
19852760_00688_R_pmrA/1-224           IGKSRIRTVR GFGYMLVNNI DTE*
20038016-KSLU_00615_R_pmrA/1-224      IGKSRIRTVR GFGYMLANNI DTE*
26048671-KSLU_03263_R_pmrA/1-224      IGKSRIRTVR GFGYMLANNI DTE*
401433-14_00076_R_pmrA/1-224          IGKSRIRTVR GFGYMLANNI DTE*
402006-2-12_00565_R_pmrA/1-224        IGKSRIRTVR GFGYMLANNI DTE*
```

```
Alignment: D:\Documentos BASILEA\AMR LAB\Colistin resistance project\Genes\K. pneumoniae\basS(p
Seaview [blocks=10 fontsize=10 A4] on Wed Jun 03 19:17:36 2020

                                  1
113179-17_02781_S_pmrB/1-366      MALFATETWT MRHRLLLTIG AILVVCQLIS VFWLWHESKE QIQLLVASAI
16020166_02424_S_pmrB/1-366       MALFATETWT MRHRLLLTIG AILVVCQLIS VFWLWHESKE QIQLLVASAI
809097-16_03282_S_pmrB/1-366      MALFATETWT MRHRLLLTIG AILVVCQLIS VFWLWHESKE QIQLLVASAI
808922-16_04525_S_pmrB/1-366      MALFATETWT MRHRLLLTIG AILVVCQLIS VFWLWHESKE QIQLLVASAI
13892823_03070_S_pmrB/1-366       MALFATETWT MRHRLLLTIG AILVVCQLIS VFWLWHESKE QIQLLVASAI
14414149_00077_S_pmrB/1-366       MALFATETWT MRHRLLLTIG AILVVCQLIS VFWLWHESKE QIQLLVASAI
18111299_00548_S_pmrB/1-366       MALFATETWT MRHRLLLTIG AILVVCQLIS VFWLWHESKE QIQLLVASAI
19221887_04212_S_pmrB/1-366       MALFATENWT MRHRLLLTIG AILVVCQLIS VFWLWHESKE QIQLLVASAI
809156-16_00534_R_pmrB/1-366      MALFATETWT MRHRLLLTIG AILVVCQLIS VFWLWHESKE QIQLLVASAI
26048671-KSLU_03262_R_pmrB/1-366  MALFATETWT MRHRLLLTIG AILVVCQLIS VFWLWHESKE QIQLLVASAI
401433-14_00077_R_pmrB/1-366      MALFATETWT MRHRLLLTIG AILVVCQLIS VFWLWHESKE QIQLLVASAI
402006-2-12_00564_R_pmrB/1-366    MALFATETWT MRHRLLLTIG AILVVCQLIS VFWLWHESKE QIQLLVASAI
404507-16_02724_R_pmrB/1-366      MALFATETWT MRHRLLLTIG AILVVCQLIS VFWLWHESKE QIQLLVASAI
800138-16_01731_R_pmrB/1-366      MALFATETWT MRHRLLLTIG AILVVCQLIS VFWLWHESKE QIQLLVASAI
802208-17_03228_R_pmrB/1-366      MALFATETWT MRHRLLLTIG AILVVCQLIS VFWLWHESKE QIQLLVASAI
18701876_02742_R_pmrB/1-366       MALFATETWT MRHRLLLTIG AILVVCQLIS VFWLWHESKE QIQLLVASAI
808927-16_01791_R_pmrB/1-366      MALFATETWT MRHRLLLTIG AILVVCQLIS VFWLWHESKE QIQLLVASAI
16003084_04039_R_pmrB/1-366       MALFATETWT MRHRLLLTIG AILVVCQLIS VFWLWHESKE QIQLLVASAI
20038016-KSLU_00614_R_pmrB/1-366  MALFATETWT MRHRLLLTIG AILVVCQLIS VFWLWHESKE QIQLLVASAI
19852760_00687_R_pmrB/1-366       MALFATETWT MRHRLLLTIG AILVVCQLIS VFWLWHESKE QIQLLVASAI

                                 51
113179-17_02781_S_pmrB/1-366      EGHNNQKHVE HEVREAVASL LVPSLLIVGL ALYISMLAVR KITRPLSRLQ
16020166_02424_S_pmrB/1-366       EGHNNQKHVE HEVREAVASL LVPSLLIVGL ALYISMLAVR KITRPLSRLQ
809097-16_03282_S_pmrB/1-366      EGHNNQKHVE HEVREAVASL LVPSLLIVGL ALYISMLAVR KITRPLSRLQ
808922-16_04525_S_pmrB/1-366      EGHNNQKHVE HEVREAVASL LVPSLLIVGL ALYISMLAVR KITRPLSRLQ
13892823_03070_S_pmrB/1-366       EGHNNQKHVE HEVREAVASL LVPSLLIVGL ALYISMLAVR KITRPLSRLQ
14414149_00077_S_pmrB/1-366       EGHNNQKHVE HEVREAVASL LVPSLLIVGL ALYISMLAVR KITRPLSRLQ
18111299_00548_S_pmrB/1-366       EGHNNQKHVE HEVREAVASL LVPSLLIVGL ALYISMLAVR KITRPLSRLQ
19221887_04212_S_pmrB/1-366       EGHNNQKHVE HEVREAVASL LVPSLLIVGL ALYISMLAVR KITRPLSRLQ
809156-16_00534_R_pmrB/1-366      EGHNNQKHVE HEVREAVASL LVPSLLIVGL ALYISMLAVR KITRPLSRLQ
26048671-KSLU_03262_R_pmrB/1-366  EGHNNQKHVE HEVREAVASL LVPSLLIVGL ALYISMLAVR KITRPLSRLQ
401433-14_00077_R_pmrB/1-366      EGHNNQKHVE HEVREAVASL LVPSLLIVGL ALYISMLAVR KITRPLSRLQ
402006-2-12_00564_R_pmrB/1-366    EGHNNQKHVE HEVREAVASL LVPSLLIVGL ALYISMLAVR KITRPLSRLQ
404507-16_02724_R_pmrB/1-366      EGHNNQKHVE HEVREAVASL LVPSLLIVGL ALYISMLAVR KITRPLSRLQ
800138-16_01731_R_pmrB/1-366      EGHNNQKHVE HEVREAVASL LVPSLLIVGL ALYISMLAVR KITRPLSRLQ
802208-17_03228_R_pmrB/1-366      EGHNNQKHVE HEVREAVASL LVPSLLIVGL ALYISMLAVR KITRPLSRLQ
18701876_02742_R_pmrB/1-366       EGHNNQKHVE HEVREAVASL LVPSLLIVGL ALYISMLAVR KITRPLSRLQ
808927-16_01791_R_pmrB/1-366      EGHNNQKHVE HEVREAVASL LVPSLLIVGL ALYISMLAVR KITRPLSRLQ
16003084_04039_R_pmrB/1-366       EGHNNQKHVE HEVREAVASL LVPSLLIVGL ALYISMLAVR KITRPLSRLQ
20038016-KSLU_00614_R_pmrB/1-366  EGHNNQKHVE HEVREAVASL LVPSLLIVGL ALYISMLAVR KITRPLSRLQ
19852760_00687_R_pmrB/1-366       EGHNNQKHVE HEVREAVASL LVPSLLIVGL ALYISMLAVR KITRPLSRLQ

                                101
113179-17_02781_S_pmrB/1-366      SELENRTPDN LTPIVLSESV PEVTAVTTAL NQLVSRLNLT LDRERLFTAD
16020166_02424_S_pmrB/1-366       SELENRTPDN LTPIVLSESV PEVTAVTTAL NQLVSRLNLT LDRERLFTAD
809097-16_03282_S_pmrB/1-366      SELENRTPDN LTPIVLSESV PEVTAVTTAL NQLVSRLNLT LDRERLFTAD
808922-16_04525_S_pmrB/1-366      SELENRTPDN LTPIVLSESV PEVTAVTTAL NQLVSRLNLT LDRERLFTAD
13892823_03070_S_pmrB/1-366       SELENRTPDN LTPIVLSESV PEVTAVTTAL NQLVSRLNLT LDRERLFTAD
14414149_00077_S_pmrB/1-366       SELENRTPDN LTPIVLSESV PEVTAVTTAL NQLVSRLNLT LDRERLFTAD
18111299_00548_S_pmrB/1-366       SELENRTPDN LTPIVLSESV PEVTAVTTAL NQLVSRLNLT LDRERLFTAD
19221887_04212_S_pmrB/1-366       SELESRTPDN LTPIVLSESV PEVTAVTTAL NQLVSRLNLT LDRERLFTAD
809156-16_00534_R_pmrB/1-366      SELENRTPDN LTPIVLSESV PEVTAVTTAL NQLVSRLNLT LDRERLFTAD
26048671-KSLU_03262_R_pmrB/1-366  SELENRTPDN LTPIVLSESV PEVTAVTTAL NQLVSRLNLT LDRERLFTAD
401433-14_00077_R_pmrB/1-366      SELENRTPDN LTPIVLSESV PEVTAVTTAL NQLVSRLNLT LDRERLFTAD
402006-2-12_00564_R_pmrB/1-366    SELENRTPDN LTPIVLSESV PEVTAVTTAL NQLVSRLNLT LDRERLFTAD
404507-16_02724_R_pmrB/1-366      SELENRTPDN LTPIVLSESV PEVTAVTTAL NQLVSRLNLT LDRERLFTAD
800138-16_01731_R_pmrB/1-366      SELENRTPDN LTPIVLSESV PEVTAVTTAL NQLVSRLNLT LDRERLFTAD
802208-17_03228_R_pmrB/1-366      SELENRTPDN LTPIVLSESV PEVTAVTTAL NQLVSRLNLT LDRERLFTAD
18701876_02742_R_pmrB/1-366       SELENRTPDN LTPIVLSESV PEVTAVTTAL NQLVSRLNLT LDRERLFTAD
808927-16_01791_R_pmrB/1-366      SELENRTPDN LTPIVLSESV PEVTAVTTAL NQLVSRLNLT LDRERLFTAD
16003084_04039_R_pmrB/1-366       SELENRTPDN LTPIVLSESV PEVTAVTTAL NQLVSRLNLT LDRERLFTAD
20038016-KSLU_00614_R_pmrB/1-366  SELENRTPDN LTPIVLSESV PEVTAVTTAL NQLVSRLNLT LDRERLFTAD
19852760_00687_R_pmrB/1-366       SELENRTPDN LTPIVLSESV PEVTAVTTAL NQLVSRLNLT LDRERLFTAD
```

```
                                   151
113179-17_02781_S_pmrB/1-366        VAHELRTPLA GLRLHLELLA KVHGMGVDPL IQRLDQMTTS ISQLLQLARV
16020166_02424_S_pmrB/1-366         VAHELRTPLA GLRLHLELLA KVHGMGVDPL IQRLDQMTTS ISQLLQLARV
809097-16_03282_S_pmrB/1-366        VAHELRTPLA GLRLHLELLA KVHGMGVDPL IQRLDQMTTS ISQLLQLARV
808922-16_04525_S_pmrB/1-366        VAHELRTPLA GLRLHLELLA KVHGMGVDPL IQRLDQMTTS ISQLLQLARV
13892823_03070_S_pmrB/1-366         VAHELRTPLA GLRLHLELLA KVHGMGVDPL IQRLDQMTTS ISQLLQLARV
14414149_00077_S_pmrB/1-366         VAHELRTPLA GLRLHLELLA KVHGMGVDPL IQRLDQMTTS ISQLLQLARV
18111299_00548_S_pmrB/1-366         VAHELRTPLA GLRLHLELLA KVHGMGVDPL IQRLDQMTTS ISQLLQLARV
19221887_04212_S_pmrB/1-366         VAHELRTPLA GLRLHLELLA KVHGMGVDPL IQRLDQMTTS ISQLLQLARV
809156-16_00534_R_pmrB/1-366        VAHELRTPLA GLRLHLELLA KVHGMGVDPL IQRLDQMTTS ISQLLQLARV
26048671-KSLU_03262_R_pmrB/1-366    VAHELRTPLA GLRLHLELLA KVHGMGVDPL IQRLDQMTTS ISQLLQLARV
401433-14_00077_R_pmrB/1-366        VAHELRTPLA GLRLHLELLA KVHGMGVDPL IQRLDQMTTS ISQLLQLARV
402006-2-12_00564_R_pmrB/1-366      VAHELRTPLA GLRLHLELLA KVHGMGVDPL IQRLDQMTTS ISQLLQLARV
404507-16_02724_R_pmrB/1-366        VAHELRTPLA GLRLHLELLA KVHGMGVDPL IQRLDQMTTS ISQLLQLARV
800138-16_01731_R_pmrB/1-366        VAHELRTPLA GLRLHLELLA KVHGMGVDPL IQRLDQMTTS ISQLLQLARV
802208-17_03228_R_pmrB/1-366        VAHELRTPLA GLRLHLELLA KVHGMGVDPL IQRLDQMTTS ISQLLQLARV
18701876_02742_R_pmrB/1-366         VAHELRTPLA GLRLHLELLA KVHGMGVDPL IQRLDQMTTS ISQLLQLARV
808927-16_01791_R_pmrB/1-366        VAHELRTPLA GLRLHLELLA KVHGMGVDPL IQRLDQMTTS ISQLLQLARV
16003084_04039_R_pmrB/1-366         VAHELRTPLA GLRLHLELLA KVHGMGVDPL IQRLDQMTTS ISQLLQLARV
20038016-KSLU_00614_R_pmrB/1-366    VAHELRTPLA GLRLHLELLA KVHGMGVDPL IQRLDQMTTS ISQLLQLARV
19852760_00687_R_pmrB/1-366         VAHELRTPLA GLRLHLELLA KVHGMGVDPL IQRLDQMTTS ISQLLQLARV

                                   201
113179-17_02781_S_pmrB/1-366        GQSFSAGSYQ QVLLLDDVVK PLQDELEAML AQRQQRLLLT DIENETVVSG
16020166_02424_S_pmrB/1-366         GQSFSAGSYQ QVLLLDDVVK PLQDELEAML AQRQQRLLLT DIENETVVSG
809097-16_03282_S_pmrB/1-366        GQSFSAGSYQ QVLLLDDVVK PLQDELEAML AQRQQRLLLT DIENEAVVSG
808922-16_04525_S_pmrB/1-366        GQSFSAGSYQ QVLLLDDVVK PLQDELEAML AQRQQRLLLT DIENEAVVSG
13892823_03070_S_pmrB/1-366         GQSFSAGSYQ QVLLLDDVVK PLQDELEAML AQRQQRLLLT DIENEAVVSG
14414149_00077_S_pmrB/1-366         GQSFSAGSYQ QVLLLDDVVK PLQDELEAML AQRQQRLLLT DIENEAVVSG
18111299_00548_S_pmrB/1-366         GQSFSAGSYQ QVLLLDDVVK PLQDELEAML AQRQQRLLLT DIENEAVVSG
19221887_04212_S_pmrB/1-366         GQSFSAGSYQ QVLLLDDVVK PLQDELETML AERQQRLLLT DVESEAVVSG
809156-16_00534_R_pmrB/1-366        GQSFSAGSYQ QVLLLDDVVK PLQDELEAML AQRQQRLLLT DIENEAVVSG
26048671-KSLU_03262_R_pmrB/1-366    GQSFSAGSYQ QVLLLDDVVK PLQDELEAML AQRQQRLLLT DIENETVVSG
401433-14_00077_R_pmrB/1-366        GQSFSAGSYQ QVLLLDDVVK PLQDELEAML AQRQQRLLLT DIENEAVVSG
402006-2-12_00564_R_pmrB/1-366      GQSFSAGSYQ QVLLLDDVVK PLQDELEAML AQRQQRLLLT DIENEAVVSG
404507-16_02724_R_pmrB/1-366        GQSFSAGSYQ QVMLLDDVVK PLQDELEAML AQRQQRLLLT DIENEAVVSG
800138-16_01731_R_pmrB/1-366        GQSFSAGSYQ QVLLLDDVVK PLQDELEAML AQRQQRLLLT DIENETVVSG
802208-17_03228_R_pmrB/1-366        GQSFSAGSYQ QVLLLDDVVK PLQDELEAML AQRQQRLLLT DIENEAVVSG
18701876_02742_R_pmrB/1-366         GQSFSAGSYQ QVLLLDDVVK PLQDELEAML AQRQQRLLLT DIENEAVVSG
808927-16_01791_R_pmrB/1-366        GQSFSAGSYQ QVLLLDDVVK PLQDELEAML AQRQQRLLLT DIENEAVVSG
16003084_04039_R_pmrB/1-366         GQSFSAGSYQ QVLLLDDVVK PLQDELEAML AQRQQRLLLT DIENEAVVSG
20038016-KSLU_00614_R_pmrB/1-366    GQSFSAGSYQ QVLLLDDVVK PLQDELEAML AQRQQRLLLT DIENEAVVSG
19852760_00687_R_pmrB/1-366         GQSFSAGSYQ QVLLLDDVVK PLQDELEAML AQRQQRLLLT DIENEAVVSG

                                   251
113179-17_02781_S_pmrB/1-366        DATLIRVILR NLVENAHRYS PEGSTIRVSV KAGLMPVMAV EDEGPGIDEA
16020166_02424_S_pmrB/1-366         DATLIRVILR NLVENAHRYS PEGSTIRVSV KAGLMPVMAV EDEGPGIDEA
809097-16_03282_S_pmrB/1-366        DATLIGVILR NLVENAHRYS PEGSTIRVSV KAGLMPVMAV EDEGPGIDEA
808922-16_04525_S_pmrB/1-366        DATLIRVILR NLVENAHRYS PEGSTIRVSV KAGLMPVMAV EDEGPGIDEA
13892823_03070_S_pmrB/1-366         DATLIGVILR NLVENAHRYS PEGSTIRVSV KAGLMPVMAV EDEGPGIDEA
14414149_00077_S_pmrB/1-366         DATLIGVILR NLVENAHRYS PEGSTIRVSV KAGLMPVMAV EDEGPGIDEA
18111299_00548_S_pmrB/1-366         DATLIGVILR NLVENAHRYS PEGSTIRVSV KAGLMPVMAV EDEGPGIDEA
19221887_04212_S_pmrB/1-366         DATLIRVILR NLVENAHRYS PQGSTIRVSV KAGLMPVMAV EDEGPGIDEA
809156-16_00534_R_pmrB/1-366        DATLIRVILR NLVENAHRYS PEGSTIRVSV KAGLMPVMAV EDEGPGIDEA
26048671-KSLU_03262_R_pmrB/1-366    DATLIRVILR NLVENAHRYS PEGSTIRVSV KAGLMPVMAV EDEGPGIDEA
401433-14_00077_R_pmrB/1-366        DATLIGVILR NLVENAHRYS PEGSTIRVSV KAGLMPVMAV EDEGPGIDEA
402006-2-12_00564_R_pmrB/1-366      DATLIGVILR NLVENAHRYS PEGSTIRVSV KAGLMPVMAV EDEGPGIDEA
404507-16_02724_R_pmrB/1-366        DATLIRVILR NLVENAHRYS PEGSTIRVSV KAGLMPVMAV EDEGPGIDEA
800138-16_01731_R_pmrB/1-366        DATLIRVILR NLVENAHRYS PEGSTIRVSV KAGLMPVMAV EDEGPGIDEA
802208-17_03228_R_pmrB/1-366        DATLIRVILR NLVENAHRYS PEGSTIRVSV KAGLMPVMAV EDEGPGIDEA
18701876_02742_R_pmrB/1-366         DATLIGVILR NLVENAHRYS PEGSTIRVSV KAGLMPVMAV EDEGPGIDEA
808927-16_01791_R_pmrB/1-366        DATLIRVILR NLVENAHRYS PEGSTIRVSV KAGLMPVMAV EDEGPGIDEA
16003084_04039_R_pmrB/1-366         DATLIRVILR NLVENAHRYS PEGSTIRVSV KAGLMPVMAV EDEGPGIDEA
20038016-KSLU_00614_R_pmrB/1-366    DATLIGVILR NLVENAHRYS PEGSTIRVSV KAGLMPVMAV EDEGPGIDEA
19852760_00687_R_pmrB/1-366         DATLIRVILR NLVENAHRYS PEGSTIRVSV KAGLMPVMAV EDEGPGIDEA
```

```
                                  301
113179-17_02781_S_pmrB/1-366        KSGELSKAFV RMDSRYGGIG LGLSIVTRIA QLHDAQFFLH NRQPGPGVRA
16020166_02424_S_pmrB/1-366         KSGELSKAFV RMDSRYGGIG LGLSIVTRIA QLHDAQFFLH NRQPGPGVRA
809097-16_03282_S_pmrB/1-366        KSGELSKAFV RMDSRYGGIG LGLSIVTRIA QLHDAQFFLH NRQPGPGVRA
808922-16_04525_S_pmrB/1-366        KSGELSKAFV RMDSRYGGIG LGLSIVTRIA QLHDAQFFLH NRQPGPGVRA
13892823_03070_S_pmrB/1-366         KSGELSKAFV RMDSRYGGIG LGLSIVTRIA QLHDAQFFLH NRQPGPGVRA
14414149_00077_S_pmrB/1-366         KSGELSKAFV RMDSRYGGIG LGLSIVTRIA QLHDAQFFLH NRQPGPGVRA
18111299_00548_S_pmrB/1-366         KSGELSKAFV RMDSRYGGIG LGLSIVTRIA QLHDAQFFLH NRQPGPGVRA
19221887_04212_S_pmrB/1-366         KSGELSKAFV RMDSRYGGIG LGLSIVTRIA QLHDAQFFLH NRQPGPGVRA
809156-16_00534_R_pmrB/1-366        KSGELSKAFV RMDSRYGGIG LGLSIVTRIA QLHDAQFFLH NRQPGPGVRA
26048671-KSLU_03262_R_pmrB/1-366    KSGELSKAFV RMDSRYGGIG LGLSIVTRIA QLHDAQFFLH NRQPGPGVRA
401433-14_00077_R_pmrB/1-366        KSGELSKAFV RMDSRYGGIG LGLSIVTRIA QLHDAQFFLH NRQPGPGVRA
402006-2-12_00564_R_pmrB/1-366      KSGELSKAFV RMDSRYGGIG LGLSIVTRIA QLHDAQFFLH NRQPGPGVRA
404507-16_02724_R_pmrB/1-366        KSGELSKAFV RMDSRYGGIG LGLSIVTRIA QLHDAQFFLH NRQPGPGVRA
800138-16_01731_R_pmrB/1-366        KSGELSKAFV RMDSRYGGIG LGLSIVTRIA QLHDAQFFLH NRQPGPGVRA
802208-17_03228_R_pmrB/1-366        KSGELSKAFV RMDSRYGGIG LGLSIVTRIA QLHDAQFFLH NRQPGPGVRA
18701876_02742_R_pmrB/1-366         KSGELSKAFV RMDSRYGGIG LGLSIVTRIA QLHDAQFFLH NRQPGPGVRA
808927-16_01791_R_pmrB/1-366        KSGELSKAFV RMDSRYGGIG LGLSIVTRIA QLHDAQFFLH NRQPGPGVRA
16003084_04039_R_pmrB/1-366         KSGELSKAFV RMDSRYGGIG LGLSIVTRIA QLHDAQFFLH NRQPGPGVRA
20038016-KSLU_00614_R_pmrB/1-366    KSGELSKAFV RMDSRYGGIG LGLSIVTRIA QLHDAQFFLH NRQPGPGVRA
19852760_00687_R_pmrB/1-366         KSGELSKAFV RMDSRYGGIG LGLSIVTRIA QLHDAQFFLH NRQPGPGVRA

                                  351
113179-17_02781_S_pmrB/1-366        WVLFPQRGGQ NVSTH*
16020166_02424_S_pmrB/1-366         WVLFPQRGGQ NVSTH*
809097-16_03282_S_pmrB/1-366        WVLFPQRGGQ NVSTH*
808922-16_04525_S_pmrB/1-366        WVLFPQRGGQ NVSTH*
13892823_03070_S_pmrB/1-366         WVLFPQRGGQ NVSTH*
14414149_00077_S_pmrB/1-366         WVLFPQRGGQ NVSTH*
18111299_00548_S_pmrB/1-366         WVLFPQRGGQ NVSTH*
19221887_04212_S_pmrB/1-366         WVLFPRRAGQ NVSTH*
809156-16_00534_R_pmrB/1-366        WVLFPQRGGQ NVSTH*
26048671-KSLU_03262_R_pmrB/1-366    WVLFPQRGGQ NVSTH*
401433-14_00077_R_pmrB/1-366        WVLFPQRGGQ NVSTH*
402006-2-12_00564_R_pmrB/1-366      WVLFPQRGGQ NVSTH*
404507-16_02724_R_pmrB/1-366        WVLFPQRGGQ NVSTH*
800138-16_01731_R_pmrB/1-366        WVLFPQRGGQ NVSTH*
802208-17_03228_R_pmrB/1-366        WVLFPQRGGQ NVSTH*
18701876_02742_R_pmrB/1-366         WVLFPQRGGQ NVSTH*
808927-16_01791_R_pmrB/1-366        WVLFPQRGGQ NVSTH*
16003084_04039_R_pmrB/1-366         WVLFPQRGGQ NVSTH*
20038016-KSLU_00614_R_pmrB/1-366    WVLFPQRGGQ NVSTH*
19852760_00687_R_pmrB/1-366         WVLFPQRGGQ NVSTH*
```

Alignment: D:\Documentos BASILEA\AMR LAB\Colistin resistance project\Genes\K. pneumoniae\eptA\K
Seaview [blocks=10 fontsize=10 A4] on Wed Jun 03 19:31:46 2020

```
                                   1
113179-17_02779_S_pmrC/1-547       MSLLPLRRPV VSRTTYLILF ACYIGICLNL AFYRQVFPLL PVNSLHNWLV
16020166_02426_S_pmrC/1-547        MSLLPLRRPV VSRTTYLILF ACYIGICLNL AFYRQVFPLL PVNSLHNWLV
808922-16_04523_S_pmrC/1-547       MSLLPLRRPV VSRTTYLILF ACYIGICLNL AFYRQVFPLL PVNSLHNWLV
809097-16_03284_S_pmrC/1-547       MSLLPLRRPV VSRTTYLILF ACYIGIFLNL AFYRQVFPLL PVNSLHNWLV
13892823_03068_S_pmrC/1-547        MSLLPLRRPV VSRTTYLILF ACYIGIFLNL AFYRQVFPLL PVNSLHNWLV
14414149_00075_S_pmrC/1-547        MSLLPLRRPV VSRTTYLILF ACYIGIFLNL AFYRQVFPLL PVNSLHNWLV
18111299_00550_S_pmrC/1-547        MSLLPLRRPV VSRTTYLILF ACYIGIFLNL AFYRQVFPLL PVNSLHNWLV
19221887_04214_S_pmrC/1-547        MSLLPLRRPV VSHTTYLIIF AVYIGLFLNL AFYRQAYTLL PVNNLHTALV
809156-16_00536_R_pmrC/1-547       MSLLPLRRPV VSRTTYLILF ACYIGICLNL AFYRQVFPLL PVNSLHNWLV
26048671-KSLU_03264_R_pmrC/1-547   MSLLPLRRPV VSRTTYLILF ACYIGICLNL AFYRQVFPLL PVNSLHNWLV
401433-14_00075_R_pmrC/1-547       MSLLPLRRPV VSRTTYLILF ACYIGIFLNL AFYRQVFPLL PVNSLHNWLV
20038016-KSLU_00616_R_pmrC/1-547   MSLLPLRRPV VSRTTYLILF ACYIGIFLNL AFYRQVFPLL PVNSLHNWLV
402006-2-12_00566_R_pmrC/1-547     MSLLPLRRPV VSRTTYLILF ACYIGIFLNL AFYRQVFPLL PVNSLHNWLV
404507-16_02722_R_pmrC/1-547       MSLLPLRRPV VSRTTYLILF ACYIGICLNL AFYRQVFPLL PVNSLHNWLV
808927-16_01793_R_pmrC/1-547       MSLLPLRRPV VSRTTYLILF ACYIGICLNL AFYRQVFPLL PVNSLHNWLV
19852760_00689_R_pmrC/1-547        MSLLPLRRPV VSRTTYLILF ACYISICLNL AFYRQVFPLL PVNSLHNWLV
16003084_04037_R_pmrC/1-547        MSLLPLRRPV VSRTTYLILF ACYISICLNL AFYRQVFPLL PVNSLHNWLV
800138-16_01733_R_pmrC/1-547       MSLLPLRRPV VSRTTYLILF ACYIGICLNL AFYRQVFPLL PVNSLHNWLV
18701876_02744_R_pmrC/1-547        MSLLPLRRPV VSRTTYLILF ACYIGIFLNL AFYRQVFPLL PVNSLHNWLV
802208-17_03226_R_pmrC/1-547       MSLLPLRRPV VSRTTYLILF ACYIGICLNL AFYRQVFPLL PVNSLHNWLV

                                  51
113179-17_02779_S_pmrC/1-547       FLSMPIVAIS VMNILTTLAS FLKLDRLVIS LFILLSASAQ YFIWNFGVVI
16020166_02426_S_pmrC/1-547        FLSMPIVAIS VMNILTTLAS FLKLDRLVIS LFILLSASAQ YFIWNFGVVI
808922-16_04523_S_pmrC/1-547       FLSMPIVAIS VMNILTTLAS FLKLDRLVIS LFILLSASAQ YFIWNFGVVI
809097-16_03284_S_pmrC/1-547       FLSMPIVAIS VMNILTTLAS FLKLDRLVIS LFILLSASAQ YFIWNFGVVI
13892823_03068_S_pmrC/1-547        FLSMPIVAIS VMNILTTLAS FLKLDRLVIS LFILLSASAQ YFIWNFGVVI
14414149_00075_S_pmrC/1-547        FLSMPIVAIS VMNILTTLAS FLKLDRLVIS LFILLSASAQ YFIWNFGVVI
18111299_00550_S_pmrC/1-547        FLSMPIVAIS VMNILTTLAS FLKLDRLVIS LFILLSASAQ YFIWNFGVVI
19221887_04214_S_pmrC/1-547        FLSMPLVAFS VMNILTTLAS FLKLDRLVIS LFILLSASAQ YFIWSFGVVI
809156-16_00536_R_pmrC/1-547       FLSMPIVAIS VMNILTTLAS FLKLDRLVIS LFILLSASAQ YFIWNFGVVI
26048671-KSLU_03264_R_pmrC/1-547   FLSMPIVAIS VMNILTTLAS FLKLDRLVIS LFILLSASAQ YFIWNFGVVI
401433-14_00075_R_pmrC/1-547       FLSMPIVAIS VMNILTTLAS FLKLDRLVIS LFILLSASAQ YFIWNFGVVI
20038016-KSLU_00616_R_pmrC/1-547   FLSMPIVAIS VMNILTTLAS FLKLDRLVIS LFILLSASAQ YFIWNFGVVI
402006-2-12_00566_R_pmrC/1-547     FLSMPIVAIS VMNILTTLAS FLKLDRLVIS LFILLSASAQ YFIWNFGVVI
404507-16_02722_R_pmrC/1-547       FLSMPIVAIS VMNILTTLAS FLKLDRLVIS LFILLSASAQ YFIWNFGVVI
808927-16_01793_R_pmrC/1-547       FLSMPIVAIS VMNILTTLAS FLKLDRLVIS LFILLSASAQ YFIWNFGVVI
19852760_00689_R_pmrC/1-547        FLSMPIVAIS VMNILTTLAS FLKLDRLVIS LFILLSASAQ YFIWNFGVVI
16003084_04037_R_pmrC/1-547        FLSMPIVAIS VMNILTTLAS FLKLDRLVIS LFILLSASAQ YFIWNFGVVI
800138-16_01733_R_pmrC/1-547       FLSMPIVAIS VMNILTTLAS FLKLDRLVIS LFILLSASAQ YFIWNFGVVI
18701876_02744_R_pmrC/1-547        FLSMPIVAIS VMNILTTLAS FLKLDRLVIS LFILLSASAQ YFIWNFGVVI
802208-17_03226_R_pmrC/1-547       FLSMPIVAIS VMNILTTLAS FLKLDRLVIS LFILLSASAQ YFIWNFGVVI

                                 101
113179-17_02779_S_pmrC/1-547       DRSMITNILD TTPAESFALL SGEMIAVLGL SGVLAVFVAW WVKIRKPATR
16020166_02426_S_pmrC/1-547        DRSMITNILD TTPAESFALL SGEMIAVLGL SGVLAVFVAW WVKIRKPATR
808922-16_04523_S_pmrC/1-547       DRSMITNILD TTPAESFALL SGEMIAVLGL SGVLAVFVAW WVKIRKPATR
809097-16_03284_S_pmrC/1-547       DRSMITNILD TTPAESFALL SGEMIAVLGL SGVLAVFVAW WVKIRKPATR
13892823_03068_S_pmrC/1-547        DRSMITNILD TTPAESFALL SGEMIAVLGL SGVLAVFVAW WVKIRKPATR
14414149_00075_S_pmrC/1-547        DRSMITNILD TTPAESFALL SGEMIAVLGL SGVLAVFVAW WVKIRKPATR
18111299_00550_S_pmrC/1-547        DRSMITNILD TTPAESFALL SGEMIAVLGL SGVLAVFVAW WVKIRKPATR
19221887_04214_S_pmrC/1-547        DRSMITNILD TTPAESFALL SGEMIVVLGL SGVLAVLVAW WVKIRKPATL
809156-16_00536_R_pmrC/1-547       DRSMITNILD TTPAESFALL SGEMIAVLGL SGVLAVFVAW WVKIRKPATR
26048671-KSLU_03264_R_pmrC/1-547   DRSMITNILD TTPAESFALL SGEMIAVLGL SGVLAVFVAW WVKIRKPATR
401433-14_00075_R_pmrC/1-547       DRSMITNILD TTPAESFALL SGEMIAVLGL SGVLAVFVAW WVKIRKPATR
20038016-KSLU_00616_R_pmrC/1-547   DRSMITNILD TTPAESFALL SGEMIAVLGL SGVLAVFVAW WVKIRKPATR
402006-2-12_00566_R_pmrC/1-547     DRSMITNILD TTPAESFALL SGEMIAVLGL SGVLAVFVAW WVKIRKPATR
404507-16_02722_R_pmrC/1-547       DRSMITNILD TTPAESFALL SGEMIAVLGL SGVLAVFVAW WVKIRKPATR
808927-16_01793_R_pmrC/1-547       DRSMITNILD TTPAESFALL SGEMIAVLGL SGVLAVFVAW WVKIRKPATR
19852760_00689_R_pmrC/1-547        DRSMITNILD TTPAESFALL SGEMIAVLGL SGVLAVFVAW WVKIRKPATR
16003084_04037_R_pmrC/1-547        DRSMITNILD TTPAESFALL SGEMIAVLGL SGVLAVFVAW WVKIRKPATR
800138-16_01733_R_pmrC/1-547       DRSMITNILD TTPAESFALL SGEMIAVLGL SGVLAVFVAW WVKIRKPATR
18701876_02744_R_pmrC/1-547        DRSMITNILD TTPAESFALL SGEMIAVLGL SGVLAVFVAW WVKIRKPATR
802208-17_03226_R_pmrC/1-547       DRSMITNILD TTPAESFALL SGEMIAVLGL SGVLAVFVAW WVKIRKPATR
```

```
                                151
113179-17_02779_S_pmrC/1-547       WRGAAMRLLN IAVSALLIIL VAALFYKDYA SVFRNNKELV KSLSPSNSIV
16020166_02426_S_pmrC/1-547        WRGAAMRLLN IAVSALLIIL VAALFYKDYA SVFRNNKELV KSLSPSNSIV
808922-16_04523_S_pmrC/1-547       WRGAAMRLLN IAVSALLIIL VAALFYKDYA SVFRNNKELV KSLSPSNSIV
809097-16_03284_S_pmrC/1-547       WRGAAMRLLN IAVSALLIIL VAALFYKDYA SVFRNNKELV KSLSPSNSIV
13892823_03068_S_pmrC/1-547        WRGAAMRLLN IAVSALLIIL VAALFYKDYA SVFRNNKELV KSLSPSNSIV
14414149_00075_S_pmrC/1-547        WRGAAMRLLN IAVSALLIIL VAALFYKDYA SVFRNNKELV KSLSPSNSIV
18111299_00550_S_pmrC/1-547        WRGAAMRLLN IAVSALLIIL VAALFYKDYA SVFRNNKELV KSLSPSNSIV
19221887_04214_S_pmrC/1-547        WRGVAWRLVN MVASALLIVL IAVLFYKDYA SLFRNNKELV KSLSPSNSIV
809156-16_00536_R_pmrC/1-547       WRGAAMRLLN IAVSALLIIL VAALFYKDYA SVFRNNKELV KSLSPSNSIV
26048671-KSLU_03264_R_pmrC/1-547   WRGAAMRLLN IAVSALLIIL VAALFYKDYA SVFRNNKELV KSLSPSNSIV
401433-14_00075_R_pmrC/1-547       WRGAAMRLLN IAVSALLIIL VAALFYKDYA SVFRNNKELV KSLSPSNSIV
20038016-KSLU_00616_R_pmrC/1-547   WRGAAMRLLN IAVSALLIIL VAALFYKDYA SVFRNNKELV KSLSPSNSIV
402006-2-12_00566_R_pmrC/1-547     WRGAAMRLLN IAVSALLIIL VAALFYKDYA SVFRNNKELV KSLSPSNSIV
404507-16_02722_R_pmrC/1-547       WRGAAMRLLN IAVSALLIIL VAALFYKDYA SVFRNNKELV KSLSPSNSIV
808927-16_01793_R_pmrC/1-547       WHGAAMRLLN IAVSALLIIL VAALFYKDYA SVFRNNKELV KSLSPSNSIV
19852760_00689_R_pmrC/1-547        WRGAAMRLLN IAVSALLIIL VAALFYKDYA SVFRNNKELV KSLSPSNSIV
16003084_04037_R_pmrC/1-547        WRGAAMRLLN IAVSALLIIL VAALFYKDYA SVFRNNKELV KSLSPSNSIV
800138-16_01733_R_pmrC/1-547       WRGAAMRLLN IAVSALLIIL VAALFYKDYA SVFRNNKELV KSLSPSNSIV
18701876_02744_R_pmrC/1-547        WRGAAMRLLN IAVSALLIIL VAALFYKDYA SVFRNNKELV KSLSPSNSIV
802208-17_03226_R_pmrC/1-547       WRGAAMRLLN IAVSALLIIL VAALFYKDYA SVFRNNKELV KSLSPSNSIV

                                201
113179-17_02779_S_pmrC/1-547       AVNSWYAHHR MDNLPLVKIG EDATQKAVMH NAPRKNLTIV VLGETSRADN
16020166_02426_S_pmrC/1-547        AVNSWYAHHR MDNLPLVKIG EDATQKAVMH NAPRKNLTIV VLGETSRADN
808922-16_04523_S_pmrC/1-547       AVNSWYAHHR MDNLPLVKIG EDATQKAVMH NAPRKNLTIV VLGETSRADN
809097-16_03284_S_pmrC/1-547       AVNSWYAHHR MDNLPLVKIG EDATQKAVMH NAPRKNLTIV VLGETSRADN
13892823_03068_S_pmrC/1-547        AVNSWYAHHR MDNLPLVKIG EDATQKAVMH NAPRKNLTIV VLGETSRADN
14414149_00075_S_pmrC/1-547        AVNSWYAHHR MDNLPLVKIG EDATQKAVMH NAPRKNLTIV VLGETSRADN
18111299_00550_S_pmrC/1-547        AVNSWYAHHR MDNLPLVKIG EDATQKAVMH NAPRKNLTIV VLGETSRADN
19221887_04214_S_pmrC/1-547        AVNSWYAHHR MDNLPLVKIG EDAKQNPVMH NGPRKNLTIV VLGETSRADN
809156-16_00536_R_pmrC/1-547       AVNSWYAHHR MDNLPLVKIG EDATQKAVMH NAPRKNLTIV VLGETSRADN
26048671-KSLU_03264_R_pmrC/1-547   AVNSWYAHHR MDNLPLVKIG EDATQKAVMH NAPRKNLTIV VLGETSRADN
401433-14_00075_R_pmrC/1-547       AVNSWYAHHR MDNLPLVKIG EDATQKAVMH NAPRKNLTIV VLGETSRADN
20038016-KSLU_00616_R_pmrC/1-547   AVNSWYAHHR MDNLPLVKIG EDATQKAVMH NAPRKNLTIV VLGETSRADN
402006-2-12_00566_R_pmrC/1-547     AVNSWYAHHR MDNLPLVKIG EDATQKAVMH NAPRKNLTIV VLGETSRADN
404507-16_02722_R_pmrC/1-547       AVNSWYAHHR MDNLPLVKIG EDATQKAVMH NAPRKNLTIV VLGETSRADN
808927-16_01793_R_pmrC/1-547       AVNSWYAHHR MDNLPLVKIG EDATQKAVMH NAPRKNLTIV VLGETSRADN
19852760_00689_R_pmrC/1-547        AVNSWYAHHR MDNLPLVKIG EDATQKAVMH NAPRKNLTIV VLGETSRADN
16003084_04037_R_pmrC/1-547        AVNSWYAHHR MDNLPLVKIG EDATQKAVMH NAPRKNLTIV VLGETSRADN
800138-16_01733_R_pmrC/1-547       AVNSWYAHHR MDNLPLVKIG EDATQKAVMH NAPRKNLTIV VLGETSRADN
18701876_02744_R_pmrC/1-547        AVNSWYAHHR MDNLPLVKIG EDATQKAVMH NAPRKNLTIV VLGETSRADN
802208-17_03226_R_pmrC/1-547       AVNSWYAHHR MDNLPLVKIG EDATQKAVMH NAPRKNLTIV VLGETSRADN

                                251
113179-17_02779_S_pmrC/1-547       FSLGGYSRDT NPLMRQDGVI YFPHTTSCGT ATAVSVPCMF SNMPRAHYDE
16020166_02426_S_pmrC/1-547        FSLGGYLRDT NPLMRQDGVI YFPHTTSCGT ATAVSVPCMF SNMPRAHYDE
808922-16_04523_S_pmrC/1-547       FSLGGYSRDT NPLMRQDGVI YFPHTTSCGT ATAVSVPCMF SNMPRAHYDE
809097-16_03284_S_pmrC/1-547       FSLGGYSRDT NPLMRQDGVI YFPHTTSCGT ATAVSVPCMF SNMPRAHYDE
13892823_03068_S_pmrC/1-547        FSLGGYSRDT NPLMRQDGVI YFPHTTSCGT ATAVSVPCMF SNMPRAHYDE
14414149_00075_S_pmrC/1-547        FSLGGYSRDT NPLMRQDGVI YFPHTTSCGT ATAVSVPCMF SNMPRAHYDE
18111299_00550_S_pmrC/1-547        FSLGGYSRDT NPLMRQDGVI YFPHTTSCGT ATAVSVPCMF SNMPRAHYDE
19221887_04214_S_pmrC/1-547        FSLGGYPRDT NPLMQQDGVI YFPHTTSCGT ATAVSVPCMF SNMPRAHYDE
809156-16_00536_R_pmrC/1-547       FSLGGYSRDT NPLMRQDGVI YFPHTTSCGT ATAVSVPCMF SNMPRAHYDE
26048671-KSLU_03264_R_pmrC/1-547   FSLGGYLRDT NPLMRQDGVI YFPHTTSCGT ATAVSVPCMF SNMPRAHYDE
401433-14_00075_R_pmrC/1-547       FSLGGYSRDT NPLMRQDGVI YFPHTTSCGT ATAVSVPCMF SNMPRAHYDE
20038016-KSLU_00616_R_pmrC/1-547   FSLGGYSRDT NPLMRQDGVI YFPHTTSCGT ATAVSVPCMF SNMPRAHYDE
402006-2-12_00566_R_pmrC/1-547     FSLGGYSRDT NPLMRQDGVI YFPHTTSCGT ATAVSVPCMF SNMPRAHYDE
404507-16_02722_R_pmrC/1-547       FSLGGYSRDT NPLMRQDGVI YFPHTTSCGT ATAVSVPCMF SNMPRAHYDE
808927-16_01793_R_pmrC/1-547       FSLGGYSRDT NPLMRQDGVI YFPHTTSCGT ATAVSVPCMF SNMPRAHYDE
19852760_00689_R_pmrC/1-547        FSLGGYSRDT NPLMRQDGVI YFPHTTSCGT ATAVSVPCMF SNMPRAHYDE
16003084_04037_R_pmrC/1-547        FSLGGYSRDT NPLMRQDGVI YFPHTTSCGT ATAVSVPCMF SNMPRAHYDE
800138-16_01733_R_pmrC/1-547       FSLGGYLRDT NPLMRQDGVI YFPHTTSCGT ATAVSVPCMF SNMPRAHYDE
18701876_02744_R_pmrC/1-547        FSLGGYSRDT NPLMRQDGVI YFPHTTSCGT ATAVSVPCMF SNMPRAHYDE
802208-17_03226_R_pmrC/1-547       FSLGGYSRDT NPLMRQDGVI YFPHTTSCGT ATAVSVPCMF SNMPRAHYDE
```

```
                                   301
113179-17_02779_S_pmrC/1-547       ELAHHQEGVL DILQRAGIQV LWNDNDGGCK GACDRVPHQN VTDLKLTGQC
16020166_02426_S_pmrC/1-547        ELAHHQEGVL DILQRAGIQV LWNDNDGGCK GACDRVPHQN VTDLKLTGQC
808922-16_04523_S_pmrC/1-547       ELAHHQEGVL DILQRAGIQV LWNDNDGGCK GACDRVPHQN VTDLKLTGQC
809097-16_03284_S_pmrC/1-547       ELAHHQEGVL DILQRAGIRV LWNDNDGGCK GACDRVPHQN VTDLKLTGQC
13892823_03068_S_pmrC/1-547        ELAHHQEGVL DILQRAGIRV LWNDNDGGCK GACDRVPHQN VTDLKLTGQC
14414149_00075_S_pmrC/1-547        ELAHHQEGVL DILQRAGIRV LWNDNDGGCK GACDRVPHQN VTDLKLTGQC
18111299_00550_S_pmrC/1-547        ELAHHQEGVL DILQRAGIRV LWNDNDGGCK GACDRVPHQN VTDLKLTGQC
19221887_04214_S_pmrC/1-547        ELAHHQEGVL DILQRAGIQV LWNDNDGGCK GACDRVPHQN VTDLKLTGQC
809156-16_00536_R_pmrC/1-547       ELAHHQEGVL DILQRAGIRV LWNDNDGGCK GACDRVPHQN VTDLKLTGQC
26048671-KSLU_03264_R_pmrC/1-547   ELAHHQEGVL DILQRAGIQV LWNDNDGGCK GACDRVPHQN VTDLKLTGQC
401433-14_00075_R_pmrC/1-547       ELAHHQEGVL DILQRAGIRV LWNDNDGGCK GACDRVPHQN VTDLKLTGQC
20038016-KSLU_00616_R_pmrC/1-547   ELAHHQEGVL DILQRAGIRV LWNDNDGGCK GACDRVPHQN VTDLKLTGQC
402006-2-12_00566_R_pmrC/1-547     ELAHHQEGVL DILQRAGIRV LWNDNDGGCK GACDRVPHQN VTDLKLTGQC
404507-16_02722_R_pmrC/1-547       ELAHHQEGVL DILQRAGIRV LWNDNDGGCK GACDRVPHQN VTDLKLTGQC
808927-16_01793_R_pmrC/1-547       ELAHHQEGVL DILQRAGIQV LWNDNDGGCK GACDRVPHQN VTDLKLTGQC
19852760_00689_R_pmrC/1-547        ELAHHQEGVL DILQRAGIRV LWNDNDGGCK GACDRVPHQN VTDLKLTGQC
16003084_04037_R_pmrC/1-547        ELAHHQEGVL DILQRAGIRV LWNDNDGGCK GACDRVPHQN VTDLKLTGQC
800138-16_01733_R_pmrC/1-547       ELAHHQEGVL DILQRAGIQV LWNDNDGGCK GACDRVPHQN VTDLKLTGQC
18701876_02744_R_pmrC/1-547        ELAHHQEGVL DILQRAGIRV LWNDNDGGCK GACDRVPHQN VTDLKLTGQC
802208-17_03226_R_pmrC/1-547       ELAHHQEGVL DILQRAGIRV LWNDNDGGCK GACDRVPHQN VTDLKLTGQC

                                   351
113179-17_02779_S_pmrC/1-547       IDGECYDDVL FHNLDSYIDN LQQDGIIVLH TIGSHGPTYY NRYPAAFRKF
16020166_02426_S_pmrC/1-547        IDGECYDDVL FHNLDSYIDN LQQDGIIVLH TIGSHGPTYY NRYPAAFRKF
808922-16_04523_S_pmrC/1-547       IDGECYDDVL FHNLDSYIDN LQQDGIIVLH TIGSHGPTYY NRYPAAFRKF
809097-16_03284_S_pmrC/1-547       IDGECYDDVL FHNLDSYIDN LQQDGIIVLH TIGSHGPTYY NRYPAAFRKF
13892823_03068_S_pmrC/1-547        IDGECYDDVL FHNLDSYIDN LQQDGIIVLH TIGSHGPTYY NRYPAAFRKF
14414149_00075_S_pmrC/1-547        IDGECYDDVL FHNLDSYIDN LQQDGIIVLH TIGSHGPTYY NRYPAAFRKF
18111299_00550_S_pmrC/1-547        IDGECYDDVL FHNLDSYIDN LQQDGIIVLH TIGSHGPTYY NRYPAAFRKF
19221887_04214_S_pmrC/1-547        IDGECYDDVL FHHLDSYIDN LQQDGIIVLH TIGSHGPTYY NRYPAEFKKF
809156-16_00536_R_pmrC/1-547       IDGECYDDVL FHNLDSYIDN LQQDGIIVLH TIGSHGPTYY NRYPAAFRKF
26048671-KSLU_03264_R_pmrC/1-547   IDGECYDDVL FHNLDSYIDN LQQDGIIVLH TIGSHGPTYY NRYPAAFRKF
401433-14_00075_R_pmrC/1-547       IDGECYDDVL FHNLDSYIDN LQQDGIIVLH TIGSHGPTYY NRYPAAFRKF
20038016-KSLU_00616_R_pmrC/1-547   IDGECYDDVL FHNLDSYIDN LQQDGIIVLH TIGSHGPTYY NRYPAAFRKF
402006-2-12_00566_R_pmrC/1-547     IDGECYDDVL FHNLDSYIDN LQQDGIIVLH TIGSHGPTYY NRYPAAFRKF
404507-16_02722_R_pmrC/1-547       IDGECYDDVL FHNLDSYIDN LQQDGIIVLH TIGSHGPTYY NRYPAAFRKF
808927-16_01793_R_pmrC/1-547       IDGECYDDVL FHNLDSYIDN LQQDGIIVLH TIGSHGPTYY NRYPAAFRKF
19852760_00689_R_pmrC/1-547        IDGECYDDVL FHNLDSYIDN LQQDGIIVLH TIGSHGPTYY NRYPAAFRKF
16003084_04037_R_pmrC/1-547        IDGECYDDVL FHNLDSYIDN LQQDGIIVLH TIGSHGPTYY NRYPAAFRKF
800138-16_01733_R_pmrC/1-547       IDGECYDDVL FHNLDSYIDN LQQDGIIVLH TIGSHGPTYY NRYPAAFRKF
18701876_02744_R_pmrC/1-547        IDGECYDDVL FHNLDSYIDN LQQDGIIVLH TIGSHGPTYY NRYPAAFRKF
802208-17_03226_R_pmrC/1-547       IDGECYDDVL FHNLDSYIDN LQQDGIIVLH TIGSHGPTYY NRYPAAFRKF

                                   401
113179-17_02779_S_pmrC/1-547       TPTCDTNEIQ GCTREQLTNT YDNTILYVDY VVDKAIKLLQ SKQDKFTTSL
16020166_02426_S_pmrC/1-547        TPTCDTNEIQ GCTREQLTNT YDNTILYVDY VVDKAIKLLQ SKQDKFTTSL
808922-16_04523_S_pmrC/1-547       TPTCDTNEIQ GCTREQLTNT YDNTILYVDY VVDKAIKLLQ SKQDKFTTSL
809097-16_03284_S_pmrC/1-547       TPTCDTNEIQ GCTREQLTNT YDNTILYVDY VVDKAIKLLQ SKQDKFTTSL
13892823_03068_S_pmrC/1-547        TPTCDTNEIQ GCTREQLTNT YDNTILYVDY VVDKAIKLLQ SKQDKFTTSL
14414149_00075_S_pmrC/1-547        TPTCDTNEIQ GCTREQLTNT YDNTILYVDY VVDKAIKLLQ SKQDKFTTSL
18111299_00550_S_pmrC/1-547        TPTCDTNEIQ GCTREQLTNT YDNTILYVDY VVDKAIKLLQ SKQDKFTTSL
19221887_04214_S_pmrC/1-547        TPTCDTNEIQ GCTREQLTNT YDNTILYVDH VVDKAIKLLQ AKQDKFTTSL
809156-16_00536_R_pmrC/1-547       TPTCDTNEIQ GCTREQLTNT YDNTILYVDY VVDKAIKLLQ SKQDKFTTSL
26048671-KSLU_03264_R_pmrC/1-547   TPTCDTNEIQ GCTREQLTNT YDNTILYVDY VVDKAIKLLQ SKQDKFTTSL
401433-14_00075_R_pmrC/1-547       TPTCDTNEIQ GCTREQLTNT YDNTILYVDY VVDKAIKLLQ SKQDKFTTSL
20038016-KSLU_00616_R_pmrC/1-547   TPTCDTNEIQ GCTREQLTNT YDNTILYVDY VVDKAIKLLQ SKQDKFTTSL
402006-2-12_00566_R_pmrC/1-547     TPTCDTNEIQ GCTREQLTNT YDNTILYVDY VVDKAIKLLQ SKQDKFTTSL
404507-16_02722_R_pmrC/1-547       TPTCDTNEIQ GCTREQLTNT YDNTILYVDY VVDKAIKLLQ SKQDKFTTSL
808927-16_01793_R_pmrC/1-547       TPTCDTNEIQ GCTREQLTNT YDNTILYVDY VVDKAIKLLQ SKQDKFTTSL
19852760_00689_R_pmrC/1-547        TPTCDTNEIQ GCTREQLTNT YDNTILYVDY VVDKAIKLLQ SKQDKFTTSL
16003084_04037_R_pmrC/1-547        TPTCDTNEIQ GCTREQLTNT YDNTILYVDY VVDKAIKLLQ SKQDKFTTSL
800138-16_01733_R_pmrC/1-547       TPTCDTNEIQ GCTREQLTNT YDNTILYVDY VVDKAIKLLQ SKQDKFTTSL
18701876_02744_R_pmrC/1-547        TPTCDTNEIQ GCTREQLTNT YDNTILYVDY VVDKAIKLLQ SKQDKFTTSL
802208-17_03226_R_pmrC/1-547       TPTCDTNEIQ GCTREQLTNT YDNTILYVDY VVDKAIKLLQ SKQDKFTTSL
```

```
                                   451
113179-17_02779_S_pmrC/1-547        VYLSDHGESL GEDGVYLHGL PYSIAPDTQK HVPMALWLSA DYQQRYGISA
16020166_02426_S_pmrC/1-547         VYLSDHGESL GEDGVYLHGL PYSIAPDTQK HVPMALWLSA DYQQRYGISA
808922-16_04523_S_pmrC/1-547        VYLSDHGESL GEDGVYLHGL PYSIAPDTQK HVPMALWLSA DYQQRYGISA
809097-16_03284_S_pmrC/1-547        VYLSDHGESL GEDGVYLHGL PYSIAPDTQK HVPMALWLSA DYQQRYGISA
13892823_03068_S_pmrC/1-547         VYLSDHGESL GEDGVYLHGL PYSIAPDTQK HVPMALWLSA DYQQRYGISA
14414149_00075_S_pmrC/1-547         VYLSDHGESL GEDGVYLHGL PYSIAPDTQK HVPMALWLSA DYQQRYGISA
18111299_00550_S_pmrC/1-547         VYLSDHGESL GEDGVYLHGL PYSIAPDTQK HVPMALWLSA DYQQRYGISA
19221887_04214_S_pmrC/1-547         VYLSDHGESL GEDGVYLHGL PYSIAPDTQK HVPMVMWLSA DYQQRYGISA
809156-16_00536_R_pmrC/1-547        VYLSDHGESL GEDGVYLHGL PYSIAPDTQK HVPMALWLSA DYQQRYGISA
26048671-KSLU_03264_R_pmrC/1-547    VYLSDHGESL GEDGVYLHGL PYSIAPDTQK HVPMALWLSA DYQQRYGISA
401433-14_00075_R_pmrC/1-547        VYLSDHGESL GEDGVYLHGL PYSIAPDTQK HVPMALWLSA DYQQRYGISA
20038016-KSLU_00616_R_pmrC/1-547    VYLSDHGESL GEDGVYLHGL PYSIAPDTQK HVPMALWLSA DYQQRYGISA
402006-2-12_00566_R_pmrC/1-547      VYLSDHGESL GEDGVYLHGL PYSIAPDTQK HVPMALWLSA DYQQRYGISA
404507-16_02722_R_pmrC/1-547        VYLSDHGESL GEDGVYLHGL PYSIAPDTQK HVPMALWLSA DYQQRYGISA
808927-16_01793_R_pmrC/1-547        VYLSDHGESL GEDGVYLHGL PYSIAPNTQK HVPMALWLSA DYQQRYGISA
19852760_00689_R_pmrC/1-547         VYLSDHGESL GEDGVYLHGL PYSIAPDTQK HVPMALWLSA DYQQRYGISA
16003084_04037_R_pmrC/1-547         VYLSDHGESL GEDGVYLHGL PYSIAPDTQK HVPMALWLSA DYQQRYGISA
800138-16_01733_R_pmrC/1-547        VYLSDHGESL GEDGVYLHGL PYSIAPDTQK HVPMALWLSA DYQQRYGISA
18701876_02744_R_pmrC/1-547         VYLSDHGESL GEDGVYLHGL PYSIAPDTQK HVPMALWLSA DYQQRYGISA
802208-17_03226_R_pmrC/1-547        VYLSDHGESL GEDGVYLHGL PYSIAPDTQK HVPMALWLSA DYQQRYGISA

                                   501
113179-17_02779_S_pmrC/1-547        HCLQQRAQKE NYSQDNLFST LLGLLGVSTR EYQAADDILT PCREAG*
16020166_02426_S_pmrC/1-547         HCLQQRAQKE NYSQDNLFST LLGLLGVSTR EYQAADDILT PCREAG*
808922-16_04523_S_pmrC/1-547        HCLQQRAQKE NYSQDNLFST LLGLLGVSTR EYQAADDILT PCREAG*
809097-16_03284_S_pmrC/1-547        HCLQQRAQKE NYSQDNLFST LLGLLGVSTR EYQAADDILT PCREAG*
13892823_03068_S_pmrC/1-547         HCLQQRAQKE NYSQDNLFST LLGLLGVSTR EYQAADDILT PCREAG*
14414149_00075_S_pmrC/1-547         HCLQQRAQKE NYSQDNLFST LLGLLGVSTR EYQAADDILT PCREAG*
18111299_00550_S_pmrC/1-547         HCLQQRAQKE NYSQDNLFST LLGLLGVSTR EYQAADDILT PCREAG*
19221887_04214_S_pmrC/1-547         QCLQQRAKKE NYSQDNLFST LLGLLGVSTH EYQAADDILT PCREAG*
809156-16_00536_R_pmrC/1-547        HCLQQRAQKE NYSQDNLFST LLGLLGVSTR EYQAADDILT PCREAG*
26048671-KSLU_03264_R_pmrC/1-547    HCLQQRAQKE NYSQDNLFST LLGLLGVSTR EYQAADDILT PCREAG*
401433-14_00075_R_pmrC/1-547        HCLQQRAQKE NYSQDNLFST LLGLLGVSTR EYQAADDILT PCREAG*
20038016-KSLU_00616_R_pmrC/1-547    HCLQQRAQKE NYSQDNLFST LLGLLGVSTR EYQAADDILT PCREAG*
402006-2-12_00566_R_pmrC/1-547      HCLQQRAQKE NYSQDNLFST LLGLLGVSTR EYQAADDILT PCREAG*
404507-16_02722_R_pmrC/1-547        HCLQQRAQKE NYSQDNLFST LLGLLGVSTR EYQAADDILT PCREAG*
808927-16_01793_R_pmrC/1-547        HCLQQRAQKE NYSQDNLFST LLGLLGVSTR EYQAADDILT PCREAG*
19852760_00689_R_pmrC/1-547         HCLQQRAQKE NYSQDNLFST LLGLLGVSTR EYQAADDILT PCREAG*
16003084_04037_R_pmrC/1-547         HCLQQRAQKE NYSQDNLFST LLGLLGVSTR EYQAADDILT PCREAG*
800138-16_01733_R_pmrC/1-547        HCLQQRAQKE NYSQDNLFST LLGLLGVSTR EYQAADDILT PCREAG*
18701876_02744_R_pmrC/1-547         HCLQQRAQKE NYSQDNLFST LLGLLGVSTR EYQAADDILT PCREAG*
802208-17_03226_R_pmrC/1-547        HCLQQRAQKE NYSQDNLFST LLGLLGVSTR EYQAADDILT PCREAG*
```
