# Supplementary material for: Colistin resistance in Gram-negative bacteria analysed by five phenotypic assays and inference of the underlying genomic mechanisms
Source: BMC Microbiol. 2021 Nov 20;21:321. doi: 10.1186/s12866-021-02388-8 (PMC8605564; doi:10.1186/s12866-021-02388-8)
Supplement: Supplementary file 4 — Additional file 4. Escherichia coli protein sequence alignments.pdf showing the protein sequences alignments of all colistin resistance-related proteins analyzed in this study. [file 12866_2021_2388_MOESM4_ESM.pdf]

Alignment: D:\Documentos BASILEA\AMR LAB\Projects\Colistin resistance project\Genes\E. coli gen
Seaview [blocks=10 fontsize=10 A4] on Fri Aug 14 09:23:06 2020

	1				
108627-17_03753_S_pmrA/1-223	MKILIVEDDT	LLLQGLILAA	QTEGYACDGV	STARMAEQSL	EAGHYSLVVL
705889-17_02711_S_pmrA/1-223	MKILIVEDDT	LLLQGLILAA	QTEGYACDGV	TTARMAEQSL	EAGHYSLVVL
722306-16_01584_S_pmrA/1-223	MKILIVEDDT	LLLQGLILAA	QTEGYACDGV	STARMAEQSL	EAGHYSLVVL
14929959_02416_S_pmrA/1-223	MKILIVEDDT	LLLQGLILAA	QTEGYACDGV	TTARMAEQSL	EAGHYSLVVL
19015727_03069_S_pmrA/1-223	MKILIVEDDT	LLLQGLILAA	QTEGYACDGV	TTARMAEQSL	EAGHYSLVVL
ATCC-25922_00126_S_pmrA/1-223	MKILIVEDDT	LLLQGLILAA	QTEGYACDGV	STARMAEQSL	EAGHYSLVVL
108670-17_00865_S_pmrA/1-223	MKILIVEDDT	LLLQGLILAA	QTEGYACDGV	STARMAEQSL	EAGHYSLVVL
603351-17_04081_S_pmrA/1-223	MKILIVEDDT	LLLQGLILAA	QTEGYACDGV	STARMAEQSL	EAGHYSLVVL
703629-17_00412_S_pmrA/1-223	MKILIVEDDT	LLLQGLILAA	QTEGYACDGV	STARMAEQSL	EAGHYSLVVL
703692-17_03306_S_pmrA/1-223	MKILIVEDDT	LLLQGLILAA	QTEGYACDGV	STARMAEQSL	EAGHYSLVVL
703694-17_00940_S_pmrA/1-223	MKILIVEDDT	LLLQGLILAA	QTEGYACDGV	TTARMAEQSL	EAGHYSLVVL
705882-17_00428_S_pmrA/1-223	MKILIVEDDT	LLLQGLILAA	QTEGYACDGV	TTARMAEQSL	EAGHYSLVVL
705801-17_01829_S_pmrA/1-223	MKILIVEDDT	LLLQGLILAA	QTEGYACDGV	TTARMAEQSL	EAGHYSLVVL
709006-16_02799_R_pmrA/1-223	MKILIVEDDT	LLLQGLILAA	QTEGYACDGV	STARMAEQSL	EAGHYSLVVL
719645-16_03123_R_pmrA/1-223	MKILIVEDDT	LLLQGLILAA	QTEGYACDGV	TTARMAEQSL	EAGHYSLVVL
721296-16_02905_R_pmrA/1-223	MKILIVEDDT	LLLQGLILAA	QTEGYACDGV	TTARMAEQSL	EAGHYSLVVL
KP-37_03330_R_pmrA/1-223	MKILIVEDDT	LLLQGLILAA	QTEGYACDGV	TTARMAEQSL	EAGHYSLVVL
NCTC-13846_00783_R_pmrA/1-223	MKILIVEDDT	LLLQGLILAA	QTEGYACDGV	TTARMAEQSL	EAGHYSLVVL
700099-17_03187_R_pmrA/1-223	MKILIVEDDT	LLLQGLILAA	QTEGYACDGV	TTARMAEQSL	EAGHYSLVVL
700455-17_00208_R_pmrA/1-223	MKILIVEDDT	LLLQGLILAA	QTEGYACDGV	STARMAEQSL	EAGHYSLVVL
705498-12_03776_R_pmrA/1-223	MKILIVEDDT	LLLQGLILAA	QTEGYACDGV	STARMAEQSL	EAGHYSLVVL
706090-16_01484_R_pmrA/1-223	MKILIVEDDT	LLLQGLILAA	QTEGYACDGV	STARMAEQSL	EAGHYSLVVL
707671-17_00427_R_pmrA/1-223	MKILIVEDDT	LLLQGLILAA	QTEGYACDGV	TTARMAEQSL	EAGHYSLVVL

	51				
108627-17_03753_S_pmrA/1-223	DLGLPDEDGL	HFLARIRQKK	YTLPVLILTA	RDTLTDKIAG	LDVGADDYLV
705889-17_02711_S_pmrA/1-223	DLGLPDEDGL	HFLARIRQKK	YTLPVLILTA	RDTLTDKIAG	LDVGADDYLV
722306-16_01584_S_pmrA/1-223	DLGLPDEDGL	HFLARIRQKK	YTLPVLILTA	RDTLTDKIAG	LDVGADDYLV
14929959_02416_S_pmrA/1-223	DLGLPDEDGL	HFLARIRQKK	YTLPVLILTA	RDTLTDKIAG	LDVGADDYLV
19015727_03069_S_pmrA/1-223	DLGLPDEDGL	HFLARIRQKK	YTLPVLILTA	RDTLTDKIAG	LDVGADDYLV
ATCC-25922_00126_S_pmrA/1-223	DLGLPDEDGL	HFLARIRQKK	YTLPVLILTA	RDTLTDKIAG	LDVGADDYLV
108670-17_00865_S_pmrA/1-223	DLGLPDEDGL	HFLARIRQKK	YTLPVLILTA	RDTLTDKIAG	LDVGADDYLV
603351-17_04081_S_pmrA/1-223	DLGLPDEDGL	HFLARIRQKK	YTLPVLILTA	RDTLTDKIAG	LDVGADDYLV
703629-17_00412_S_pmrA/1-223	DLGLPDEDGL	HFLARIRQKK	YTLPVLILTA	RDTLTDKIAG	LDVGADDYLV
703692-17_03306_S_pmrA/1-223	DLGLPDEDGL	HFLARIRQKK	YTLPVLILTA	RDTLTDKIAG	LDVGADDYLV
703694-17_00940_S_pmrA/1-223	DLGLPDEDGL	HFLARIRQKK	YTLPVLILTA	RDTLTDKIAG	LDVGADDYLV
705882-17_00428_S_pmrA/1-223	DLGLPDEDGL	HFLARIRQKK	YTLPVLILTA	RDTLTDKIAG	LDVGADDYLV
705801-17_01829_S_pmrA/1-223	DLGLPDEDGL	HFLARIRQKK	YTLPVLILTA	RDTLTDKIAG	LDVGADDYLV
709006-16_02799_R_pmrA/1-223	DLGLPDEDGL	HFLARIRQKK	YTLPVLILTA	RDTLTDKIAG	LDVGADDYLV
719645-16_03123_R_pmrA/1-223	DLGLPDEDGL	HFLARIRQKK	YTLPVLILTA	RDTLTDKIAG	LDVGADDYLV
721296-16_02905_R_pmrA/1-223	DLGLPDEDGL	HFLARIRQKK	YTLPVLILTA	RDTLTDKIAG	LDVGADDYLV
KP-37_03330_R_pmrA/1-223	DLGLPDEDGL	HFLARIRQKK	YTLPVLILTA	RDTLTDKIAG	LDVGADDYLV
NCTC-13846_00783_R_pmrA/1-223	DLGLPDEDGL	HFLARIRQKK	YTLPVLILTA	RDTLTDKIAG	LDVGADDYLV
700099-17_03187_R_pmrA/1-223	DLGLPDEDGL	HFLARIRQKK	YTLPVLILTA	RDTLTDKIAG	LDVGADDYLV
700455-17_00208_R_pmrA/1-223	DLGLPDEDGL	HFLARIRQKK	YTLPVLILTA	RDTLTDKIAG	LDVGADDYLV
705498-12_03776_R_pmrA/1-223	DLGLPDEDGL	HFLARIRQKK	YTLPVLILTA	RDTLTDKIAG	LDVGADDYLV
706090-16_01484_R_pmrA/1-223	DLGLPDEDGL	HFLARIRQKK	YTLPVLILTA	HDTLTDKIAG	LDVGADDYLV
707671-17_00427_R_pmrA/1-223	DLGLPDEDGL	HFLARIRQKK	YTLPVLILTA	RDTLTDKIAG	LDVGADDYLV

	101				
108627-17_03753_S_pmrA/1-223	KPFALEELHA	RIRALLRRHN	NQGESELNVG	NLTLNMGRRQ	VWMSGEELIL
705889-17_02711_S_pmrA/1-223	KPFALEELHA	RIRALLRRHN	NQGESELIVG	NLTLNMGRRQ	VWMGGEELIL
722306-16_01584_S_pmrA/1-223	KPFALEELHA	RIRALLRRHN	NQGESELIVG	NLTLNMGRRQ	VWMGGEELIL
14929959_02416_S_pmrA/1-223	KPFALEELHA	RIRALLRRHN	NQGESELIVG	NLTLNMGRRQ	VWMGGEELIL
19015727_03069_S_pmrA/1-223	KPFALEELHA	RIRALLRRHN	NQGESELIVG	NLTLNMGRRQ	VWMGGEELIL
ATCC-25922_00126_S_pmrA/1-223	KPFALEELHA	RIRALLRRHN	NQGESELNVG	NLTLNMGRRQ	VWMSGEELIL
108670-17_00865_S_pmrA/1-223	KPFALEELHA	RIRALLRRHN	NQGESELNVG	NLTLNMGRRQ	VWMSGEELIL
603351-17_04081_S_pmrA/1-223	KPFALEELHA	RIRALLRRHN	NQGESELNVG	NLTLNMGRRQ	VWMSGEELIL
703629-17_00412_S_pmrA/1-223	KPFALEELHA	RIRALLRRHN	NQGESELNVG	NLTLNMGRRQ	VWMSGEELIL
703692-17_03306_S_pmrA/1-223	KPFALEELHA	RIRALLRRHN	NQGESELNVG	NLTLNMGRRQ	VWMSGEELIL
703694-17_00940_S_pmrA/1-223	KPFALEELHA	RIRALLRRHN	NQGESELIVG	NLTLNMGRRQ	VWMGGEELIL
705882-17_00428_S_pmrA/1-223	KPFALEELHA	RIRALLRRHN	NQGESELIVG	NLTLNMGRRQ	VWMGGEELIL
705801-17_01829_S_pmrA/1-223	KPFALEELHA	RIRALLRRHN	NQGESELIVG	NLTLNMGRRQ	VWMSGEELIL
709006-16_02799_R_pmrA/1-223	KPFALEELHA	RIRALLRRHN	NQGESELIVG	NLTLNMGRRQ	VWMGGEELIL
719645-16_03123_R_pmrA/1-223	KPFALEELHA	RIRALLRRHN	NQGESELIVG	NLTLNMGRRQ	VWMSGEELIL
721296-16_02905_R_pmrA/1-223	KPFALEELHA	RIRALLRRHN	NQGESELIVG	NLTLNMGRRQ	VWMGGEELIL
KP-37_03330_R_pmrA/1-223	KPFALEELHA	RIRALLRRHN	NQGESELIVG	NLTLNMGRRQ	VWMGGEELIL
NCTC-13846_00783_R_pmrA/1-223	KPFALEELHA	RIRALLRRHN	NQGESELIVG	NLTLNMGRRQ	VWMGGEELIL
700099-17_03187_R_pmrA/1-223	KPFALEELHA	RIRALLRRHN	NQGESELIVG	NLTLNMGRRQ	VWMSGEELIL
700455-17_00208_R_pmrA/1-223	KPFALEELHA	RIRALLRRHN	NQGESELNVG	NLTLNMGRRQ	VWMSGEELIL
705498-12_03776_R_pmrA/1-223	KPFALEELHA	RIRALLRRHN	NQGESELIVG	NLTLNMGRRQ	VWMGGEELIL
706090-16_01484_R_pmrA/1-223	KPFALEELHA	RIRALLRRHN	NQGESELNVG	NLTLNMGRRQ	VWMSGEELIL
707671-17_00427_R_pmrA/1-223	KPFALEELHA	RIRALLRRHN	NQGESELIVG	NLTLNMGRRQ	VWMGGEELIL

```
                                151
108627-17_03753_S_pmrA/1-223      TPKEYALLSR LMLKAGSPVH REILYNDIYN WDNEPSTNTL EVHIHNLRDK
705889-17_02711_S_pmrA/1-223      TPKEYALLSR LMLKAGSPVH REILYNDIYN WDNEPSTNTL EVHIHNLRDK
722306-16_01584_S_pmrA/1-223      TPKEYALLSR LMLKAGSPVH REILYNDIYN WDNEPSTNTL EVHIHNLRDK
14929959_02416_S_pmrA/1-223       TPKEYALLSR LMLKAGSPVH REILYNDIYN WDNEPSTNTL EVHIHNLRDK
19015727_03069_S_pmrA/1-223       TPKEYALLSR LMLKAGSPVH REILYNDIYN WDNEPSTNTL EVHIHNLRDK
ATCC-25922_00126_S_pmrA/1-223     TPKEYALLSR LMLKAGSPVH REILYNDIYN WDNEPSTNTL EVHIHNLRDK
108670-17_00865_S_pmrA/1-223      TPKEYALLSR LMLKAGSPVH REILYNDIYN WDNEPSTNTL EVHIHNLRDK
603351-17_04081_S_pmrA/1-223      TPKEYALLSR LMLKAGSPVH REILYNDIYN WDNEPSTNTL EVHIHNLRDK
703629-17_00412_S_pmrA/1-223      TPKEYALLSR LMLKAGSPVH REILYNDIYN WDNEPSTNTL EVHIHNLRDK
703692-17_03306_S_pmrA/1-223      TPKEYALLSR LMLKAGSPVH REILYNDIYN WDNEPSTNTL EVHIHNLRDK
703694-17_00940_S_pmrA/1-223      TPKEYALLSR LMLKAGSPVH REILYNDIYN WDNEPSTNTL EVHIHNLRDK
705882-17_00428_S_pmrA/1-223      TPKEYALLSR LMLKAGSPVH REILYNDIYN WDNEPSTNTL EVHIHNLRDK
705801-17_01829_S_pmrA/1-223      TPKEYALLSR LMLKAGSPVH REILYNDIYN WDNEPSTNTL EVHIHNLRDK
709006-16_02799_R_pmrA/1-223      TPKEYALLSR LMLKAGSPVH REILYNDIYN WDNEPSTNTL EVHIHNLRDK
719645-16_03123_R_pmrA/1-223      TPKEYALLSR LMLKAGSPVH REILYNDIYN WDNEPSTNTL EVHIHNLRDK
721296-16_02905_R_pmrA/1-223      TPKEYALLSR LMLKAGSPVH REILYNDIYN WDNEPSTNTL EVHIHNLRDK
KP-37_03330_R_pmrA/1-223          TPKEYALLSR LMLKAGSPVH REILYNDIYN WDNEPSTNTL EVHIHNLRDK
NCTC-13846_00783_R_pmrA/1-223     TPKEYALLSR LMLKAGSPVH REILYNDIYN WDNEPSTNTL EVHIHNLRDK
700099-17_03187_R_pmrA/1-223      TPKEYALLSR LMLKAGSPVH REILYNDIYN WDNEPSTNTL EVHIHNLRDK
700455-17_00208_R_pmrA/1-223      TPKEYALLSR LMLKAGSPVH REILYNDIYN WDNEPSTNTL EVHIHNLRDK
705498-12_03776_R_pmrA/1-223      TPKEYALLSR LMLKAGSPVH REILYNDIYN WDNEPSTNTL EVHIHNLRDK
706090-16_01484_R_pmrA/1-223      TPKEYALLSR LMLKAGSPVH REILYNDIYN WDNEPSTNTL EVHIHNLRDK
707671-17_00427_R_pmrA/1-223      TPKEYALLSR LMLKAGSPVH REILYNDIYN WDNEPSTNTL EVHIHNLRDK
```

```
                                201
108627-17_03753_S_pmrA/1-223      VGKARIRTVR GFGYMLVANE EN*
705889-17_02711_S_pmrA/1-223      VGKARIRTVR GFGYMLVANE EN*
722306-16_01584_S_pmrA/1-223      VGKARIRTVR GFGYMLVANE EN*
14929959_02416_S_pmrA/1-223       VGKARIRTVR GFGYMLVANE EN*
19015727_03069_S_pmrA/1-223       VGKARIRTVR GFGYMLVANE EN*
ATCC-25922_00126_S_pmrA/1-223     VGKARIRTVR GFGYMLVANE EN*
108670-17_00865_S_pmrA/1-223      VGKARIRTVR GFGYMLVANE EN*
603351-17_04081_S_pmrA/1-223      VGKARIRTVR GFGYMLVANE EN*
703629-17_00412_S_pmrA/1-223      VGKARIRTVR GFGYMLVANE EN*
703692-17_03306_S_pmrA/1-223      VGKARIRTVR GFGYMLVANE EN*
703694-17_00940_S_pmrA/1-223      VGKARIRTVR GFGYMLVANE EN*
705882-17_00428_S_pmrA/1-223      VGKARIRTVR GFGYMLVANE EN*
705801-17_01829_S_pmrA/1-223      VGKARIRTVR GFGYMLVANE EN*
709006-16_02799_R_pmrA/1-223      VGKARIRTVR GFGYMLVANE EN*
719645-16_03123_R_pmrA/1-223      VGKARIRTVR GFGYMLVANE EN*
721296-16_02905_R_pmrA/1-223      VGKARIRTVR GFGYMLVANE EN*
KP-37_03330_R_pmrA/1-223          VGKARIRTVR GFGYMLVANE EN*
NCTC-13846_00783_R_pmrA/1-223     VGKARIRTVR GFGYMLVANE EN*
700099-17_03187_R_pmrA/1-223      VGKARIRTVR GFGYMLVANE EN*
700455-17_00208_R_pmrA/1-223      VGKARIRTVR GFGYMLVANE EN*
705498-12_03776_R_pmrA/1-223      VGKARIRTVR GFGYMLVANE EN*
706090-16_01484_R_pmrA/1-223      VGKARIRTVR GFGYMLVANE EN*
707671-17_00427_R_pmrA/1-223      VGKARIRTVR GFGYMLVANE EN*
```

```
Alignment: D:\Documentos BASILEA\AMR LAB\Projects\Colistin resistance project\Genes\E. coli gen
Seaview [blocks=10 fontsize=10 A4] on Fri Aug 14 09:43:28 2020

                                   1
108627-17_03754_S_pmrB/1-367       LNLMRFLRRP ISLRQRLILT IGAILLVFEL ISVFWLWHES TEQIQLFEQA
19015727_03068_S_pmrB/1-367        LNLMHFLRRP ISLRQRLILT IGAILLVFEL ISVFWLWHES TEQIQLFEQA
ATCC-25922_00125_S_pmrB/1-367      LNLMRFLRRP ISLRQRLILT IGAILLVFEL ISVFWLWHES TEQIQLFEQA
108670-17_00866_S_pmrB/1-367       LNLMRFLRRP ISLRQRLILT IGAILLVFEL ISVFWLWHES TEQIQLFEQA
603351-17_04082_S_pmrB/1-367       LNLMRFLRRP ISLRQRLILT IGAILLVFEL ISVFWLWHES TEQIQLFEQA
703629-17_00411_S_pmrB/1-367       LNLMRFLRRP ISLRQRLILT IGAILLVFEL ISVFWLWHES TEQIQLFEQA
703692-17_03305_S_pmrB/1-367       LNLMRFLRRP ISLRQRLILT IGAILLVFEL ISVFWLWHES TEQIQLFEQA
703694-17_00941_S_pmrB/1-367       LNLMHFLRRP ISLRQRLILT IGAILLVFEL ISVFWLWHES TEQIQLFEQA
705882-17_00429_S_pmrB/1-367       LNLMRFLRRP ISLRQRLILT IGAILLVFEL ISVFWLWHES TEQIQLFEQA
705889-17_02710_S_pmrB/1-367       LNLMHFLRRP ISLRQRLILT IGAILLVFEL ISVFWLWHES TEQIQLFEQA
722306-16_01583_S_pmrB/1-367       LNLIRFLRRP ISLRQRLILT IGAILLVFEL ISVFWLWHES TEQIQLFEQA
14929959_02417_S_pmrB/1-367        LNLMRFLRRP ISLRQRLILT IGAILLVFEL ISVFWLWHES TEQIQLFEQA
705801-17_01828_S_pmrB/1-367       LNLMRFLRRP ISLRQRLILT IGAILLVFEL ISVFWLWHES TEQIQLFEQA
KP-37_03331_R_pmrB/1-367           LNLMHFLRRP ISLRQRLILT IGAILLVFEL ISVFWLWHES TEQIQLFEQA
NCTC-13846_00784_R_pmrB/1-367      LNLMRFLRRP ISLRQRLILT IGAILLVFEL ISVFWLWHES TEQIQLFEQA
700099-17_03188_R_pmrB/1-367       LNLMHFLRRP ISLRQRLILT IGAILLVFEL ISVFWLWHES TEQIQLFEQA
700455-17_00209_R_pmrB/1-367       LNLMRFLRRP ISLRQRLILT IGAILLVFEL ISVFWLWHES TEQIQLFEQA
705498-12_03777_R_pmrB/1-367       LNLMRFLRRP ISLRQRLILT IGAILLVFEL ISVFWLWHES TEQIQLFEQA
706090-16_01485_R_pmrB/1-367       LNLMRFLRRP ISLRQRLILT IGAILLVFEL ISVFWLWHES TEQIQLFEQA
707671-17_00428_R_pmrB/1-367       LNLMHFLRRP ISLRQRLILT IGAILLVFEL ISVFWLWHES TEQIQLFEQA
719645-16_03122_R_pmrB/1-367       LNLMHFLRRP ISLRQRLILT IGAILLVFEL ISVFWLWHES TEQIQLFEQA
709006-16_02800_R_pmrB/1-367       LNLMRFLRRP ISLRQRLILT IGAILLVFEL ISVFWLWHES TEQIQLFEQA
721296-16_02904_R_pmrB/1-367       LNLMHFLRRP ISLRQRLILT IGAILLVFEL ISVFWLWHES TEQIQLFEQA

                                  51
108627-17_03754_S_pmrB/1-367       LRDNRNNDRH IMREIREAVA SLIVPGVFMV SLTLFICYQA VRRITRPLAE
19015727_03068_S_pmrB/1-367        LRDNRNNDRH IMREIREAVA SLIVPGVFMV SLTLFICYQA VRRITRPLAE
ATCC-25922_00125_S_pmrB/1-367      LRDNRNNDRH IMREIREAVA SLIVPGVFMV SLTLFICYQA VRRITRPLAE
108670-17_00866_S_pmrB/1-367       LRDNRNNDRH IMREIREAVA SLIVPGVFMV SLTLFICYQA VRRITRPLAE
603351-17_04082_S_pmrB/1-367       LRDNRNNDRH IMREIREAVA SLIVPGVFMV SLTLFICYQA VRRITRPLAE
703629-17_00411_S_pmrB/1-367       LRDNRNNDRH IMREIREAVA SLIVPGVFMV SLTLFICYQA VRRITRPLAE
703692-17_03305_S_pmrB/1-367       LRDNRNNDRH IMREIREAVA SLIVPGVFMV SLTLFICYQA VRRITRPLAE
703694-17_00941_S_pmrB/1-367       LRDNRNNDRH IMREIREAVA SLIVPGVFMV SLTLFICYQA VRRITRPLAE
705882-17_00429_S_pmrB/1-367       LRDNRNNDRH IMREIREAVA SLIVPGVFMV SLTLFICYQA VRRITRPLAE
705889-17_02710_S_pmrB/1-367       LRDNRNNDRH IMREIREAVA SLIVPGVFMV SLTLFICYQA VRRITRPLAE
722306-16_01583_S_pmrB/1-367       LRDNRNNDRH IMREIREAVA SLIVPGVFMV SLTLFICYQA VRRITRPLAE
14929959_02417_S_pmrB/1-367        LRDNRNNDRH IMREIREAVA SLIVPGVFMV SLTLFICYQA VRRITRPLAE
705801-17_01828_S_pmrB/1-367       LRDNRNNDRH IMREIREAVA SLIVPGVFMV SLTLFICYQA VRRITRPLAE
KP-37_03331_R_pmrB/1-367           LRDNRNNDRH IMREIREAVA SLIVPGVFMV SLTLFICYQA VRRITRPLAE
NCTC-13846_00784_R_pmrB/1-367      LRDNRNNDRH IMREIREAVA SLIVPGVFMV SLTLFICYQA VRRITRPLAE
700099-17_03188_R_pmrB/1-367       LRDNRNNDRH IMREIREAVA SLIVPGVFMV SLTLFICYQA VRRITRPLAE
700455-17_00209_R_pmrB/1-367       LRDNRNNDRH IMREIREAVA SLIVPGVFMV SLTLFICYQA VRRITRPLAE
705498-12_03777_R_pmrB/1-367       LRDNRNNDRH IMREIREAVA SLIVPGVFMV SLTLFICYQA VRRITRPLAE
706090-16_01485_R_pmrB/1-367       LRDNRNNDRH IMREIREAVA SLIVPGVFMV SLTLFICYQA VRRITRPLAE
707671-17_00428_R_pmrB/1-367       LRDNRNNDRH IMREIREAVA SLIVPGVFMV SLTLFICYQA VRRITRPLAE
719645-16_03122_R_pmrB/1-367       LRDNRNNDRH IMREIREAVA SLIVPGVFMV SLTLFICYQA VRRITRPLAE
709006-16_02800_R_pmrB/1-367       LRDNRNNDRH IMREIREAVA SLIVPGVFMV SLTLFICYQA VRRITRPLAE
721296-16_02904_R_pmrB/1-367       LRDNRNNDRH IMREIREAVA SLIVPGVFMV SLTLFICYQA VRRITRLLAE

                                 101
108627-17_03754_S_pmrB/1-367       LQKELEARTA DNLTPIAIHS ATLEIDAVVS ALNDLVSRLT STLDNERLFT
19015727_03068_S_pmrB/1-367        LQKELEARTA DNLTPIAIHS ATLEIEAVVS ALNDLVSRLT STLDNERLFT
ATCC-25922_00125_S_pmrB/1-367      LQKELEARTA DNLTPIAIHS ATLEIDAVVS ALNDLVSRLT STLDNERLFT
108670-17_00866_S_pmrB/1-367       LQKELEARTA DNLTPIAIHS ATLEIDAVVS ALNDLVSRLT STLDNERLFT
603351-17_04082_S_pmrB/1-367       LQKELEARTA DNLTPIAIHS ATLEIDAVVS ALNDLVSRLT STLDNERLFT
703629-17_00411_S_pmrB/1-367       LQKELEARTA DNLTPIAIHS ATLEIDAVVS ALNDLVSRLT STLDNERLFT
703692-17_03305_S_pmrB/1-367       LQKELEARTA DNLTPIAIHS ATLEIDAVVS ALNDLVSRLT STLDNERLFT
703694-17_00941_S_pmrB/1-367       LQKELEARTA DNLTPIAIHS ATLEIEAVVS ALNDLVSRLT STLDNERLFT
705882-17_00429_S_pmrB/1-367       LQKELEARTA DNLTPIAIHS ATLEIEAVVS ALNDLVSRLT NTLDNERLFT
705889-17_02710_S_pmrB/1-367       LQKELEARTA DNLTPIAIHS ATLEIEAVVS ALNDLVSRLT STLDNERLFT
722306-16_01583_S_pmrB/1-367       LQKELEARTA DNLTPIAIHS ATLEIEAVVS ALNDLVSRLT STLDNERLFT
14929959_02417_S_pmrB/1-367        LQKELEARTA DNLTPIAIHS ATLEIEAVVS ALNDLVSRLT STLDNERLFT
705801-17_01828_S_pmrB/1-367       LQKELEARTA DNLTPIAIHS ATLEIEAVVS ALNDLVSRLT STLDNERLFT
KP-37_03331_R_pmrB/1-367           LQKELEARTA DNLTPIAIHS ATLEIEAVVS ALNDLVSRLT STLDNERLFT
NCTC-13846_00784_R_pmrB/1-367      LQKELEARTA DNLTPIAIHS ATLEIEAVVS ALNDLVSRLT STLDNERLFT
700099-17_03188_R_pmrB/1-367       LQKELEARTA DNLTPIAIHS ATLEIEAVVS ALNDLVSRLT STLDNERLFT
700455-17_00209_R_pmrB/1-367       LQKELEARTA DNLTPIAIHS ATLEIDAVVS ALNDLVSRLT STLDNERLFT
705498-12_03777_R_pmrB/1-367       LQKELEARTA DNLTPIAIHS ATLEIDAVVS ALNDLVSRLT STLDNERLFT
706090-16_01485_R_pmrB/1-367       LQKELEARTA DNLTPIAIHS ATLEIDAVVS ALNDLVSRLT STLDNERLFT
707671-17_00428_R_pmrB/1-367       LQKELEARTA DNLTPIAIHS ATLEIEAVVS ALNDLVSRLT STLDNERLFT
719645-16_03122_R_pmrB/1-367       LQKELEARTA DNLTPIAIHS ATLEIEAVVS ALNDLVSRLT STLDNERLFT
709006-16_02800_R_pmrB/1-367       LQKELEARTA DNLTPIAIHS ATLEIDAVVS ALNDLVSRLT STLDNERLFT
721296-16_02904_R_pmrB/1-367       LQKELEARTA DNLTPIAIHS ATLEIEAVVS ALNDLVSRLT STLDNERLFT
```

```
                                151
108627-17_03754_S_pmrB/1-367    ADVAHELRTP LAGVRLHLEL LAKTHHIDVA PLVARLDQMM ESVSQLLQLA
19015727_03068_S_pmrB/1-367     ADVAHELRTP LAGVRLHLEL LAKTHHIDVA PLVARLDQMM ESVSQLLQLA
ATCC-25922_00125_S_pmrB/1-367   ADVAHELRTP LAGVRLHLEL LAKTHHIDVA PLVARLDQMM ESVSQLLQLA
108670-17_00866_S_pmrB/1-367    ADVAHELRTP LAGVRLHLEL LAKTHHIDVA PLVARLDQMM ESVSQLLQLA
603351-17_04082_S_pmrB/1-367    ADVAHELRTP LAGVRLHLEL LAKTHHIDVA PLVARLDQMM ESVSQLLQLA
703629-17_00411_S_pmrB/1-367    ADVAHELRTP LAGVRLHLEL LAKTHHIDVA PLVARLDQMM ESVSQLLQLA
703692-17_03305_S_pmrB/1-367    ADVAHELRTP LAGVRLHLEL LAKTHHIDVA PLVARLDQMM ESVSQLLQLA
703694-17_00941_S_pmrB/1-367    ADVAHELRTP LAGVRLHLEL LAKTHHIDVA PLVARLDQMM ESVSQLLQLA
705882-17_00429_S_pmrB/1-367    ADVAHELRTP LAGVRLHLEL LAKTHHIDVA PLVARLDQMM ESVSQLLQLA
705889-17_02710_S_pmrB/1-367    ADVAHELRTP LAGVRLHLEL LAKTHHIDVA PLVARLDQMM ESVSQLLQLA
722306-16_01583_S_pmrB/1-367    ADVAHELRTP LAGVRLHLEL LAKTHHIDVA SLVARLDQMM ESVSQLLQLA
14929959_02417_S_pmrB/1-367     ADVAHELRTP LAGVRLHLEL LAKTHHIDVA PLVARLDQMM ESVSQLLQLA
705801-17_01828_S_pmrB/1-367    ADVAHELRTP LAGVRLHLEL LAKTHHIDVA PLVARLDQMM ESVSQLLQLA
KP-37_03331_R_pmrB/1-367        ADVAHELRTP LAGVRLHLEL LAKTHHIDVA PLVARLDQMM ESVSQLLQLA
NCTC-13846_00784_R_pmrB/1-367   ADVAHELRTP LAGVRLHLEL LAKTHHIDVA PLVARLDQMM ESVSQLLQLA
700099-17_03188_R_pmrB/1-367    ADVAHELRTP LAGVRLHLEL LAKTHHIDVA PLVARLDQMM ESVSQLLQLA
700455-17_00209_R_pmrB/1-367    ADVAHELRTP LAGVRLHLEL LAKTHHIDVA PLVARLDQMM ESVSQLPQLA
705498-12_03777_R_pmrB/1-367    ADVAHELRTP LAGVRLHLEL LAKTHHIDVA PLVARLDQMM ESVSQLLQLA
706090-16_01485_R_pmrB/1-367    ADVAHELRTP LAGVRLHLEL LAKTHHIDVA PLVARLDQMM ESVSQLLQLA
707671-17_00428_R_pmrB/1-367    ADVAHELRTP LAGVRLHLEL LAKTHHIDVA PLVARLDQMM ESVSQLLQLA
719645-16_03122_R_pmrB/1-367    ADVAHELRTP LAGVRLHLEL LAKTHHIDVA PLVARLDQMM ESVSQLLQLA
709006-16_02800_R_pmrB/1-367    ADVAHELRTP LAGVRLHLKL LAKTHHIDVA PLVARLDQMM ESVSQLLQLA
721296-16_02904_R_pmrB/1-367    ADVAHELRTP LAGVRLHLEL LAKTHHIDVA PLVARLDQMM ESVSQLLQLA

                                201
108627-17_03754_S_pmrB/1-367    RAGQSFSSGN YQHVKLLEDV ILPSYDELST MLDQRQQTLL LPESAADITV
19015727_03068_S_pmrB/1-367     RAGQSFSSGN YQHVKLLEDV ILPSYDELST MLDQRQQTLL LPESAADITV
ATCC-25922_00125_S_pmrB/1-367   RAGQSFSSGN YQHVKLLEDV ILPSYDELST MLDQRQQTLL LPESAADITV
108670-17_00866_S_pmrB/1-367    RAGQSFSSGN YQHVKLLEDV ILPSYDELST MLDQRQQTLL LPESAADITV
603351-17_04082_S_pmrB/1-367    RAGQSFSSGN YQHVKLLEDV ILPSYDELST MLDQRQQTLL LPESAADITV
703629-17_00411_S_pmrB/1-367    RAGQSFSSGN YQHVKLLEDV ILPSYDELST MLDQRQQTLL LPESAADITV
703692-17_03305_S_pmrB/1-367    RAGQSFSSGN YQHVKLLEDV ILPSYDELST MLDQRQQTLL LPESAADITV
703694-17_00941_S_pmrB/1-367    RAGQSFSSGN YQHVKLLEDV ILPSYDELST MLDQRQQTLL LPESAADITV
705882-17_00429_S_pmrB/1-367    RAGQSFSSGN YQHVKLLEDV ILPSYDELST MLDQRQQTLL LPESAADITV
705889-17_02710_S_pmrB/1-367    RAGQSFSSGN YQHVKLLEDV ILPSYDELST MLDQRQQTLL LPESAADITV
722306-16_01583_S_pmrB/1-367    RAGQSFSSGN YQHVKLLEDV ILPSYDELST MLDQRQQNLL LPESAADITV
14929959_02417_S_pmrB/1-367     RAGQSFSSGN YQHVKLLEDV ILPSYDELST MLDQRQQTLL LPESAADITV
705801-17_01828_S_pmrB/1-367    RAGQSFSSGN YQHVKLLEDV ILPSYDELST MLDQRQQTLL LPESAADITV
KP-37_03331_R_pmrB/1-367        RAGQSFSSGN YQHVKLLEDV ILPSYDELST MLDQRQQTLL LPESAADITV
NCTC-13846_00784_R_pmrB/1-367   RAGQSFSSGN YQHVKLLEDV ILPSYDELST MLDQRQQTLL LPESAADITV
700099-17_03188_R_pmrB/1-367    RAGQSFSSGN YQHVKLLEDV ILPSYDELST MLDQRQQTLL LPESAADITV
700455-17_00209_R_pmrB/1-367    RAGQSFSSGN YQHVKLLEDV ILPSYDELST MLDQRQQTLL LPESAADITV
705498-12_03777_R_pmrB/1-367    RAGQSFSSGN YQHVKLLEDV ILPSYDELST MLDQRQQTLL LPESAADITV
706090-16_01485_R_pmrB/1-367    RAGQSFSSGN YQHVKLLEDV ILPSYDELST MLDQRQQTLL LPESAADITV
707671-17_00428_R_pmrB/1-367    RAGQSFSSGN YQHVKLLEDV ILPSYDELST MLDQRQQTLL LPESAADITV
719645-16_03122_R_pmrB/1-367    RAGQSFSSGN YQHVKLLEDV ILPSYDELST MLDQRQQTLL LPESAADITV
709006-16_02800_R_pmrB/1-367    RAGQSFSSGN YQHVKLLEDV ILPSYDELST MLDQRQQTLL LPESAADITV
721296-16_02904_R_pmrB/1-367    RAGQSFSSGN YQHVKLLEDV ILPSYDELST MLDQRQQTLL LPESAADITV

                                251
108627-17_03754_S_pmrB/1-367    QGDATLLRML LRNLVENAHR YSPQGSNIMI KLQEDGGAVM AVEDEGPGID
19015727_03068_S_pmrB/1-367     QGDATLLRML LRNLVENAHR YSPQGSNIMI KLQEDGGAVM AVEDEGPGID
ATCC-25922_00125_S_pmrB/1-367   QGDATLLRML LRNLVENAHR YSPQGSNIMI KLQEDGGAVM AVEDEGPGID
108670-17_00866_S_pmrB/1-367    QGDATLLRML LRNLVENAHR YSPQGSNIMI KLQEDGGAVM AVEDEGPGID
603351-17_04082_S_pmrB/1-367    QGDATLLRML LRNLVENAHR YSPQGSNIMI KLQEDGGAVM AVEDEGPGID
703629-17_00411_S_pmrB/1-367    QGDATLLRML LRNLVENAHR YSPQGSNIMI KLQEDGGAVM AVEDEGPGID
703692-17_03305_S_pmrB/1-367    QGDATLLRML LRNLVENAHR YSPQGSNIMI KLQEDGGAVM AVEDEGPGID
703694-17_00941_S_pmrB/1-367    QGDATLLRML LRNLVENAHR YSPQGSNIMI KLQEDGGAVM AVEDEGPGID
705882-17_00429_S_pmrB/1-367    QGDATLLRML LRNLVENAHR YSPQGSNIMI KLQEDGGAVM AVEDEGPGID
705889-17_02710_S_pmrB/1-367    QGDATLLRML LRNLVENAHR YSPQGSNIMI KLQEDDGAVM AVEDEGPGID
722306-16_01583_S_pmrB/1-367    QGDATLLRML LRNLVENAHR YSPQGSNIMI KLQEDGGAVM AVEDEGPGID
14929959_02417_S_pmrB/1-367     QGDATLLRML LRNLVENAHR YSPQGSNIMI KLQEDGGAVM AVEDEGPGID
705801-17_01828_S_pmrB/1-367    QGDATLLRML LRNLVENAHR YSPQGSNIMI KLQEDGGAVM AVEDEGPGID
KP-37_03331_R_pmrB/1-367        QGDATLLRML LRNLVENAHR YSPQGSNIMI KLQEDDGAVM AVEDEGPGID
NCTC-13846_00784_R_pmrB/1-367   QGDATLLRML LRNLVENAHR YSPQGSNIMI KLQEDGGAVM AVEDEGPGID
700099-17_03188_R_pmrB/1-367    QGDATLLRML LRNLVENAHR YSPQGSNIMI KLQEDGGAVM AVEDEGPGID
700455-17_00209_R_pmrB/1-367    QGDATLLRML LRNLVENAHR YSPQGSNIMI KLQEDGGAVM AVEDEGPGID
705498-12_03777_R_pmrB/1-367    QGDATLLRML LRNLVENAHR YSPQGSNIMI KLQEDGGAVM AVEDEGPGID
706090-16_01485_R_pmrB/1-367    QGDATLLRML LRNLVENAHR YSPQGSNIMI KLQEDGGAVM AVEDEGPGID
707671-17_00428_R_pmrB/1-367    QGDATLLRML LRNLVENAHR YSPQGSNIMI KLQEDDGAVM AVEDEGPGID
719645-16_03122_R_pmrB/1-367    QGDATLLRML LRNLVENAHR YSPQGSNIMI KLQEDGGAVM AVEDEGPGID
709006-16_02800_R_pmrB/1-367    QGDATLLRML LRNLVENAHR YSPQGSNIMI KLQEDGGAVM AVEDEGPGID
721296-16_02904_R_pmrB/1-367    QGDATLLRML LRNLVENAHR YSPQGSNIMI KLQEDDGAVM AVEDEGPGID
```

```
                                301
108627-17_03754_S_pmrB/1-367    ESKCGELSKA FVRMDSRYGG IGLGLSIVSR ITQLHHGQFF LQNRQETSGT
19015727_03068_S_pmrB/1-367     ESKCGELSKA FVRMDSRYGG IGLGLSIVSR ITQLHHGQFF LQNRQETSGT
ATCC-25922_00125_S_pmrB/1-367   ESKCGELSKA FVRMDSRYGG IGLGLSIVSR ITQLHHGQFF LQNRQETSGT
108670-17_00866_S_pmrB/1-367    ESKCGELSKA FVRMDSRYGG IGLGLSIVSR ITQLHHGQFF LQNRQETSGT
603351-17_04082_S_pmrB/1-367    ESKCGELSKA FVRMDSRYGG IGLGLSIVSR ITQLHHGQFF LQNRQETSGT
703629-17_00411_S_pmrB/1-367    ESKCGELSKA FVRMDSRYGG IGLGLSIVSR ITQLHHGQFF LQNRQETSGT
703692-17_03305_S_pmrB/1-367    ESKCGELSKA FVRMDSRYGG IGLGLSIVSR ITQLHHGQFF LQNRQETSGT
703694-17_00941_S_pmrB/1-367    ESKCGELSKA FVRMDSRYGG IGLGLSIVSR ITQLHHGQFF LQNRQETSGT
705882-17_00429_S_pmrB/1-367    ESKCGELSKA FVRMDSRYGG IGLGLSIVSR ITQLHHGQFF LQNRQETSGT
705889-17_02710_S_pmrB/1-367    ESKCGELSKA FVRMDSRYGG IGLGLSIVSR ITQLHQGQFF LQNRQETSGT
722306-16_01583_S_pmrB/1-367    ESKCGELSKA FVRMDSRYGG IGLGLSIVSR ITQLHHGQFF LQNRQETSGT
14929959_02417_S_pmrB/1-367     ESKCGELSKA FVRMDSRFGG IGLGLSIVSR ITQLHHGQFF LQNRQETSGT
705801-17_01828_S_pmrB/1-367    ESKCGELSKA FVRMDSRYGG IGLGLSIVSR ITQLHHGQFF LQNRQETSGT
KP-37_03331_R_pmrB/1-367        ESKCGELSKA FVRMDSRYGG IGLGLSIVSR ITQLHHGQFF LQNRQETSGT
NCTC-13846_00784_R_pmrB/1-367   ESKCGELSKA FVRMDSRYGG IGLGLSIVSR ITQLHHGQFF LQNRQETSGT
700099-17_03188_R_pmrB/1-367    ESKCGELSKA FVRMDSRYGG IGLGLSIVSR ITQLHHGQFF LQNRQETSGT
700455-17_00209_R_pmrB/1-367    ESKCGELSKA FVRMDSRYGG IGLGLSIVSR ITQLHHGQFF LQNRQETSGT
705498-12_03777_R_pmrB/1-367    ESKCGELSKA FVRMDSRYGG IGLGLSIVSR ITQLHHGQFF LQNRQETSGT
706090-16_01485_R_pmrB/1-367    ESKCGELSKA FVRMDSRYGG IGLGLSIVSR ITQLHHGQFF LQNRQETSGT
707671-17_00428_R_pmrB/1-367    ESKCGELSKA FVRMDSRYGG IGLGLSIVSR ITQLHHGQFF LQNRQETSGT
719645-16_03122_R_pmrB/1-367    ESKCGELSKA FVRMDSRYGG IGLGLSIVSR ITQLHHGQFF LQNRQETSGT
709006-16_02800_R_pmrB/1-367    ESKCGELSKA FVRMDSRYGG IGLGLSIVSR ITQLHHGQFF LQNRQETSGT
721296-16_02904_R_pmrB/1-367    ESKCGELSKA FVRMDSRYGG IGLGLSIVSR ITQLHHGQFF LQNRQETSGT

                                351
108627-17_03754_S_pmrB/1-367    RAWIRLKKDQ YVANQI*
19015727_03068_S_pmrB/1-367     RAWVRLKKDQ NVANQI*
ATCC-25922_00125_S_pmrB/1-367   RAWIRLKKDQ YVANQI*
108670-17_00866_S_pmrB/1-367    RAWIRLKKDQ YVANQI*
603351-17_04082_S_pmrB/1-367    RAWIRLKKDQ YVANQI*
703629-17_00411_S_pmrB/1-367    RAWIRLKKDQ YVANQI*
703692-17_03305_S_pmrB/1-367    RAWIRLKKDQ YVANQI*
703694-17_00941_S_pmrB/1-367    RAWVRLKKDQ NVANQI*
705882-17_00429_S_pmrB/1-367    RAWVRLKKDQ YVANQI*
705889-17_02710_S_pmrB/1-367    RAWVRLKKDQ YVANQI*
722306-16_01583_S_pmrB/1-367    RAWVRLKKDQ YVVNQI*
14929959_02417_S_pmrB/1-367     RAWVRLKKDQ YVANQI*
705801-17_01828_S_pmrB/1-367    RAWVRLKKDQ YVANQI*
KP-37_03331_R_pmrB/1-367        RAWVRLKKDQ YVANQI*
NCTC-13846_00784_R_pmrB/1-367   RAWVRLKKDQ YVANQI*
700099-17_03188_R_pmrB/1-367    RAWVRLKKDQ NVANQI*
700455-17_00209_R_pmrB/1-367    RAWIRLKKDQ YVANQI*
705498-12_03777_R_pmrB/1-367    RAWIRLKKDQ YVANQI*
706090-16_01485_R_pmrB/1-367    RAWIRLKKDQ YVANQI*
707671-17_00428_R_pmrB/1-367    RAWVRLKKDQ YVANQI*
719645-16_03122_R_pmrB/1-367    RAWVRLKKDQ NVANQI*
709006-16_02800_R_pmrB/1-367    RAWIRLKKDQ YVANQI*
721296-16_02904_R_pmrB/1-367    RAWVRLKKDQ YVANQI*
```

```
Alignment: D:\Documentos BASILEA\AMR LAB\Projects\Colistin resistance project\Genes\E. coli gen
Seaview [blocks=10 fontsize=10 A4] on Fri Aug 14 09:40:39 2020

                                 1
108627-17_03752_S_pmrC/1-548     MLKRLLKRPS LNLFSWLLLA TFYISVCLNI AFFKQVLQVL PLDSLHNVLV
14929959_02415_S_pmrC/1-548      MLKRLLKRPS LNLLAWLLLA AFYISICLNI AFFKQVLQAL PLDSLHNVLV
19015727_03070_S_pmrC/1-548      MLKRLLKRPS LNLLAWLLLA AFYISICLNI AFFKQVLQAL PLDSLHNVLV
ATCC-25922_00127_S_pmrC/1-548    MLKRLLKRPS LNLFSWLLLA TFYISVCLNI AFFKQVLQVL PLDSLHNVLV
603351-17_04080_S_pmrC/1-548     MLKRLLKRPS LNLLAWLLLA AFYISICLNI AFFKQVLQAL PLDSLHNVLV
108670-17_00864_S_pmrC/1-548     MLKRLLKRPS LNLFSWLLLA TFYISVCLNI AFFKQVLQVL PLDSLHNVLV
703692-17_03307_S_pmrC/1-548     MLKRLLKRPS LNLFSWLLLA TFYISVCLNI AFFKQVLQVL PLDSLHNVLV
703629-17_00413_S_pmrC/1-548     MLKRLLKRPS LNLFSWLLLA TFYISVCLNI AFFKQVLQVL PLDSLHNVLV
703694-17_00939_S_pmrC/1-548     MLKRLLKRPS LNLLAWLLLA AFYISICLNI AFFKQVLQAL PLDSLHNVLV
705889-17_02712_S_pmrC/1-548     MLKRLLKRPS LNLLAWLLLA AFYISICLNI AFFKQVLQAL PLDSLHNVLV
722306-16_01585_S_pmrC/1-548     MLKRLLKRPS LNLLAWLLLA AFYISICLNI AFFKQVLQAL PLDSLHNVLV
705882-17_00427_S_pmrC/1-548     MLKRLLKRPS LNLLAWLLLA AFYISICLNI AFFKQVLQAL PLDSLHNVLV
705801-17_01830_S_pmrC/1-548     MLKRLLKRPS LNLLAWLLLA AFYISICLNI AFFKQVLQAL PLDSLHNVLV
KP-37_03329_R_pmrC/1-548         MLKRLLKRPS LNLLAWLLLA AFYISIYLNI AFFKQVLQAL PLDSLHNVLV
NCTC-13846_00782_R_pmrC/1-548    MLKRLLKRPS LNLLAWLLLA AFYISICLNI AFFKQVLQAL PLDSLHNVLV
707671-17_00426_R_pmrC/1-548     MLKRLLKRPS LNLLAWLLLA AFYISIYLNI AFFKQVLQAL PLDSLHNVLV
700099-17_03186_R_pmrC/1-548     MLKRLLKRPS LNLLAWLLLA AFYISICLNI AFFKQVLQAL PLDSLHNVLV
700455-17_00207_R_pmrC/1-548     MLKRLLKRPS LDLFSWLLLA TFYISVCLNI AFFKQVLQVL PLDSLHNVLV
709006-16__R_pmrC/1-548          MLKRLLKRPS LNLFSWLLLA TFYISVCLNI AFFKQVLQVL PLDSLHNVLV
705498-12_03775_R_pmrC/1-548     MLKRLLKRPS LNLFSWLLLA TFYISVCLNI AFFKQVLQVL PLDSLHNVLV
706090-16_01483_R_pmrC/1-548     MLKRLLKRPS LNLFSWLLLA TFYISVCLNI AFFKQVLQVL PLDSLHNVLV
719645-16_03124_R_pmrC/1-548     MLKRLLKRPS LNLLAWLLLA AFYISICLNI AFFKQVLQAL PLDSLHNVLV
721296-16_02906_R_pmrC/1-548     MLKRLLKRPS LNLLAWLLLA AFYISIYLNI AFFKQVLQAL PLDSLHNVLV

                                51
108627-17_03752_S_pmrC/1-548     FLSMPVVAFS VINIVLTLGS FLWLNRPLAC LFILVGAAAQ YFIMTYGIVI
14929959_02415_S_pmrC/1-548      FLSMPVVAFS VINIVLTLSS FLWLNRPLAC LFILVGAAAQ YFIMTYGIVI
19015727_03070_S_pmrC/1-548      FLSMPVVAFS VINIVLTLSS FLWLNRPLAC LFILVGAAAQ YFIMTYGIVI
ATCC-25922_00127_S_pmrC/1-548    FLSMPVVAFS VINIVLTLGS FLWLNRPLAC LFILVGAAAQ YFIMTYGIVI
603351-17_04080_S_pmrC/1-548     FLSMPVVAFS VINIVLTLSS FLWLNRPLAC LFILVGAAAQ YFIMTYGIVI
108670-17_00864_S_pmrC/1-548     FLSMPVVAFS VINIVLTLGS FLWLNRPLAC LFILVGAAAQ YFIMTYGIVI
703692-17_03307_S_pmrC/1-548     FLSMPVVAFS VINIVLTLGS FLWLNRPLAC LFILVGAAAQ YFIMTYGIVI
703629-17_00413_S_pmrC/1-548     FLSMPVVAFS VINIVLTLGS FLWLNRPLAC LFILVGAAAQ YFIMTYGIVI
703694-17_00939_S_pmrC/1-548     FLSMPVVAFS VINIVLTLSS FLWLNRPLAC LFILVGAAAQ YFIMTYGIVI
705889-17_02712_S_pmrC/1-548     FLSMPVVAFS VINIVLTLSS FLWLNRPLAC LFILVGAAAQ YFIMTYGIVI
722306-16_01585_S_pmrC/1-548     FLSMPVVAFS VINIVLTLSS FLWLNRPLAC LFILVGAAAQ YFIMTYGIVI
705882-17_00427_S_pmrC/1-548     FLSMPVVAFS VINIVLTLSS FLWLNRPLAC LFILVGAAAQ YFIMTYGIVI
705801-17_01830_S_pmrC/1-548     FLSMPVVAFS VINIVLTLSS FLWLNRPLAC LFILVGAAAQ YFIMTYGIVI
KP-37_03329_R_pmrC/1-548         FLSMPVVAFS VINIVLTLSS FLWINRPLAC LFILVGAAAQ YFIMTYGIVI
NCTC-13846_00782_R_pmrC/1-548    FLSMPVVAFS VINIVLTLSS FLWLNRPLAC LFILVGAAAQ YFIMTYGIVI
707671-17_00426_R_pmrC/1-548     FLSMPVVAFS VINIVLTLSS FLWLNRPLAC LFILVGAAAQ YFIMTYGIVI
700099-17_03186_R_pmrC/1-548     FLSMPVVAFS VINIVLTLSS FLWLNRPLAC LFILVGAAAQ YFIMTYGIVI
700455-17_00207_R_pmrC/1-548     FLSMPVVAFS VINIVLTLGS FLWLNRPLAC LFILVGAAAQ YFIMTYGIVI
709006-16__R_pmrC/1-548          FLSMPVVAFS VINIVLTLGS FLWLNRPLAC LFILVGAAAQ YFIMTYGIVI
705498-12_03775_R_pmrC/1-548     FLSMPVVAFS VINIVLTLGS FLWLNRPLAC LFILVGAAAQ YFIMTYGIVI
706090-16_01483_R_pmrC/1-548     FLSMPVVAFS VINIVLTLGS FLWLNRPLAC LFILVGAAAQ YFIMTYGIVI
719645-16_03124_R_pmrC/1-548     FLSMPVVAFS VINIVLTLSS FLWLNRPLAC LFILVGAAAQ YFIMTYGIVI
721296-16_02906_R_pmrC/1-548     FLSMPVVAFS VINIVLTLSS FLWLNRPLAC LFILVGAAAQ YFIMTYGIVI

                               101
108627-17_03752_S_pmrC/1-548     DRSMITNIID TTPAESYALM TPRMLLTLGL SGVLAAIIAC WIKIKPTTSR
14929959_02415_S_pmrC/1-548      DRSMIANIID TTPAESYALM TPQMLLTLGF SGVLAALIAC WIKIKPATSR
19015727_03070_S_pmrC/1-548      DRSMIANIID TTPAESYALM TPQMLLTLGF SGVLAALIAC WIKIKPATSR
ATCC-25922_00127_S_pmrC/1-548    DRSMITNIID TTPAESYALM TPRMLLTLGL SGVLAAIIAC WIKIKPTTSR
603351-17_04080_S_pmrC/1-548     DRSMIANIID TTPAESYALM TPQMLLTLGF SGVLAALIAC WIKIKPTTSR
108670-17_00864_S_pmrC/1-548     DRSMITNIID TTPAESYALM TPRMLLTLGL SGVLAAIIAC WIKITPTTSR
703692-17_03307_S_pmrC/1-548     DRSMITNIID TTPAESYALM TPRMLLTLGL SGVLAAIIAC WIKIKPTTSR
703629-17_00413_S_pmrC/1-548     DRSMITNIID TTPAESYALM TPRMLLTLGL SGVLAAIIAC WIKIKPTTSR
703694-17_00939_S_pmrC/1-548     DRSMIANIID TTPAESYALM TPQMLLTLGF SGVLAALIAC WIKIKPATSR
705889-17_02712_S_pmrC/1-548     DRSMIANIID TTPAESYALM TPQMLLTLGF SGVLAALIAC WIKIKPATSR
722306-16_01585_S_pmrC/1-548     DRSMIANIID TTPAESYALM TPQMLLTLGF SGVLAALIAC WIKIKPASSR
705882-17_00427_S_pmrC/1-548     DRSMIANIID TTPAESYALM TPQMLLTLGF SGVLAALIAC WIKIKPATSR
705801-17_01830_S_pmrC/1-548     DRSMIANIID TTPAESYALM TPQMLLTLGF SGVLAALIAC WIKIKPTTSR
KP-37_03329_R_pmrC/1-548         DRSMIANIID TTPAESYALM TPQMLLTLGF SGVLAALIAC WIKIKPATSR
NCTC-13846_00782_R_pmrC/1-548    DRSMIANIID TTPAESYALM TPQMLLTLGF SGVLAALIAC WIKIKPAASR
707671-17_00426_R_pmrC/1-548     DRSMIANIID TTPAESYALM TPQMLLTLGF SGVLAALIAC WIKIKPATSR
700099-17_03186_R_pmrC/1-548     DRSMIANIID TTPAESYALM TPQMLLTLGF SGVLAALIAC WIKIKPATSR
700455-17_00207_R_pmrC/1-548     DRSMITNIID TTPAESYALM TPRMLLTLGL SGVLAAIIAC WIKIKPTTSR
709006-16__R_pmrC/1-548          DRSMITNIID TTPAESYALM TPRMLLTLGL SGVLAAIIAC WIKIKPTTSR
705498-12_03775_R_pmrC/1-548     DRSMITNIID TTPAESYALM TPRMLLTLGL SGVLAAIIAC WIKIKPTTSR
706090-16_01483_R_pmrC/1-548     DRSMITNIID TTPAESYALM TPRMLLTLGL SGVLAAIIAC WIKITPTTSR
719645-16_03124_R_pmrC/1-548     DRSMIANIID TTPAESYALM TPQMLLTLGF SGVLAALIAC WIKIKPATSR
721296-16_02906_R_pmrC/1-548     DRSMIANIID TTPAESYALM TPQMLLTLGF SGVLAALIAC WIKIKPATSR
```

```
                                 151
108627-17_03752_S_pmrC/1-548     LRSVLFRGAN ILISVLLILL VAALFYKDYA SLFRNNKELV KSLSPSNSIV
14929959_02415_S_pmrC/1-548      LRSVLFRGAN ILVSVLLILL VAALFYKDYA SLFRNNKELV KSLSPSNSIV
19015727_03070_S_pmrC/1-548      LRSVLFRGAN ILVSVLLILL VAALFYKDYA SLFRNNKELV KSLSPSNSIV
ATCC-25922_00127_S_pmrC/1-548    LRSVLFRGAN ILISVLLILL VAALFYKDYA SLFRNNKELV KSLSPSNSIV
603351-17_04080_S_pmrC/1-548     LRSVLFRGAN ILISVLLILL VAALFYKDYA SLFRNNKELV KSLSPSNSIV
108670-17_00864_S_pmrC/1-548     LRSVLFRGAN ILISVLLILL VAALFYKDYA SLFRNNKELV KSLSPSNSIV
703692-17_03307_S_pmrC/1-548     LRSVLFRGAN ILISVLLILL VAALFYKDYA SLFRNNKELV KSLSPSNSIV
703629-17_00413_S_pmrC/1-548     LRSVLFRGAN ILISVLLILL VAALFYKDYA SLFRNNKELV KSLSPSNSIV
703694-17_00939_S_pmrC/1-548     LRSVLFRGAN ILVSVLLILL VAALFYKDYA SLFRNNKELV KSLSPSNSIV
705889-17_02712_S_pmrC/1-548     LRSVLFRGAN ILVSVLLILL VAALFYKDYA SLFRNNKELV KSLSPSNSIV
722306-16_01585_S_pmrC/1-548     LRSVLFRGAN ILVSVLLILL VAALFYKDYA SLFRNNKELV KSLSPSNSIV
705882-17_00427_S_pmrC/1-548     LRSVLFRGAN ILVSVLLILL VAALFYKDYA SLFRNNKELV KSLSPSNSIV
705801-17_01830_S_pmrC/1-548     LRSVLFRGAN ILVSVLLILL VAALFYKDYA SLFRNNKELV KSLSPSNSIV
KP-37_03329_R_pmrC/1-548         LRSVLFRGAN ILVSVLLILL VAALFYKDYA SLFRNNKELV KSLSPSNSIV
NCTC-13846_00782_R_pmrC/1-548    LRSVLFRGAN ILVSVLLILL VAALFYKDYA SLFRNNKELV KSLSPSNSIV
707671-17_00426_R_pmrC/1-548     LRSVLFRGAN ILVSVLLILL VAALFYKDYA SLFRNNKELV KSLSPSNSIV
700099-17_03186_R_pmrC/1-548     LRSVLFRGAN ILVSVLLILL VAALFYKDYA SLFRNNKELV KSLSPSNSIV
700455-17_00207_R_pmrC/1-548     LRSVLFRGAN ILISVLLILL VAALFYKDYA SLFRNNKELV KSLSPSNSIV
709006-16__R_pmrC/1-548          LRSVLFRGAN ILISVLLILL VAALFYKDYA SLFRNNKELV KSLSPSNSIV
705498-12_03775_R_pmrC/1-548     LRSVLFRGAN ILISVLLILL VAALFYKDYA SLFRNNKELV KSLSPSNSIV
706090-16_01483_R_pmrC/1-548     LRSVLFRGAN ILISVLLILL VAALFYKDYA SLFRNNKELV KSLSPSNSIV
719645-16_03124_R_pmrC/1-548     LRSVLFRGAN ILVSVLLILL VAALFYKDYA SLFRNNKELV KSLSPSNSIV
721296-16_02906_R_pmrC/1-548     LRSVLFRGAN ILVSVLLILL VAALFYKDYA SLFRNNKELV KSLSPSNSIV

                                 201
108627-17_03752_S_pmrC/1-548     ASWSWYSHQR SANLPLIRIG EDAHRNPLMQ NGKRKNLTIL IVGETSRAEN
14929959_02415_S_pmrC/1-548      ASWSWYSHQR LANLPLVRIG EDAHRNPLMQ NETRKNLTIL IVGETSRAEN
19015727_03070_S_pmrC/1-548      ASWSWYSHQR LANLPLVRIG EDAHRNPLMQ NEKRKNLTIL IVGETSRAEN
ATCC-25922_00127_S_pmrC/1-548    ASWSWYSHQR SANLPLIRIG EDAHRNPLMQ NGKRKNLTIL IVGETSRAEN
603351-17_04080_S_pmrC/1-548     ASWSWYSHQR SANLPLIRIG EDAHRNPLMQ NGKRKNLTIL IVGETSRAEN
108670-17_00864_S_pmrC/1-548     ASWSWYSHQR SANLPLIRIG EDAHRNPLMQ NGKRKNLTIL IVGETSRAEN
703692-17_03307_S_pmrC/1-548     ASWSWYSHQR SANLPLIRIG EDAHRNPLMQ NGKRKNLTIL IVGETSRAEN
703629-17_00413_S_pmrC/1-548     ASWSWYSHQR SANLPLIRIG EDAHRNPLMQ NGKRKNLTIL IVGETSRAEN
703694-17_00939_S_pmrC/1-548     ASWSWYSHQR LANLPLVRIG EDAHRNPLMQ NEKRKNLTIL IVGETSRAEN
705889-17_02712_S_pmrC/1-548     ASWSWYSHQR LANLPLVRIG EDAHRNPLMQ NEKRKNLTIL IVGETSRAEN
722306-16_01585_S_pmrC/1-548     ASWSWYSHQR LANLPLVRIG EDAHRNPLMQ NEKRKNLTIL IVGETSRAEN
705882-17_00427_S_pmrC/1-548     ASWSWYSHQR LANLPLVRIG EDAHRNPLMQ NETRKNLTIL IVGETSRAEN
705801-17_01830_S_pmrC/1-548     ASWSWYSHQR LANLPLVRIG EDAHRNPLMQ NEKRKNLTIL IVGETSRAEN
KP-37_03329_R_pmrC/1-548         ASWSWYSHQR LANLPLVRIG EDAHRNPLMQ NEKRKNLTIL IVGETSRAEN
NCTC-13846_00782_R_pmrC/1-548    ASWSWYSHQR LANLPLVRIG EDAHRNPLMQ NEKRKNLTIL IVGETSRAEN
707671-17_00426_R_pmrC/1-548     ASWSWYSHQR LANLPLVRIG EDAHRNPLMQ NEKRKNLTIL IVGETSRAEN
700099-17_03186_R_pmrC/1-548     ASWSWYSHQR LANLPLVRIG EDAHRNPLMQ NEKRKNLTIL IVGETSRAEN
700455-17_00207_R_pmrC/1-548     ASWSWYSHQR SANLPLIRIG EDAHRNPLMQ NGKRKNLTIL IVGETSRAEN
709006-16__R_pmrC/1-548          ASWSWYSHQR SANLPLIRIG EDAHRNPLMQ NGKRKNLTIL IVGETSRAEN
705498-12_03775_R_pmrC/1-548     ASWSWYSHQR SANLPLIRIG EDAHRNPLMQ NGKRKNLTIL IVGETSRAEN
706090-16_01483_R_pmrC/1-548     ASWSWYSHQR SANLPLIRIG EDAHRNPLMQ NGKRKNLTIL IVGETSRAEN
719645-16_03124_R_pmrC/1-548     ASWSWYSHQR LANLPLVRIG EDAHRNPLMQ NEKRKNLTIL IVGETSRAEN
721296-16_02906_R_pmrC/1-548     ASWSWYSHQR LANLPLVRIG EDAHRNPLMQ NEKRKNLTIL IVGETSRAEN

                                 251
108627-17_03752_S_pmrC/1-548     FSLNGYQRET NPRLAKDNVV YFPNTASCGT ATAVSVPCMF SDMPREHYKE
14929959_02415_S_pmrC/1-548      FSLNGYPRET NPRLAKDNVV YFPNTASCGT ATAVSVPCMF SDMPREHYKE
19015727_03070_S_pmrC/1-548      FSLNGYPRET NPRLAKDNVV YFPNTASCGT ATAVSVPCMF SDMPREHYKE
ATCC-25922_00127_S_pmrC/1-548    FSLNGYQRET NPRLAKDNVV YFPNTASCGT ATAVSVPCMF SDMPREHYKE
603351-17_04080_S_pmrC/1-548     FSLNGYPRET NPRLAKDNVV YFPNTASCGT ATAVSVPCMF SDMPREHYKE
108670-17_00864_S_pmrC/1-548     FSLNGYPRET NPRLAKDNVV YFPNTASCGT ATAVSVPCMF SDMPREHYKE
703692-17_03307_S_pmrC/1-548     FSLNGYQRET NPRLAKDNVV YFPNTASCGT ATAVSVPCMF SDMPREHYKE
703629-17_00413_S_pmrC/1-548     FSLNGYPRET NPRLAKDNVV YFPNTASCGT ATAVSVPCMF SDMPREHYKE
703694-17_00939_S_pmrC/1-548     FSLNGYPRET NPRLAKDNVV YFPNTASCGT ATAVSVPCMF SDMPREHYKE
705889-17_02712_S_pmrC/1-548     FSLNGYPRET NPRLAKDNVV YFPNTASCGT ATAVSVPCMF SDMPREHYKE
722306-16_01585_S_pmrC/1-548     FSLNGYPRET NPRLAKDNVV YFPNTASCGT ATAVSVPCMF SDMPREHYKE
705882-17_00427_S_pmrC/1-548     FSLNGYPRET NPRLAKDNVV YFPNTASCGT ATAVSVPCMF SDMPREHYKE
705801-17_01830_S_pmrC/1-548     FSLNGYPRET NPRLAKDNVV YFPNTASCGT ATAVSVPCMF SDMPREHYKE
KP-37_03329_R_pmrC/1-548         FSLNGYPRET NPRLAKDNVV YFPNTASCGT ATAVSVPCMF SDMPREHYKE
NCTC-13846_00782_R_pmrC/1-548    FSLNGYPRET NPRLAKDNVV YFPNTASCGT ATAVSVPCMF SDMPREHYKE
707671-17_00426_R_pmrC/1-548     FSLNGYPRET NPRLAKDNVV YFPNTASCGT ATAVSVPCMF SDMPREHYKE
700099-17_03186_R_pmrC/1-548     FSLNGYPRET NPRLAKDNVV YFPNTASCGT ATAVSVPCMF SDMPREHYKE
700455-17_00207_R_pmrC/1-548     FSLNGYPRET NPRLAKDNVV YFPNTASCGT ATAVSVPCMF SDMPREHYKE
709006-16__R_pmrC/1-548          FSLNGYPRET NPRLAKDNVV YFPNTASCGT ATAVSVPCMF SDMPREHYKE
705498-12_03775_R_pmrC/1-548     FSLNGYPRET NPRLAKDNVV YFPNTASCGT ATAVSVPCMF SDMPREHYKE
706090-16_01483_R_pmrC/1-548     FSLNGYPRET NPRLAKDNVV YFPNTASCGT ATAVSVPCMF SDMPREHYKE
719645-16_03124_R_pmrC/1-548     FSLNGYPRET NPRLAKDNVV YFPNTASCGT ATAVSVPCMF SDMPREHYKE
721296-16_02906_R_pmrC/1-548     FSLNGYPRET NPRLAKDNVV YFPNTASCGT ATAVSVPCMF SDMPREHYKE
```

```
                                   301
108627-17_03752_S_pmrC/1-548       ELAQHQEGVL DIIQRAGINV LWNDNDGGCK GVCDRVPHQN ITALNLPGQC
14929959_02415_S_pmrC/1-548        ELAQHQEGVL DIIQRAGINV LWNDNDGGCK GACDRVPHQN VTALNLPGQC
19015727_03070_S_pmrC/1-548        ELAQHQEGVL DIIQRAGINV LWNDNDGGCK GACDRVPHQN VTALNLPGQC
ATCC-25922_00127_S_pmrC/1-548      ELAQHQEGVL DIIQRAGINV LWNDNDGGCK GVCDRVPHQN ITALNLPGQC
603351-17_04080_S_pmrC/1-548       ELAQHQEGVL DIIQRAGINV LWNDNDGGCK GVCDRVPHQN VTALNLPGQC
108670-17_00864_S_pmrC/1-548       ELAQHQEGVL DIIQRAGINV LWNDNDGGCK GVCDRVPHQN ITALNLPGQC
703692-17_03307_S_pmrC/1-548       ELAQHQEGVL DIIQRAGINV LWNDNDGGCK GVCDRVPHQN ITALNLPGQC
703629-17_00413_S_pmrC/1-548       ELAQHQEGVL DIIQRAGINV LWNDNDGGCK GVCDRVPHQN VTALNLPGQC
703694-17_00939_S_pmrC/1-548       ELAQHQEGVL DIIQRAGINV LWNDNDGGCK GACDRVPHQN VTALNLPGQC
705889-17_02712_S_pmrC/1-548       ELAQHQEGVL DIIQRAGINV LWNDNDGGCK GACDRVPHQN VTALNLPDQC
722306-16_01585_S_pmrC/1-548       ELAQHQEGVL DIIQRAGINV LWNDNDGGCK GVCDRVPHQN VTALNLPGQC
705882-17_00427_S_pmrC/1-548       ELAQHQEGVL DIIQRAGINV LWNDNDGGCK GACDRVPHQN VTALNLPGQC
705801-17_01830_S_pmrC/1-548       ELAQHQEGVL DIIQRAGINV LWNDNDGGCK GACDRVPHQN VTALNLPGQC
KP-37_03329_R_pmrC/1-548           ELAQHQEGVL DIIQRAGINV LWNDNDGGCK GACDRVPHQN VTALNLPDQC
NCTC-13846_00782_R_pmrC/1-548      ELAQHQEGVL DIIQRAGINV LWNDNDGGCK GVCDRVPHQN VTALNLPGQC
707671-17_00426_R_pmrC/1-548       ELAQHQEGVL DIIQRAGINV LWNDNDGGCK GACDRVPHQN VTALNLPDQC
700099-17_03186_R_pmrC/1-548       ELAQHQEGVL DIIQRAGINV LWNDNDGGCK GACDRVPHQN VTALNLPGQC
700455-17_00207_R_pmrC/1-548       ELAQHQEGVL DIIQRAGINV LWNDNDGGCK GVCDRVPHQN VTALNLPGQC
709006-16__R_pmrC/1-548            ELAQHQEGVL DIIQRAGINV LWNDNDGGCK GVCDRVPHQN VTALNLPGQC
705498-12_03775_R_pmrC/1-548       ELAQHQEGVL DIIQRAGINV LWNDNDGGCK GVCDRVPHQN VTALNLPGQC
706090-16_01483_R_pmrC/1-548       ELAQHQEGVL DIIQRAGINV LWNDNDGGCK GVCDRVPHQN ITALNLPGQC
719645-16_03124_R_pmrC/1-548       ELAQHQEGVL DIIQRAGINV LWNDNDGGCK GACDRVPHQN VTALNLPGQC
721296-16_02906_R_pmrC/1-548       ELAQHQEGVL DIIQRAGINV LWNDNDGGCK GACDRVPHQN VTALNLPDQC

                                   351
108627-17_03752_S_pmrC/1-548       INGECYDEVL FHGLEEYINN LQSDGLIVLH TIGSHGPTYY NRYPPQFRKF
14929959_02415_S_pmrC/1-548        INGECYDEVL FHGLEEYINN LQGDGVIVLH TIGSHGPTYY NRYPPQFRKF
19015727_03070_S_pmrC/1-548        INGECYDEVL FHGLEEYINN LQGDGVIVLH TIGSHGPTYY NRYPPQFRKF
ATCC-25922_00127_S_pmrC/1-548      INGECYDEVL FHGLEEYINN LQSDGLIVLH TIGSHGPTYY NRYPPQFRKF
603351-17_04080_S_pmrC/1-548       INGECYDEVL FHGLEEYINN LQSDGLIVLH TIGSHGPTYY NRYPPQFRKF
108670-17_00864_S_pmrC/1-548       INGECYDEVL FHGLEEYINN LQSDGLIVLH TIGSHGPTYY NRYPPQFRKF
703692-17_03307_S_pmrC/1-548       INGECYDEVL FHGLEEYINN LQSDGLIVLH TIGSHGPTYY NRYPPQFRKF
703629-17_00413_S_pmrC/1-548       INGECYDEVL FHGLEDYINN LQGDGVIVLH TIGSHGPTYY NRYPPQFRKF
703694-17_00939_S_pmrC/1-548       INGECYDEVL FHGLEEYINN LQGDGVIVLH TIGSHGPTYY NRYPPQFRKF
705889-17_02712_S_pmrC/1-548       INGECYDEVL FHGLEEYINN LQGDGVIVLH TIGSHGPTYY NRYPPQFRKF
722306-16_01585_S_pmrC/1-548       INGECYDEVL FHGLEDYINN LQGDGVIVLH TIGSHGPTYY NRYPPQFRKF
705882-17_00427_S_pmrC/1-548       INGECYDEVL FHGLEEYINN LQGDGVIVLH TIGSHGPTYY NRYPPQFRKF
705801-17_01830_S_pmrC/1-548       INGECYDEVL FHGLEEYINN LQGDGVIVLH TIGSHGPTYY NRYPPQFRKF
KP-37_03329_R_pmrC/1-548           INGECYDEVL FHGLEEYINN LQGDGVIVLH TIGSHGPTYY NRYPPQFRKF
NCTC-13846_00782_R_pmrC/1-548      INGECYDEVL FHGLEDYINN LQGDGVIVLH TIGSHGPTYY NRYPPQFRKF
707671-17_00426_R_pmrC/1-548       INGECYDEVL FHGLEEYINN LQGDGVIVLH TIGSHGPTYY NRYPPQFRKF
700099-17_03186_R_pmrC/1-548       INGECYDEVL FHGLEEYINN LQGDGVIVLH TIGSHGPTYY NRYPPQFRKF
700455-17_00207_R_pmrC/1-548       INGECYDEVL FHGLEDYINN LQGDGVIVLH TIGSHGPTYY NRYPPQFRKF
709006-16__R_pmrC/1-548            INGECYDEVL FHGLEDYINN LQGDGVIVLH TIGSHGPTYY NRYPPQFRKF
705498-12_03775_R_pmrC/1-548       INGECYDEVL FHGLEDYINN LQGDGVIVLH TIGSHGPTYY NRYPPQFRKF
706090-16_01483_R_pmrC/1-548       INGECYDEVL FHGLEDYINN LQGDGVIVLH TIGSHGPTYY NRYPPQFRKF
719645-16_03124_R_pmrC/1-548       INGECYDEVL FHGLEEYINN LQGDGVIVLH TIGSHGPTYY NRYPPQFRKF
721296-16_02906_R_pmrC/1-548       INGECYDEVL FHGLEEYINN LQGDGVIVLH TIGSHGPTYY NRYPPQFRKF

                                   401
108627-17_03752_S_pmrC/1-548       TPTCDTNEIQ TCTKEQLVNT YDNTLVYVDY IVDKAINLLK EHQDKFTTSL
14929959_02415_S_pmrC/1-548        TPTCDTNEIQ TCTKEQLVNT YDNTLVYVDY IVDKAINLLK EHQDKFTTSL
19015727_03070_S_pmrC/1-548        TPTCDTNEIQ TCSKEQLVNT YDNTLVYVDY IVDKAINLLK EHQDKFTTSL
ATCC-25922_00127_S_pmrC/1-548      TPTCDTNEIQ TCTKEQLVNT YDNTLVYVDY IVDKAINLLK EHQDKFTTSL
603351-17_04080_S_pmrC/1-548       TPTCDTNEIQ TCTQEQLVNT YDNTLVYVDY IVDKAINLLK EHQDKFTTSL
108670-17_00864_S_pmrC/1-548       TPTCDTNEIQ TCTQEQLVNT YDNTLVYVDY IVDKAINLLK EHQDKFTTSL
703692-17_03307_S_pmrC/1-548       TPTCDTNEIQ TCTKEQLVNT YDNTLVYVDY IVDKAINLLK EHQDKFTTSL
703629-17_00413_S_pmrC/1-548       TPTCDTNEIQ TCTQEQLVNT YDNTLVYVDY IVDKAINLLK EHQDKFTTSL
703694-17_00939_S_pmrC/1-548       TPTCDTNEIQ TCSKEQLVNT YDNTLVYVDY IVDKAINLLK EHQDKFTTSL
705889-17_02712_S_pmrC/1-548       TPTCDTNEIQ TCTKEQLVNT YDNTLVYVDY IVDKAINLLK EHQDKFTTSL
722306-16_01585_S_pmrC/1-548       TPTCDTNEIQ TCTQEQLVNT YDNTLVYVDY IVDKAINLLK EHQDKFTTSL
705882-17_00427_S_pmrC/1-548       TPTCDTNEIQ TCTKEQLVNT YDNTLVYVDY IVDKAINLLK EHQDKFTTSL
705801-17_01830_S_pmrC/1-548       TPTCDTNEIQ TCTKEQLVNT YDNTLVYVDY IVDKAINLLK EHQDKFTTSL
KP-37_03329_R_pmrC/1-548           TPTCDTNEIQ TCTKEQLVNT YDNTLVYVDY IVDKAINLLK EHQDKFTTSL
NCTC-13846_00782_R_pmrC/1-548      TPTCDTNEIQ TCTQEQLVNT YDNTLVYVDY IVDKAINLLK EHQDKFTTSL
707671-17_00426_R_pmrC/1-548       TPTCDTNEIQ TCTKEQLVNT YDNTLVYVDY IVDKAINLLK EHQDKFTTSL
700099-17_03186_R_pmrC/1-548       TPTCDTNEIQ TCSKEQLVNT YDNTLVYVDY IVDKAINLLK EHQDKFTTSL
700455-17_00207_R_pmrC/1-548       TPTCDTNEIQ TCTQEQLVNT YDNTLVYVDY IVDKAINLLK EHQDKFTTSL
709006-16__R_pmrC/1-548            TPTCDTNEIQ TCTQEQLVNT YDNTLVYVDY IVDKAINLLK EHQDKFTTSL
705498-12_03775_R_pmrC/1-548       TPTCDTNEIQ TCTQEQLVNT YDNTLVYVDY IVDKAINLLK EHQDKFTTSL
706090-16_01483_R_pmrC/1-548       TPTCDTNEIQ TCTKEQLVNT YDNTLVYVDY IVDKAINLLK EHQDKFTTSL
719645-16_03124_R_pmrC/1-548       TPTCDTNEIQ TCSKEQLVNT YDNTLVYVDY IVDKAINLLK EHQDKFTTSL
721296-16_02906_R_pmrC/1-548       TPTCDTNEIQ TCTKEQLVNT YDNTLVYVDY IVDKAINLLK EHQDKFTTSL
```

```
                                451
108627-17_03752_S_pmrC/1-548    VYLSDHGESL GENGIYLHGL PYAIAPDSQK QVPMLLWLSE DYQKRYQVDQ
14929959_02415_S_pmrC/1-548     VYLSDHGESL GENGIYLHGL PYAIAPDSQK QVPMLLWLSE DYQKRYQVDQ
19015727_03070_S_pmrC/1-548     VYLSDHGESL GENGIYLHGL PYAIAPDSQK QVPMLLWLSE DYQKRYQVDQ
ATCC-25922_00127_S_pmrC/1-548   VYLSDHGESL GENGIYLHGL PYAIAPDSQK QVPMLLWLSE DYQKRYQVDQ
603351-17_04080_S_pmrC/1-548    VYLSDHGESL GENGIYLHGL PYAIAPDSQK QVPMLLWLSE DYQKRYQVDQ
108670-17_00864_S_pmrC/1-548    VYLSDHGESL GENGIYLHGL PYAIAPDSQK QVPMLLWLSE DYQKRYQVDQ
703692-17_03307_S_pmrC/1-548    VYLSDHGESL GENGIYLHGL PYAIAPDSQK QVPMLLWLSE DYQKRYQVDQ
703629-17_00413_S_pmrC/1-548    VYLSDHGESL GENGIYLHGL PYAIAPDSQK QVPMLLWLSE DYQKRYQVDQ
703694-17_00939_S_pmrC/1-548    VYLSDHGESL GENGIYLHGL PYAIAPDSQK QVPMLLWLSE DYQKRYQVDQ
705889-17_02712_S_pmrC/1-548    VYLSDHGESL GENGIYLHGL PYAIAPDSQK QVPMLLWLSE DYQKRYQVDQ
722306-16_01585_S_pmrC/1-548    VYLSDHGESL GENGIYLHGL PYAIAPDSQK QVPMLLWLSE DYQKRYQVDQ
705882-17_00427_S_pmrC/1-548    VYLSDHGESL GENGIYLHGL PYAIAPDSQK QVPMLLWLSE DYQKRYQVDQ
705801-17_01830_S_pmrC/1-548    VYLSDHGESL GENGIYLHGL PYAIAPDSQK QVPMLLWLSE DYQKRYQVDQ
KP-37_03329_R_pmrC/1-548        VYLSDHGESL GENGIYLHGL PYAIAPDSQK QVPMLLWLSE DYQKRYQVDQ
NCTC-13846_00782_R_pmrC/1-548   VYLSDHGESL GENGIYLHGL PYAIAPDSQK QVPMLLWLSE DYQKRYQVDQ
707671-17_00426_R_pmrC/1-548    VYLSDHGESL GENGIYLHGL PYAIAPDSQK QVPMLLWLSE DYQKRYQVDQ
700099-17_03186_R_pmrC/1-548    VYLSDHGESL GENGIYLHGL PYAIAPDSQK QVPMLLWLSE DYQKRYQVDQ
700455-17_00207_R_pmrC/1-548    VYLSDHGESL GENGIYLHGL PYAIAPDSQK QVPMLLWLSE DYQKRYQVDQ
709006-16__R_pmrC/1-548         VYLSDHGESL GENGIYLHGL PYAIAPDSQK QVPMLLWLSE DYQKRYQVDQ
705498-12_03775_R_pmrC/1-548    VYLSDHGESL GENGIYLHGL PYAIAPDSQK QVPMLLWLSE DYQKRYQVDQ
706090-16_01483_R_pmrC/1-548    VYLSDHGESL GENGIYLHGL PYAIAPDSEK QVPMLLWLSE DYQKRYQVDQ
719645-16_03124_R_pmrC/1-548    VYLSDHGESL GENGIYLHGL PYAIAPDSQK QVPMLLWLSE DYQKRYQVDQ
721296-16_02906_R_pmrC/1-548    VYLSDHGESL GENGIYLHGL PYAIAPDSQK QVPMLLWLSE DYQKRYQVDQ

                                501
108627-17_03752_S_pmrC/1-548    NCLQKQAQTQ HYSQDNLFST LLGLTGVETK YYQAADDILQ TCRRVSE*
14929959_02415_S_pmrC/1-548     NCLQKQAQTQ HYSQDNLFST LLGLTGVETK YYQAADDILQ TCRRVSE*
19015727_03070_S_pmrC/1-548     NCLQKQAQTQ HYSQDNLFST LLGLTGVETK YYQAADDILQ TCRRVSE*
ATCC-25922_00127_S_pmrC/1-548   NCLQKQAQTQ HYSQDNLFST LLGLTGVETK YYQAADDILQ TCRRVSE*
603351-17_04080_S_pmrC/1-548    NCLQKQAQTQ HYSQDNLFST LLGLTGVETK YYQAADDILQ TCRRVSE*
108670-17_00864_S_pmrC/1-548    NCLQKQAQTQ HYSQDNLFST LLGLTGVETK YYQAADDILQ TCRRVSE*
703692-17_03307_S_pmrC/1-548    NCLQKQAQTQ HYSQDNLFST LLGLTGVETK YYQAADDILQ TCRRVSE*
703629-17_00413_S_pmrC/1-548    NCLQKQAQTQ HYSQDNLFST LLGLTGVETK YYQAADDILQ TCRRVSE*
703694-17_00939_S_pmrC/1-548    NCLQKQAQTQ HYSQDNLFST LLGLTGVETK YYQAADDILQ TCRRVSE*
705889-17_02712_S_pmrC/1-548    NCLQKQAQTQ HYSQDNLFST LLGLTGVETK YYQAADDILQ TCRRVSE*
722306-16_01585_S_pmrC/1-548    NCLQKQAQTQ HYSQDNLFST LLGLTGVETK YYQAADDILQ TCRRVSE*
705882-17_00427_S_pmrC/1-548    NCLQKQAQTQ HYSQDNLFST LLGLTGVETK YYQAADDILQ TCRRVSE*
705801-17_01830_S_pmrC/1-548    NCLQKQAQTQ HYSQDNLFST LLGLTGVETK YYQAADDILQ TCRRVSE*
KP-37_03329_R_pmrC/1-548        NCLQKQAQTQ HYSQDNLFST LLGLTGVETK YYQAADDILQ TCRRVSE*
NCTC-13846_00782_R_pmrC/1-548   NCLQKQAQTQ HYSQDNLFST LLGLTGVETK YYQAADDILQ TCRRVSE*
707671-17_00426_R_pmrC/1-548    NCLQKQAQTQ HYSQDNLFST LLGLTGVETK YYQAADDILQ TCRRVSE*
700099-17_03186_R_pmrC/1-548    NCLQKQAQTQ HYSQDNLFST LLGLTGVETK YYQAADDILQ TCRRVSE*
700455-17_00207_R_pmrC/1-548    NCLQKQAQTQ HYSQDNLFST LLGLTGVETK YYQAADDILQ TCRRVSE*
709006-16__R_pmrC/1-548         NCLQKQAQTQ HYSQDNLFST LLGLTGVETK YYQAADDILQ TCRRVSE*
705498-12_03775_R_pmrC/1-548    NCLQKQAQTQ HYSQDNLFST LLGLTGVETK YYQAADDILQ TCRRVSE*
706090-16_01483_R_pmrC/1-548    NCLQKQAQTQ HYSQDNLFST LLGLTGVETK YYQAADDILQ TCRRVSE*
719645-16_03124_R_pmrC/1-548    NCLQKQAQTQ HYSQDNLFST LLGLTGVETK YYQAADDILQ TCRRVSE*
721296-16_02906_R_pmrC/1-548    NCLQKQAQTQ HYSQDNLFST LLGLTGVETK YYQAADDILQ TCRRVSE*
```

Alignment: D:\Documentos BASILEA\AMR LAB\Projects\Colistin resistance project\Genes\E. coli gen
Seaview [blocks=10 fontsize=10 A4] on Fri Aug 14 09:57:55 2020

```
                                1
108627-17_04206_S_phoQ/1-487    MKKLLHLFFP LSLRVRFLLA TAAVVLVLSL AYGMVALIGY SVSFDKTTFR
14929959_04296_S_phoQ/1-487     MKKLLRLFFP LSLRVRFLLA TAAVVLVLSL AYGMVALIGY SVSFDKTTFR
ATCC-25922_03515_S_phoQ/1-487   MKKLLHLFFP LSLRVRFLLA TAAVVLVLSL AYGMVALIGY SVSFDKTTFR
108670-17_01835_S_phoQ/1-487    MKKLLHLFFP LSLRVRFLLA TAAVVLVLSL AYGMVALIGY SVSFDKTTFR
603351-17_02818_S_phoQ/1-487    MKKLLHLFFP LSLRVRFLLA TAAVVLVLSL AYGMVALIGY SVSFDKTTFR
703629-17_02557_S_phoQ/1-487    MKKLLRLFFP LSLRVRFLLA TAAVVLVLSL AYGMVALIGY SVSFDKTTFR
703692-17_03870_S_phoQ/1-487    MKKLLHLFFP LSLRVRFLLA TAAVVLVLSL AYGMVALIGY SVSFDKTTFR
703694-17_02947_S_phoQ/1-487    MKKLLRLFFP LSLRVRFLLA TAAVVLVLSL AYGMVALIGY SVSFDKTTFR
705882-17_03640_S_phoQ/1-487    MKKLLRLFFP LSLRVRFLLA TAAVVLVLSL AYGMVALIGY SVSFDKTTFR
705889-17_01549_S_phoQ/1-487    MKKLLRLFFP LSLRVRFLLA TAAVVLVLSL AYGMVALIGY SVSFDKTTFR
722306-16_01057_S_phoQ/1-454    MKKLLRLFFP LSLRVRFLLA TAAVVLVLSL AYGMVALIGY SVSFDKTTFR
705801-17_04900_S_phoQ/1-487    MKKLLRLFFP LSLRVRFLLA TAAVVLVLSL AYGMVALIGY SVSFDKTTFR
NCTC-13846_00898_R_phoQ/1-487   MKKLLRLFFP LSLRVRFLLA TAAVVLVLSL AYGMVALIGY SVSFDKTTFR
700099-17_01720_R_phoQ/1-487    MKKLLRLFFP LSLRVRFLLA TAAVVLVLSL AYGMVALIGY SVSFDKTTFR
700455-17_04539_R_phoQ/1-487    MKKLLHLFFP LSLRVRFLLA TAAVVLVLSL AYGMVALIGY SVSFDKTTFR
705498-12_01206_R_phoQ/1-487    MKKLLHLFFP LSLRVRFLLA TAAVVLVLSL AYGMVALIGY SVSFDKTTFR
705963-16_03914_R_phoQ/1-487    MKKLLRLFFP LSLRVRFLLA TAAVVLVLSL AYGMVALIGY SVSFDKTTFR
706090-16_02994_R_phoQ/1-487    MKKLLHLFFP LSLRVRFLLA TAAVVLVLSL AYGMVALIGY SVSFDKTTFR
707671-17_00856_R_phoQ/1-487    MKKLLRLFFP LSLRVRFLLA TAAVVLVLSL AYGMVALIGY SVSFDKTTFR
709006-16_01084_R_phoQ/1-487    MKKLLRLFFP LSLRVRFLLA TAAVVLVLSL AYGMVALIGY SVSFDKTTFR
719645-16_02744_R_phoQ/1-487    MKKLLRLFFP LSLRVRFLLA TAAVVLVLSL AYGMVALIGY SVSFDKTTFR
721296-16_01818_R_phoQ/1-487    MKKLLRLFFP LSLRVRFLLA TAAVVLVLSL AYGMVALIGY SVSFDKTTFR
KP-37_04232_R_phoQ/1-487        MKKLLRLFFP LSLRVRFLLA TAAVVLVLSL AYGMVALIGY SVSFDKTTFR

                               51
108627-17_04206_S_phoQ/1-487    LLRGESNLFY TLAKWENNKL HVELPENIDK QSPTMTLIYD ENGQLLWAQR
14929959_04296_S_phoQ/1-487     LLRGESNLFY TLAKWENNKL HVELPENIDK QSPTMTLIYD ENGQLLWAQR
ATCC-25922_03515_S_phoQ/1-487   LLRGESNLFY TLAKWENNKL HVELPENIDK QSPTMTLIYD ENGQLLWAQR
108670-17_01835_S_phoQ/1-487    LLRGESNLFY TLAKWENNKL HVELPENIDK QSPTMTLIYD ENGQLLWAQR
603351-17_02818_S_phoQ/1-487    LLRGESNLFY TLAKWENNKL HVELPENIDK QSPTMTLIYD ENGQLLWAQR
703629-17_02557_S_phoQ/1-487    LLRGESNLFY TLAKWENNKL HVELPENIDK QSPTMTLIYD ENGQLLWAQR
703692-17_03870_S_phoQ/1-487    LLRGESNLFY TLAKWENNKL HVELPENIDK QSPTMTLIYD ENGQLLWAQR
703694-17_02947_S_phoQ/1-487    LLRGESNLFY TLAKWENNKL HVELPENIDK QSPTMTLIYD ENGQLLWAQR
705882-17_03640_S_phoQ/1-487    LLRGESNLFY TLAKWENNKL HVELPENIDK QSPTMTLIYD ENGQLLWAQR
705889-17_01549_S_phoQ/1-487    LLRGESNLFY TLAKWENNKL HVELPENIDK QSPTMTLIYD ENGQLLWAQR
722306-16_01057_S_phoQ/1-454    LLRGESNLFY TLAKWENNKL HVELPENIDK QSPTMTLIYD ENGQLLWAQR
705801-17_04900_S_phoQ/1-487    LLRGESNLFY TLAKWENNKL HVELPENIDK QSPTMTLIYD ENGQLLWAQR
NCTC-13846_00898_R_phoQ/1-487   LLRGESNLFY TLAKWENNKL HVELPENIDK QSPTMTLIYD ENGQLLWAQR
700099-17_01720_R_phoQ/1-487    LLRGESNLFY TLAKWENNKL HVELPENIDK QSPTMTLIYD ENGQLLWAQR
700455-17_04539_R_phoQ/1-487    LLRGESNLFY TLAKWENNKL HVELPENIDK QSPTMTLIYD ENGQLLWAQR
705498-12_01206_R_phoQ/1-487    LLRGESNLFY TLAKWENNKL HVELPENIDK QSPTMTLIYD ENGQLLWAQR
705963-16_03914_R_phoQ/1-487    LLRGESNLFY TLAKWENNKL HVELPENIDK QSPTMTLIYD ENGQLLWAQR
706090-16_02994_R_phoQ/1-487    LLRGESNLFY TLAKWENNKL HVELPENIDK QSPTMTLIYD ENGQLLWAQR
707671-17_00856_R_phoQ/1-487    LLRGESNLFY TLAKWENNKL HVELPENIDK QSPTMTLIYD ENGQLLWAQR
709006-16_01084_R_phoQ/1-487    LLRGESNLFY TLAKWENNKL HVELPENIDK QSPTMTLIYD ENGQLLWAQR
719645-16_02744_R_phoQ/1-487    LLRGESNLFY TLAKWENNKL HVELPENIDK QSPTMTLIYD ENGQLLWAQR
721296-16_01818_R_phoQ/1-487    LLRGESNLFY TLAKWENNKL HVELPENIDK QSPTMTLIYD ENGQLLWAQR
KP-37_04232_R_phoQ/1-487        LLRGESNLFY TLAKWENNKL HVELPENIDK QSPTMTLIYD ENGQLLWAQR

                              101
108627-17_04206_S_phoQ/1-487    DVPWLMKMIQ PDWLKSNGFH EIEADVNDTS LLLSGDHSIQ QQLQEVREDD
14929959_04296_S_phoQ/1-487     DVPWLMKMIQ PDWLKSNGFH EIEADVNDTS LLLSGDHSIQ QQLQEVREDD
ATCC-25922_03515_S_phoQ/1-487   DVPWLMKMIQ PDWLKSNGFH EIEADVNDTS LLLSGDHSIQ QQLQEVREDD
108670-17_01835_S_phoQ/1-487    DVPWLMKMIQ PDWLKSNGFH EIEADVNDTS LLLSGDHSIQ QQLQEVREDD
603351-17_02818_S_phoQ/1-487    DVPWLMKMIQ PDWLKSNGFH EIEADVNDTS LLLSGDHSIQ QQLQEVREDD
703629-17_02557_S_phoQ/1-487    DVPWLMKMIQ PDWLKSNGFH EIEADVNDTS LLLSGDHSIQ QQLQEVREDD
703692-17_03870_S_phoQ/1-487    DVPWLMKMIQ PDWLKSNGFH EIEADVNDTS LLLSGDHSIQ QQLQEVREDD
703694-17_02947_S_phoQ/1-487    DVPWLMKMIQ PDWLKSNGFH EIEADVNDTS LLLSGDHSIQ QQLQEVREDD
705882-17_03640_S_phoQ/1-487    DVPWLMKMIQ PDWLKSNGFH EIEADVNDTS LLLSGDHTIQ QQLQEVREDD
705889-17_01549_S_phoQ/1-487    DVPWLMKMIQ PDWLKSNGFH EIEADVNDTS LLLSGDHSIQ QQLQEVREDD
722306-16_01057_S_phoQ/1-454    DVPWLMKMIQ PDWLKSNGFH EIEADVNDTS LLLSGDHSIQ QQLQEVREDD
705801-17_04900_S_phoQ/1-487    DVPWLMKMIQ PDWLKSNGFH EIEADVNDTS LLLSGDHSIQ QQLQEVREDD
NCTC-13846_00898_R_phoQ/1-487   DVPWLMKMIQ PDWLKSNGFH EIEADVNDTS LLLSGDHSIQ QQLQEVREDD
700099-17_01720_R_phoQ/1-487    DVPWLMKMIQ PDWLKSNGFH EIEADVNDTS LLLSGDHSIQ QQLQEVREDD
700455-17_04539_R_phoQ/1-487    DVPWLMKMIQ PDWLKSNGFH EIEADVNDTS LLLSGDHSIQ QQLQEVREDD
705498-12_01206_R_phoQ/1-487    DVPWLMKMIQ PDWLKSNGFH EIEADVNDTS LLLSGDHSIQ QQLQEVREDD
705963-16_03914_R_phoQ/1-487    DVPWLMKMIQ PDWLKSNGFH EIEADVNDTS LLLSGDHSIQ QQLQEVREDD
706090-16_02994_R_phoQ/1-487    DVPWLMKMIQ PDWLKSNGFH EIEADVNDTS LLLSGDHSIQ QQLQEVREDD
707671-17_00856_R_phoQ/1-487    DVPWLMKMIQ PDWLKSNGFH EIEADVNDTS LLLSGDHSIQ QQLQEVREDD
709006-16_01084_R_phoQ/1-487    DVPWLMKMIQ PDWLKSNGFH EIEADVNDTS LLLSGDHSIQ QQLQEVREDD
719645-16_02744_R_phoQ/1-487    DVPWLMKMIQ PDWLKSNGFH EIEADVNDTS LLLSGDHSIQ QQLQEVREDD
721296-16_01818_R_phoQ/1-487    DVPWLMKMIQ PDWLKSNGFH EIEADVNDTS LLLSGDHSIQ QQLQEVREDD
KP-37_04232_R_phoQ/1-487        DVPWLMKMIQ PDWLKSNGFH EIEADVNDTS LLLSGDHSIQ QQLQEVREDD
```

```
                                 151
108627-17_04206_S_phoQ/1-487      DDAEMTHSVA VNVYPATSRM PKLTIVVVDT IPVELKSSYM VWSWFIYVLS
14929959_04296_S_phoQ/1-487       DDAEMTHSVA VNVYPATSRM PKLTIVVVDT IPVELKSSYM VWSWFIYVLS
ATCC-25922_03515_S_phoQ/1-487     DDAEMTHSVA VNVYPATSRM PKLTIVVVDT IPVELKSSYM VWSWFIYVLS
108670-17_01835_S_phoQ/1-487      DDAEMTHSVA VNVYPATSRM PKLTIVVVDT IPVELKSSYM VWSWFIYVLS
603351-17_02818_S_phoQ/1-487      DDAEMTHSVA VNVYPATSRM PKLTIVVVDT IPVELKSSYM VWSWFIYVLS
703629-17_02557_S_phoQ/1-487      DDAEMTHSVA VNVYPATSRM PKLTIVVVDT IPVELKSSYM VWSWFIYVLS
703692-17_03870_S_phoQ/1-487      DDAEMTHSVA VNVYPATSRM PKLTIVVVDT IPVELKSSYM VWSWFIYVLS
703694-17_02947_S_phoQ/1-487      DDAEMTHSVA VNVYPATSRM PKLTIVVVDT IPVELKSSYM VWSWFIYVLS
705882-17_03640_S_phoQ/1-487      DDAEMTHSVA VNVYPATSRM PKLTIVVVDT IPVELKSSYM VWSWFIYVLS
705889-17_01549_S_phoQ/1-487      DDAEMTHSVA VNVYPATSRM PKLTIVVVDT IPVELKSSYM VWSWFIYVLS
722306-16_01057_S_phoQ/1-454      DDAEMTHSVA VNVYPATSRM PKLTIVVVDT IPVELKSSYM VWSWFIYVLS
705801-17_04900_S_phoQ/1-487      DDAEMTHSVA VNVYPATSRM PKLTIVVVDT IPVELKSSYM VWSWFIYVLS
NCTC-13846_00898_R_phoQ/1-487     DDAEMTHSVA VNVYPATSRM PKLTIVVVDT IPVELKSSYM VWSWFIYVLS
700099-17_01720_R_phoQ/1-487      DDAEMTHSVA VNVYPATSRM PKLTIVVVDT IPVELKSSYM VWSWFIYVLS
700455-17_04539_R_phoQ/1-487      DDAEMTHSVA VNVYPATSRM PKLTIVVVDT IPVELKSSYM VWSWFIYVLS
705498-12_01206_R_phoQ/1-487      DDAEMTHSVA VNVYPATSRM PKLTIVVVDT IPVELKSSYM VWSWFIYVLS
705963-16_03914_R_phoQ/1-487      DDAEMTHSVA VNVYPATSRM PKLTIVVVDT IPVELKSSYM VWSWFIYVLS
706090-16_02994_R_phoQ/1-487      DDAEMTHSVA VNVYPATSRM PKLTIVVVDT IPVELKSSYM VWSWFIYVLS
707671-17_00856_R_phoQ/1-487      DDAEMTHSVA VNVYPATSRM PKLTIVVVDT IPVELKSSYM VWSWFIYVLS
709006-16_01084_R_phoQ/1-487      DDAEMTHSVA VNVYPATSRM PKLTIVVVDT IPVELKSSYM VWSWFIYVLS
719645-16_02744_R_phoQ/1-487      DDAEMTHSVA VNVYPATSRM PKLTIVVVDT IPVELKSSYM VWSWFIYVLS
721296-16_01818_R_phoQ/1-487      DDAEMTHSVA VNVYPATSRM PKLTIVVVDT IPVELKSSYM VWSWFIYVLS
KP-37_04232_R_phoQ/1-487          DDAEMTHSVA VNVYPATSRM PKLTIVVVDT IPVELKSSYM VWSWFIYVLS

                                 201
108627-17_04206_S_phoQ/1-487      ANLLLVIPLL WVAAWWSLRP IEALAKEVRE LEEHNRELLN PATTRELTSL
14929959_04296_S_phoQ/1-487       ANLLLVIPLL WVAAWWSLRP IEALAKEVRE LEEHNRELLN PATTRELTSL
ATCC-25922_03515_S_phoQ/1-487     ANLLLVIPLL WVAAWWSLRP IEALAKEVRE LEEHNRELLN PATTRELTSL
108670-17_01835_S_phoQ/1-487      ANLLLVIPLL WVAAWWSLRP IEALAKEVRE LEEHNRELLN PATTRELTSL
603351-17_02818_S_phoQ/1-487      ANLLLVIPLL WVAAWWSLRP IEALAKEVRE LEEHNRELLN PATTRELTSL
703629-17_02557_S_phoQ/1-487      ANLLLVIPLL WVAAWWSLRP IEALAKEVRE LEEHNRELLN PATTRELTSL
703692-17_03870_S_phoQ/1-487      ANLLLVIPLL WVAAWWSLRP IEALAKEVRE LEEHNRELLN PATTRELTSL
703694-17_02947_S_phoQ/1-487      ANLLLVIPLL WVAAWWSLRP IEALAKEVRE LEEHNRELLN PATTRELTSL
705882-17_03640_S_phoQ/1-487      ANLLLVIPLL WVAAWWSLRP IEALAKEVRE LEEHNRELLN PATTRELTSL
705889-17_01549_S_phoQ/1-487      ANLLLVIPLL WVAAWWSLRP IEALAKEVRE LEEHNRELLN PATTRELTSL
722306-16_01057_S_phoQ/1-454      ANLLLVIPLL WVAAWWSLRP IEALAKEVRE LEEHNRELLN PATTRELTSL
705801-17_04900_S_phoQ/1-487      ANLLLVIPLL WVAAWWSLRP IEALAKEVRE LEEHNRELLN PATTRELTSL
NCTC-13846_00898_R_phoQ/1-487     ANLLLVIPLL WVAAWWSLRP IEALAKEVRE LEEHNRELLN PATTRELTSL
700099-17_01720_R_phoQ/1-487      ANLLLVIPLL WVAAWWSLRP IEALAKEVRE LEEHNRELLN PATTRELTSL
700455-17_04539_R_phoQ/1-487      ANLLLVIPLL WVAAWWSLRP IEALAKEVRE LEEHNRELLN PATTRELTSL
705498-12_01206_R_phoQ/1-487      ANLLLVIPLL WVAAWWSLRP IEALAKEVRE LEEHNRELLN PATTRELTSL
705963-16_03914_R_phoQ/1-487      ANLLLVIPLL WVAAWWSLRP IEALAKEVRE LEEHNRELLN PATTRELTSL
706090-16_02994_R_phoQ/1-487      ANLLLVIPLL WVAAWWSLRP IEALAKEVRE LEEHNRELLN PATTRELTSL
707671-17_00856_R_phoQ/1-487      ANLLLVIPLL WVAAWWSLRP IEALAKEVRE LEEHNRELLN PATTRELTSL
709006-16_01084_R_phoQ/1-487      ANLLLVIPLL WVAAWWSLRP IEALAKEVRE LEEHNRELLN PATTRELTSL
719645-16_02744_R_phoQ/1-487      ANLLLVIPLL WVAAWWSLRP IEALAKEVRE LEEHNRELLN PATTRELTSL
721296-16_01818_R_phoQ/1-487      ANLLLVIPLL WVAAWWSLRP IEALAKEVRE LEEHNRELLN PATTRELTSL
KP-37_04232_R_phoQ/1-487          ANLLLVIPLL WVAAWWSLRP IEALAKEVRE LEEHNRELLN PATTRELTSL

                                 251
108627-17_04206_S_phoQ/1-487      VRNLNRLLKS ERERYDKYRT TLTDLTHSLK TPLAVLQSTL RSLRSEKMSV
14929959_04296_S_phoQ/1-487       VRNLNRLLKS ERERYDKYRT TLTDLTHSLK TPLAVLQSTL RSLRSEKMSV
ATCC-25922_03515_S_phoQ/1-487     VRNLNRLLKS ERERYDKYRT TLTDLTHSLK TPLAVLQSTL RSLRSEKMSV
108670-17_01835_S_phoQ/1-487      VRNLNRLLKS ERERYDKYRT TLTDLTHSLK TPLAVLQSTL RSLRSEKMSV
603351-17_02818_S_phoQ/1-487      VRNLNRLLKS ERERYDKYRT TLTDLTHSLK TPLAVLQSTL RSLRSEKMSV
703629-17_02557_S_phoQ/1-487      VRNLNRLLKS ERERYDKYRT TLTDLTHSLK TPLAVLQSTL RSLRSEKMSV
703692-17_03870_S_phoQ/1-487      VRNLNRLLKS ERERYDKYRT TLTDLTHSLK TPLAVLQSTL RSLRSEKMSV
703694-17_02947_S_phoQ/1-487      VRNLNRLLKS ERERYDKYRT TLTDLTHSLK TPLAVLQSTL RSLRSEKMSV
705882-17_03640_S_phoQ/1-487      VRNLNRLLKS ERERYDKYRT TLTDLTHSLK TPLAVLQSTL RSLRSEKMSV
705889-17_01549_S_phoQ/1-487      VRNLNRLLKS ERERYDKYRT TLTDLTHSLK TPLAVLQSTL RSLRSEKMSV
722306-16_01057_S_phoQ/1-454      VRNLNRLLKS ERERYDKYRT TLTDLTHSLK TPLAVLQSTL RSLRSEKMSV
705801-17_04900_S_phoQ/1-487      VRNLNRLLKS ERERYDKYRT TLTDLTHSLK TPLAVLQSTL RSLRSEKMSV
NCTC-13846_00898_R_phoQ/1-487     VRNLNRLLKS ERERYDKYRT TLTDLTHSLK TPLAVLQSTL RSLRSEKMSV
700099-17_01720_R_phoQ/1-487      VRNLNRLLKS ERERYDKYRT TLTDLTHSLK TPLAVLQSTL RSLRSEKMSV
700455-17_04539_R_phoQ/1-487      VRNLNRLLKS ERERYDKYRT TLTDLTHSLK TPLAVLQSTL RSLRSEKMSV
705498-12_01206_R_phoQ/1-487      VRNLNRLLKS ERERYDKYRT TLTDLTHSLK TPLAVLQSTL RSLRSEKMSV
705963-16_03914_R_phoQ/1-487      VRNLNRLLKS ERERYDKYRT TLTDLTHSLK TPLAVLQSTL RSLRSEKMSV
706090-16_02994_R_phoQ/1-487      VRNLNRLLKS ERERYDKYRT TLTDLTHSLK TPLAVLQSTL RSLRSEKMSV
707671-17_00856_R_phoQ/1-487      VRNLNRLLKS ERERYDKYRT TLTDLTHSLK TPLAVLQSTL RSLRSEKMSV
709006-16_01084_R_phoQ/1-487      VRNLNRLLKS ERERYDKYRT TLTDLTHSLK TPLAVLQSTL RSLRSEKMSV
719645-16_02744_R_phoQ/1-487      VRNLNRLLKS ERERYDKYRT TLTDLTHSLK TPLAVLQSTL RSLRSEKMSV
721296-16_01818_R_phoQ/1-487      VRNLNRLLKS ERERYDKYRT TLTDLTHSLK TPLAVLQSTL RSLRSEKMSV
KP-37_04232_R_phoQ/1-487          VRNLNRLLKS ERERYDKYRT TLTDLTHSLK TPLAVLQSTL RSLRSEKMSV
```

```
                                  301
108627-17_04206_S_phoQ/1-487      SDAEPVMLEQ ISRISQQIGY YLHRASMRGG TLLSRELHPV APLLDNLTSA
14929959_04296_S_phoQ/1-487       SDAEPVMLEQ ISRISQQIGY YLHRASMRGG TLLSRELHPV APLLDNLTSA
ATCC-25922_03515_S_phoQ/1-487     SDAEPVMLEQ ISRISQQIGY YLHRASMRGG TLLSRELHPV APLLDNLTSA
108670-17_01835_S_phoQ/1-487      SDAEPVMLEQ ISRISQQIGY YLHRASMRGG TLLSRELHPV APLLDNLTSA
603351-17_02818_S_phoQ/1-487      SDAEPVMLEQ ISRISQQIGY YLHRASMRGG TLLSRELHPV APLLDNLTSA
703629-17_02557_S_phoQ/1-487      SDAEPVMLEQ ISRISQQIGY YLHRASMRGG TLLSRELHPV APLLDNLTSA
703692-17_03870_S_phoQ/1-487      SDAEPVMLEQ ISRISQQIGY YLHRASMRGG TLLSRELHPV APLLDNLTSA
703694-17_02947_S_phoQ/1-487      SDAEPVMLEQ ISRISQQIGY YLHRASMRGG TLLSRELHPV APLLDNLTSA
705882-17_03640_S_phoQ/1-487      SDAEPVMLEQ ISRISQQIGY YLHRASMRGG TLLSRELHPV APLLDNLTSA
705889-17_01549_S_phoQ/1-487      SDAEPVMLEQ ISRISQQIGY YLHRASMRGG TLLSRELHPV APLLDNLTSA
722306-16_01057_S_phoQ/1-454      SDAEPVMLEQ ISRISQQIGY YLHRASMRGG TLLSRELHPV APLLDNLTSA
705801-17_04900_S_phoQ/1-487      SDAEPVMLEQ ISRISQQIGY YLHRASMRGG TLLSRELHPV APLLDNLTSA
NCTC-13846_00898_R_phoQ/1-487     SDAEPVMLEQ ISRISQQIGY YLHRASMRGG TLLSRELHPV APLLDNLTSA
700099-17_01720_R_phoQ/1-487      SDAEPVMLEQ ISRISQQIGY YLHRASMRGG TLLSRELHPV APLLDNLTSA
700455-17_04539_R_phoQ/1-487      SDAEPVMLEQ ISRISQQIGY YLHRASMRGG TLLSRELHPV APLLDNLTSA
705498-12_01206_R_phoQ/1-487      SDAEPVMLEQ ISRISQQIGY YLHRASMRGG TLLSRELHPV APLLDNLTSA
705963-16_03914_R_phoQ/1-487      SDAEPVMLEQ ISRISQQIGY YLHRASMRGG TLLSRELHPV APLLDNLTSA
706090-16_02994_R_phoQ/1-487      SDAEPVMLEQ ISRISQQIGY YLHRASMRGG TLLSRELHPV APLLDNLTSA
707671-17_00856_R_phoQ/1-487      SDAEPVMLEQ ISRISQQIGY YLHRASMRGG TLLSRELHPV APLLDNLTSA
709006-16_01084_R_phoQ/1-487      SDAEPVMLEQ ISRISQQIGY YLHRASMRGG TLLSRELHPV APLLDNLTSA
719645-16_02744_R_phoQ/1-487      SDAEPVMLEQ ISRISQQIGY YLHRASMRGG TLLSRELHPV APLLDNLTSA
721296-16_01818_R_phoQ/1-487      SDAEPVMLEQ ISRISQQIGY YLHRASMRGG TLLSRELHPV APLLDNLTSA
KP-37_04232_R_phoQ/1-487          SDAEPVMLEQ ISRISQQIGY YLHRASMRGG TLLSRELHPV APLLDNLTSA

                                  351
108627-17_04206_S_phoQ/1-487      LNKVYQRKGV NISLDISPEI SFVGEQNDFV EVMGNVLDNA CKYCLEFVEI
14929959_04296_S_phoQ/1-487       LNKVYQRKGV NISLDISPEI SFVGEQNDFV EVMGNVLDNA CKYCLEFVEI
ATCC-25922_03515_S_phoQ/1-487     LNKVYQRKGV NISLDISPEI SFVGEQNDFV EVMGNVLDNA CKYCLEFVEI
108670-17_01835_S_phoQ/1-487      LNKVYQRKGV NISLDISPEI SFVGEQNDFV EVMGNVLDNA CKYCLEFVEI
603351-17_02818_S_phoQ/1-487      LNKVYQRKGV NISLDISPEI SFVGEQNDFV EVMGNVLDNA CKYCLEFVEI
703629-17_02557_S_phoQ/1-487      LNKVYQRKGV NISLDISPEI SFVGEQNDFV EVMGNVLDNA CKYCLEFVEI
703692-17_03870_S_phoQ/1-487      LNKVYQRKGV NISLDISPEI SFVGEQNDFV EVMGNVLDNA CKYCLEFVEI
703694-17_02947_S_phoQ/1-487      LNKVYQRKGV NISLDISPEI SFVGEQNDFV EVMGNVLDNA CKYCLEFVEI
705882-17_03640_S_phoQ/1-487      LNKVYQRKGV NISLDISPEI SFVGEQNDFV EVMGNVLDNA CKYCLEFVEI
705889-17_01549_S_phoQ/1-487      LNKVYQRKGV NISLDISPEI SFVGEQNDFV EVMGNVLDNA CKYCLEFVEI
722306-16_01057_S_phoQ/1-454      LNKVYQRKGV NISLDISPEI SFVGEQNDFV EVMGNVLDNA CKYCLEFVEI
705801-17_04900_S_phoQ/1-487      LNKVYQRKGV NISLDISPEI SFVGEQNDFV EVMGNVLDNA CKYCLEFVEI
NCTC-13846_00898_R_phoQ/1-487     LNKVYQRKGV NISLDISPEI SFVGEQNDFV EVMGNVLDNA CKYCLEFVEI
700099-17_01720_R_phoQ/1-487      LNKVYQRKGV NISLDISPEI SFVGEQNDFV EVMGNVLDNA CKYCLEFVEI
700455-17_04539_R_phoQ/1-487      LNKVYQRKGV NISLDISPEI SFVGEQNDFV EVMGNVLDNA CKYCLEFVEI
705498-12_01206_R_phoQ/1-487      LNKVYQRKGV NISLDISPEI SFVGEQNDFV EVMGNVLDNA CKYCLEFVEI
705963-16_03914_R_phoQ/1-487      LNKVYQRKGV NISLDISPEI SFVGEQNDFV EVMGNVLDNA CKYCLEFVEI
706090-16_02994_R_phoQ/1-487      LNKVYQRKGV NISLDISPEI SFVGEQNDFV EVMGNVLDNA CKYCLEFVEI
707671-17_00856_R_phoQ/1-487      LNKVYQRKGV NISLDISPEI SFVGEQNDFV EVMGNVLDNA CKYCLEFVEI
709006-16_01084_R_phoQ/1-487      LNKVYQRKGV NISLDISPEI SFVGEQNDFV EVMGNVLDNA CKYCLEFVEI
719645-16_02744_R_phoQ/1-487      LNKVYQRKGV NISLDISPEI SFVGEQNDFV EVMGNVLDNA CKYCLEFVEI
721296-16_01818_R_phoQ/1-487      LNKVYQRKGV NISLDISPEI SFVGEQNDFV EVMGNVLDNA CKYCLEFVEI
KP-37_04232_R_phoQ/1-487          LNKVYQRKGV NISLDISPEI SFVGEQNDFV EVMGNVLDNA CKYCLEFVEI

                                  401
108627-17_04206_S_phoQ/1-487      SARQTDEHLY IVVEDDGPGI PLSKREVIFD RGQRVDTLRP GQGVGLAVAR
14929959_04296_S_phoQ/1-487       SARQTDEHLY IVVEDDGPGI PLSKREVIFD RGQRVDTLRP GQGVGLAVAR
ATCC-25922_03515_S_phoQ/1-487     SARQTDEHLY IVVEDDGPGI PLSKREVIFD RGQRVDTLRP GQGVGLAVAR
108670-17_01835_S_phoQ/1-487      SARQTDEHLY IVVEDDGPGI PLSKREVIFD RGQRVDTLRP GQGVGLAVAR
603351-17_02818_S_phoQ/1-487      SARQTDEHLY IVVEDDGPGI PLSKREVIFD RGQRVDTLRP GQGVGLAVAR
703629-17_02557_S_phoQ/1-487      SARQTDEHLY IVVEDDGPGI PLSKREVIFD RGQRVDTLRP GQGVGLAVAR
703692-17_03870_S_phoQ/1-487      SARQTDEHLY IVVEDDGPGI PLSKREVIFD RGQRVDTLRP GQGVGLAVAR
703694-17_02947_S_phoQ/1-487      SARQTDEHLY IVVEDDGPGI PLSKREVIFD RGQRVDTLRP GQGVGLAVAR
705882-17_03640_S_phoQ/1-487      SARQTDEHLY IVVEDDGPGI PLSKREVIFD RGQRVDTLRP GQGVGLAVAR
705889-17_01549_S_phoQ/1-487      SARQTDEHLY IVVEDDGPGI PLSKREVIFD RGQRVDTLRP GQGVGLAVAR
722306-16_01057_S_phoQ/1-454      SARQTDEHLY IVVEDDGPGI PLSKREVIFD RGQRVDTLRP GQGVGLAVAR
705801-17_04900_S_phoQ/1-487      SARQTDEHLY IVVEDDGPGI PLSKREVIFD RGQRVDTLRP GQGVGLAVAR
NCTC-13846_00898_R_phoQ/1-487     SARQTDEHLY IVVEDDGPGI PLSKREVIFD RGQRVDTLRP GQGVGLAVAR
700099-17_01720_R_phoQ/1-487      SARQTDEHLY IVVEDDGPGI PLSKREVIFD RGQRVDTLRP GQGVGLAVAR
700455-17_04539_R_phoQ/1-487      SARQTDEHLY IVVEDDGPGI PLSKREVIFD RGQRVDTLRP GQGVGLAVAR
705498-12_01206_R_phoQ/1-487      SARQTDEHLY IVVEDDGPGI PLSKREVIFD RGQRVDTLRP GQGVGLAVAR
705963-16_03914_R_phoQ/1-487      SARQTDEHLY IVVEDDGPGI PLSKREVIFD RGQRVDTLRP GQGVGLAVAR
706090-16_02994_R_phoQ/1-487      SARQTDEHLY IVVEDDGPGI PLSKREVIFD RGQRVDTLRP GQGVGLAVAR
707671-17_00856_R_phoQ/1-487      SARQTDEHLY IVVEDDGPGI PLSKREVIFD RGQRVDTLRP GQGVGLAVAR
709006-16_01084_R_phoQ/1-487      SARQTDEHLY IVVEDDGPGI PLSKREVIFD RGQRVDTLRP GQGVGLAVAR
719645-16_02744_R_phoQ/1-487      SARQTDEHLY IVVEDDGPGI PLSKREVIFD RGQRVDTLRP GQGVGLAVAR
721296-16_01818_R_phoQ/1-487      SARQTDEHLY IVVEDDGPGI PLSKREVIFD RGQRVDTLRP GQGVGLAVAR
KP-37_04232_R_phoQ/1-487          SARQTDEHLY IVVEDDGPGI PLSKREVIFD RGQRVDTLRP GQGVGLAVAR
```

```
                                451
108627-17_04206_S_phoQ/1-487    EITEQYEGKI VAGESMLGGA RMEVIFGRQH SAPKDE*
14929959_04296_S_phoQ/1-487     EITEQYEGKI VAGESMLGGA RMEVIFGRQH SAPKDE*
ATCC-25922_03515_S_phoQ/1-487   EITEQYEGKI VAGESMLGGA RMEVIFGRQH SAPKDE*
108670-17_01835_S_phoQ/1-487    EITEQYEGKI VAGESMLGGA RMEVIFGRQH SAPKDE*
603351-17_02818_S_phoQ/1-487    EITEQYEGKI VAGESMLGGA RMEVIFGRQH SAPKDE*
703629-17_02557_S_phoQ/1-487    EITEQYEGKI VAGESMLGGA RMEVIFGRQH SAPKDE*
703692-17_03870_S_phoQ/1-487    EITEQYEGKI VAGESMLGGA RMEVIFGRQH SAPKDE*
703694-17_02947_S_phoQ/1-487    EITEQYEGKI VAGESMLGGA RMEVIFGRQH SAPKDE*
705882-17_03640_S_phoQ/1-487    EITEQYEGKI VAGESMLGGA RMEVIFGRQH STPKDE*
705889-17_01549_S_phoQ/1-487    EITEQYEGKI VAGESMLGGA RMEVIFGRQH SAPKDE*
722306-16_01057_S_phoQ/1-454    EIT*------ ---------- ---------- -------
705801-17_04900_S_phoQ/1-487    EITEQYEGKI VAGESMLGGA RMEVIFGRQH SAPKDE*
NCTC-13846_00898_R_phoQ/1-487   EITEQYEGKI VAGESMMGGA RMEVIFGRQH SAPKDE*
700099-17_01720_R_phoQ/1-487    EITEQYEGKI VAGESMLGGA RMEVIFGRQH SAPKDE*
700455-17_04539_R_phoQ/1-487    EITEQYEGKI VAGESMLGGA RMEVIFGRQH SAPKDE*
705498-12_01206_R_phoQ/1-487    EITEQYEGKI VAGESMLGGA RMEVIFGRQH SAPKDE*
705963-16_03914_R_phoQ/1-487    EITEQYEGKI VAGESMLGGA RMEVIFGRQH SAPKDE*
706090-16_02994_R_phoQ/1-487    EITEQYEGKI VAGESMLGGA RMEVIFGRQH SAPKDE*
707671-17_00856_R_phoQ/1-487    EITEQYEGKI VAGESMLGGA RMEVIFGRQH SAPKDE*
709006-16_01084_R_phoQ/1-487    EITEQYEGKI VAGESMLGGA RMEVIFGRQH SAPKDE*
719645-16_02744_R_phoQ/1-487    EITEQYEGKI VAGESMLGGA RMEVIFGRQH SAPKDE*
721296-16_01818_R_phoQ/1-487    EITEQYEGKI VAGESMLGGA RMEVIFGRQH SAPKDE*
KP-37_04232_R_phoQ/1-487        EITEQYEGKI VAGESMLGGA RMEVIFGRQH SAPKDE*
```

```
Alignment: D:\Documentos BASILEA\AMR LAB\Projects\Colistin resistance project\Genes\E. coli gen
Seaview [blocks=10 fontsize=10 A4] on Fri Aug 14 09:52:23 2020

                                 1
703692-17_03869_S_phoP/1-224     MRVLVVEDNA LLRHHLKVQI QDAGHQVDDA EDAKEADYYL NEHLPDIAIV
703694-17_02948_S_phoP/1-224     MRVLVVEDNA LLRHHLKVQI QDAGHQVDDA EDAKEADYYL NEHLPDIAIV
705882-17_03639_S_phoP/1-224     MRVLVVEDNA LLRHHLKVQI QDAGHQVDDA EDAKEADYYL NEHLPDIAIV
705889-17_01550_S_phoP/1-224     MRVLVVEDNA LLRHHLKVQI QDAGHQVDDA EDAKEADYYL NEHIPDIAIV
722306-16_01056_S_phoP/1-224     MRVLVVEDNA LLRHHLKVQI QDAGHQVDDA EDAKEADYYL NEHLPDIAIV
14929959_04295_S_phoP/1-224      MRVLVVEDNA LLRHHLKVQI QDAGHQVDDA EDAKEADYYL NEHLPDIAIV
ATCC-25922_03514_S_phoP/1-224    MRVLVVEDNA LLRHHLKVQI QDAGHQVDDA EDAKEADYYL NEHLPDIAIV
108670-17_01836_S_phoP/1-224     MRVLVVEDNA LLRHHLKVQI QDAGHQVDDA EDAKEADYYL NEHLPDIAIV
603351-17_02817_S_phoP/1-224     MRVLVVEDNA LLRHHLKVQI QDAGHQVDDA EDAKEADYYL NEHLPDIAIV
703629-17_02558_S_phoP/1-224     MRVLVVEDNA LLRHHLKVQI QDAGHQVDDA EDAKEADYYL NEHLPDIAIV
705801-17_04901_S_phoP/1-224     MRVLVVEDNA LLRHHLKVQI QDAGHQVDDA EDAKEADYYL NEHLPDIAIV
108627-17_04205_S_phoP/1-224     MRVLVVEDNA LLRHHLKVQI QDAGHQVDDA EDAKEADYYL NEHLPDIAIV
707671-17_00857_R_phoP/1-224     MRVLVVEDNA LLRHHLKVQI QDAGHQVDDA EDAKEADYYL NEHIPDIAIV
709006-16_01085_R_phoP/1-224     MRVLVVEDNA LLRHHLKVQI QDAGHQVDDA EDAKEADYYL NEHLPDIAIV
719645-16_02745_R_phoP/1-224     MRVLVVEDNA LLRHHLKVQI QDAGHQVDDA EDAKEADYYL NEHLPDIAIV
721296-16_01817_R_phoP/1-224     MRVLVVEDNA LLRHHLKVQI QDAGHQVDDA EDAKEADYYL NEHIPDIAIV
KP-37_04233_R_phoP/1-224         MRVLVVEDNA LLRHHLKVQI QDAGHQVDDA EDAKEADYYL NEHIPDIAIV
NCTC-13846_00897_R_phoP/1-224    MRVLVVEDNA LLRHHLKVQI QDAGHQVDDA EDAKEADYYL NEHLPDIAIV
700455-17_04540_R_phoP/1-224     MRVLVVEDNA LLRHHLKVQI QDAGHQVDDA EDAKEADYYL NEHLPDIAIV
705498-12_01205_R_phoP/1-224     MRVLVVEDNA LLRHHLKVQI QDAGHQVDDA EDAKEADYYL NEHLPDIAIV
705963-16_03913_R_phoP/1-224     MRVLVVEDNA LLRHHLKVQI QDAGHQVDDA EDAKEADYYL NEHLPDIAIV
706090-16_02995_R_phoP/1-224     MRVLVVEDNA LLRHHLKVQI QDAGHQVDDA EDAKEADYYL NEHLPDIAIV
700099-17_01721_R_phoP/1-224     MRVLVVEDNA LLRHHLKVQI QDAGHQVDDA EDAKEADYYL NEHLPDIAIV

                                51
703692-17_03869_S_phoP/1-224     DLGLPDEDGL SLIRRWRSND VSLPILVLTA RESWQDKVEV LSAGADDYVT
703694-17_02948_S_phoP/1-224     DLGLPDEDGL SLIRRWRSND VSLPILVLTA RESWQDKVEV LSAGADDYVT
705882-17_03639_S_phoP/1-224     DLGLPDEDGL SLIRRWRSND VSLPILVLTA RESWQDKVEV LSAGADDYVT
705889-17_01550_S_phoP/1-224     DLGLPDEDGL SLIRRWRSND VSLPILVLTA RESWQDKVEV LSAGADDYVT
722306-16_01056_S_phoP/1-224     DLGLPDEDGL SLIRRWRSND VSLPILVLTA RESWQDKVEV LSAGADDYVT
14929959_04295_S_phoP/1-224      DLGLPDEDGL SLIRRWRSND VSLPILVLTA RESWQDKVEV LSAGADDYVT
ATCC-25922_03514_S_phoP/1-224    DLGLPDEDGL SLIRRWRSND VSLPILVLTA RESWQDKVEV LSAGADDYVT
108670-17_01836_S_phoP/1-224     DLGLPDEDGL SLIRRWRSND VSLPILVLTA RESWQDKVEV LSAGADDYVT
603351-17_02817_S_phoP/1-224     DLGLPDEDGL SLIRRWRSND VSLPILVLTA RESWQDKVEV LSAGADDYVT
703629-17_02558_S_phoP/1-224     DLGLPDEDGL SLIRRWRSND VSLPILVLTA RESWQDKVEV LSAGADDYVT
705801-17_04901_S_phoP/1-224     DLGLPDEDGL SLIRRWRSND VSLPILVLTA RESWQDKVEV LSAGADDYVT
108627-17_04205_S_phoP/1-224     DLGLPDEDGL SLIRRWRSND VSLPILVLTA RESWQDKVEV LSAGADDYVT
707671-17_00857_R_phoP/1-224     DLGLPDEDGL SLIRRWRSND VSLPILVLTA RESWQDKVEV LSAGADDYVT
709006-16_01085_R_phoP/1-224     DLGLPDEDGL SLIRRWRSND VSLPILVLTA RESWQDKVEV LSAGADDYVT
719645-16_02745_R_phoP/1-224     DLGLPDEDGL SLIRRWRSND VSLPILVLTA RESWQDKVEV LSAGADDYVT
721296-16_01817_R_phoP/1-224     DLGLPDEDGL SLIRRWRSND VSLPILVLTA RESWQDKVEV LSAGADDYVT
KP-37_04233_R_phoP/1-224         DLGLPDEDGL SLIRRWRSND VSLPILVLTA RESWQDKVEV LSAGADDYVT
NCTC-13846_00897_R_phoP/1-224    DLGLPDEDGL SLIRRWRSND VSLPILVLTA RESWQDKVEV LSAGADDYVT
700455-17_04540_R_phoP/1-224     DLGLPDEDGL SLIRRWRSND VSLPILVLTA RESWQDKVEV LSAGADDYVT
705498-12_01205_R_phoP/1-224     DLGLPDEDGL SLIRRWRSND VSLPILVLTA RESWQDKVEV LSAGADDYVT
705963-16_03913_R_phoP/1-224     DLGLPDEDGL SLIRRWRSND VSLPILVLTA RESWQDKVEV LSAGADDYVT
706090-16_02995_R_phoP/1-224     DLGLPDEDGL SLIRRWRSND VSLPILVLTA RESWQDKVEV LSAGADDYVT
700099-17_01721_R_phoP/1-224     DLGLPDEDGL SLIRRWRSND VSLPILVLTA RESWQDKVEV LSAGADDYVT

                               101
703692-17_03869_S_phoP/1-224     KPFHIEEVMA RMQALMRRNS GLASQVISLP PFQVDLSRRE LSINDEVIKL
703694-17_02948_S_phoP/1-224     KPFHIEEVMA RMQALMRRNS GLASQVISLP PFQVDLSRRE LSINDEVIKL
705882-17_03639_S_phoP/1-224     KPFHIEEVMA RMQALMRRNS GLASQVISLP PFQVDLSRRE LSINDEVIKL
705889-17_01550_S_phoP/1-224     KPFHIEEVMA RMQALMRRNS GLASQVISLP PFQVDLSRRE LSINDEVIKL
722306-16_01056_S_phoP/1-224     KPFHIEEVMA RMQALMRRNS GLASQVISLP PFQVDLSRRE LSINDEVIKL
14929959_04295_S_phoP/1-224      KPFHIEEVMA RMQALMRRNS GLASQVISLP PFQVDLSRRE LSINDEVIKL
ATCC-25922_03514_S_phoP/1-224    KPFHIEEVMA RMQALMRRNS GLASQVISLP PFQVDLSRRE LSINDEVIKL
108670-17_01836_S_phoP/1-224     KPFHIEEVMA RMQALMRRNS GLASQVISLP PFQVDLSRRE LSINDEVIKL
603351-17_02817_S_phoP/1-224     KPFHIEEVMA RMQALMRRNS GLASQVISLP PFQVDLSRRE LSINDEVIKL
703629-17_02558_S_phoP/1-224     KPFHIEEVMA RMQALMRRNS GLASQVISLP PFQVDLSRRE LSINDEVIKL
705801-17_04901_S_phoP/1-224     KPFHIEEVMA RMQALMRRNS GLASQVISLP PFQVDLSRRE LSINDEVIKL
108627-17_04205_S_phoP/1-224     KPFHIEEVMA RMQALMRRNS GLASQVISLP PFQVDLSRRE LSINDEVIKL
707671-17_00857_R_phoP/1-224     KPFHIEEVMA RMQALMRRNS GLASQVISLP PFQVDLSRRE LSINDEVIKL
709006-16_01085_R_phoP/1-224     KPFHIEEVMA RMQALMRRNS GLASQVISLP PFQVDLSRRE LSINDEVIKL
719645-16_02745_R_phoP/1-224     KPFHIEEVMA RMQALMRRNS GLASQVISLP PFQVDLSRRE LSINDEVIKL
721296-16_01817_R_phoP/1-224     KPFHIEEVMA RMQALMRRNS GLASQVISLP PFQVDLSRRE LSINDEVIKL
KP-37_04233_R_phoP/1-224         KPFHIEEVMA RMQALMRRNS GLASQVISLP PFQVDLSRRE LSINDEVIKL
NCTC-13846_00897_R_phoP/1-224    KPFHIEEVMA RMQALMRRNS GLASQVISLP PFQVDLSRRE LSINDEVIKL
700455-17_04540_R_phoP/1-224     KPFHIEEVMA RMQALMRRNS GLASQVISLP PFQVDLSRRE LSINDEVIKL
705498-12_01205_R_phoP/1-224     KPFHIEEVMA RMQALMRRNS GLASQVISLP PFQVDLSRRE LSINDEVIKL
705963-16_03913_R_phoP/1-224     KPFHIEEVMA RMQALMRRNS GLASQVISLP PFQVDLSRRE LSINDEVIKL
706090-16_02995_R_phoP/1-224     KPFHIEEVMA RMQALMRRNS GLASQVISLP PFQVDLSRRE LSINDEVIKL
700099-17_01721_R_phoP/1-224     KPFHIEEVMA RMQALMRRNS GLASQVISLP PFQVDLSRRE LSINDEVIKL
```

```
                                 151
703692-17_03869_S_phoP/1-224     TAFEYTIMET LIRNNGKVVS KDSLMLQLYP DAELRESHTI DVLMGRLRKK
703694-17_02948_S_phoP/1-224     TAFEYTIMET LIRNNGKVVS KDSLMLQLYP DAELRESHTI DVLMGRLRKK
705882-17_03639_S_phoP/1-224     TAFEYTIMET LIRNNGKVVS KDSLMLQLYP DAELRESHTI DVLMGRLRKK
705889-17_01550_S_phoP/1-224     TAFEYTIMET LIRNNGKVVS KDSLMLQLYP DAELRESHTI DVLMGRLRKK
722306-16_01056_S_phoP/1-224     TAFEYTIMET LIRNNGKVVS KDSLMLQLYP DAELRESHTI DVLMGRLRKK
14929959_04295_S_phoP/1-224      TAFEYTIMET LIRNNGKVVS KDSLMLQLYP DAELRESHTI DVLMGRLRKK
ATCC-25922_03514_S_phoP/1-224    TAFEYTIMET LIRNNGKVVS KDSLMLQLYP DAELRESHTI DVLMGRLRKK
108670-17_01836_S_phoP/1-224     TAFEYTIMET LIRNNGKVVS KDSLMLQLYP DAELRESHTI DVLMGRLRKK
603351-17_02817_S_phoP/1-224     TAFEYTIMET LIRNNGKVVS KDSLMLQLYP DAELRESHTI DVLMGRLRKK
703629-17_02558_S_phoP/1-224     TAFEYTIMET LIRNNGKVVS KDSLMLQLYP DAELRESHTI DVLMGRLRKK
705801-17_04901_S_phoP/1-224     TAFEYTIMET LIRNNGKVVS KDSLMLQLYP DAELRESHTI DVLMGRLRKK
108627-17_04205_S_phoP/1-224     TAFEYTIMET LIRNNGKVVS KDSLMLQLYP DAELRESHTI DVLMGRLRKK
707671-17_00857_R_phoP/1-224     TAFEYTIMET LIRNNGKVVS KDSLMLQLYP DAELRESHTI DVLMGRLRKK
709006-16_01085_R_phoP/1-224     TAFEYTIMET LIRNNGKVVS KDSLMLQLYP DAELRESHTI DVLMGRLRKK
719645-16_02745_R_phoP/1-224     TAFEYTIMET LIRNNGKVVS KDSLMLQLYP DAELRESHTI DVLMGRLRKK
721296-16_01817_R_phoP/1-224     TAFEYTIMET LIRNNGKVVS KDSLMLQLYP DAELRESHTI DVLMGRLRKK
KP-37_04233_R_phoP/1-224         TAFEYTIMET LIRNNGKVVS KDSLMLQLYP DAELRESHTI DVLMGRLRKK
NCTC-13846_00897_R_phoP/1-224    TAFEYTIMET LIRNNGKVVS KDSLMLQLYP DAELRESHTI DVLMGRLRKK
700455-17_04540_R_phoP/1-224     TAFEYTIMET LIRNNGKVVS KDSLMLQLYP DAELRESHTI DVLMGRLRKK
705498-12_01205_R_phoP/1-224     TAFEYTIMET LIRNNGKVVS KDSLMLQLYP DAELRESHTI DVLMGRLRKK
705963-16_03913_R_phoP/1-224     TAFEYTIMET LIRNNGKVVS KDSLMLQLYP DAELRESHTI DVLMGRLRKK
706090-16_02995_R_phoP/1-224     TAFEYTIMET LIRNNGKVVS KDSLMLQLYP DAELRESHTI DVLMGRLRKK
700099-17_01721_R_phoP/1-224     TAFEYTIMET LIRNNGKVVS KDSLMLQLYP DAELRESHTI DVLMGRLRKK

                                 201
703692-17_03869_S_phoP/1-224     IQAQYPQEVI TTVRGQGYLF ELR*
703694-17_02948_S_phoP/1-224     IQAQYPQEVI TTVRGQGYLF ELR*
705882-17_03639_S_phoP/1-224     IQAQYPQEVI TTVRGQGYLF ELR*
705889-17_01550_S_phoP/1-224     IQAQYPQEVI TTVRGQGYLF ELR*
722306-16_01056_S_phoP/1-224     IQAQYPQEVI TTVRGQGYLF ELR*
14929959_04295_S_phoP/1-224      IQAQYPQEVI TTVRGQGYLF ELR*
ATCC-25922_03514_S_phoP/1-224    IQAQYPQEVI TTVRGQGYLF ELR*
108670-17_01836_S_phoP/1-224     IQAQYPQEVI TTVRGQGYLF ELR*
603351-17_02817_S_phoP/1-224     IQAQYPQEVI TTVRGQGYLF ELR*
703629-17_02558_S_phoP/1-224     IQAQYPQEVI TTVRGQGYLF ELR*
705801-17_04901_S_phoP/1-224     IQAQYPQEVI TTVRGQGYLF ELR*
108627-17_04205_S_phoP/1-224     IQAQYPQEVI TTVRGQGYLF ELR*
707671-17_00857_R_phoP/1-224     IQAQYPQEVI TTVRGQGYLF ELR*
709006-16_01085_R_phoP/1-224     IQAQYPQEVI TTVRGQGYLF ELR*
719645-16_02745_R_phoP/1-224     IQAQYPQEVI TTVRGQGYLF ELR*
721296-16_01817_R_phoP/1-224     IQAQYPQEVI TTVRGQGYLF ELR*
KP-37_04233_R_phoP/1-224         IQAQYPQEVI TTVRGQGYLF ELR*
NCTC-13846_00897_R_phoP/1-224    IQAQYPQEVI TTVRGQGYLF ELR*
700455-17_04540_R_phoP/1-224     IQAQYPQEVI TTVRGQGYLF ELR*
705498-12_01205_R_phoP/1-224     IQAQYPQEVI TTVRGQGYLF ELR*
705963-16_03913_R_phoP/1-224     IQAQYPQEVI TTVRGQGYLF ELR*
706090-16_02995_R_phoP/1-224     IQAQYPQEVI TTVRGQGYLF ELR*
700099-17_01721_R_phoP/1-224     IQAQYPQEVI TTVRGQGYLF ELR*
```
