# Supplementary material for: Colistin resistance in Gram-negative bacteria analysed by five phenotypic assays and inference of the underlying genomic mechanisms
Source: BMC Microbiol. 2021 Nov 20;21:321. doi: 10.1186/s12866-021-02388-8 (PMC8605564; doi:10.1186/s12866-021-02388-8)
Supplement: Supplementary file 5 — Additional file 5. Acinetobacter bereziniae protein sequence alignments.pdf showing the protein sequences alignments of all colistin resistance-related proteins analyzed in this study. [file 12866_2021_2388_MOESM5_ESM.pdf]

```
Alignment: D:\Documentos BASILEA\AMR LAB\Colistin resistance project\Genes\Acinetobacter spp\ba
Seaview [blocks=10 fontsize=10 A4] on Wed Jun 03 20:50:44 2020

                                  1
503361-5-15_03109_S_pmrA/1-231   MTKILMIEDD FMIAESSKTL LKYQGFDVEW VNNGLDGLKL ISEKQFDLVL
503777-15_03313_S_pmrA/1-231     MTKILMIEDD FMIAESSKTL LKYQGFDVEW VNNGLDGLKL ISEKQFDLVL
503137-15_04035_S_pmrA/1-231     MTKILMIEDD FMIAESSKTL LKYQGFDVEW VNNGLDGLKL ISEKQFDLVL
502814-15_03711_R_pmrA/1-231     MTKILMIEDD FMIAESSKTL LKYQGFDVEW VNNGLDGLKL ISEKQFDLVL

                                 51
503361-5-15_03109_S_pmrA/1-231   LDLGLPMMDG MQVLKQIRQR SGLPVLIISA RDQLQNRVDG LNQGADDYLI
503777-15_03313_S_pmrA/1-231     LDLGLPMMDG MQVLKQIRQR SGLPVLIISA RDQLQNRVDG LNQGADDYLI
503137-15_04035_S_pmrA/1-231     LDLGLPMMDG MQVLKQIRQR SGLPVLIISA RDQLQNRVDG LNQGADDYLI
502814-15_03711_R_pmrA/1-231     LDLGLPMMDG MQVLKQIRQR SGLPVLIISA RDQLQNRVDG LNQGADDYLI

                                101
503361-5-15_03109_S_pmrA/1-231   KPYEFDELVA RIHALLRRSG DATKEVMGQS SNLLKNGEIV LDVEQHIATL
503777-15_03313_S_pmrA/1-231     KPYEFDELVA RIHALLRRSG DATKEVMGQS SNLLKNGEIV LDVEQHIATL
503137-15_04035_S_pmrA/1-231     KPYEFDELVA RIHALLRRSG DATKEVMGQS SNLLKNGEIV LDVEQHIATL
502814-15_03711_R_pmrA/1-231     KPYEFDELVA RIHALLRRSG DATKEVMGQS SNLLKNGEIV LDVEQHIATL

                                151
503361-5-15_03109_S_pmrA/1-231   KGEPVELSNR EWAILIPLMT HPNKIFSKAN LEDKLYAFDS EINSNTIEVY
503777-15_03313_S_pmrA/1-231     KGEPVELSNR EWAILIPLMT HPNKIFSKAN LEDKLYAFDS EINSNTIEVY
503137-15_04035_S_pmrA/1-231     KGEPVELSNR EWAILIPLMT HPNKIFSKAN LEDKLYAFDS EINSNTIEVY
502814-15_03711_R_pmrA/1-231     KGEPVELSNR EWAILIPLMT HPNKIFSKAN LEDKLYAFDS EINSNTIEVY

                                201
503361-5-15_03109_S_pmrA/1-231   VHHIRSKLGK DIIRTIRGLG YRLGQAHKAE *
503777-15_03313_S_pmrA/1-231     VHHIRSKLGK DIIRTIRGLG YRLGQAHKAE *
503137-15_04035_S_pmrA/1-231     VHHIRSKLGK DIIRTIRGLG YRLGQAHKAE *
502814-15_03711_R_pmrA/1-231     VHHIRSKLGK DIIRTIRGLG YRLGQAHKAE *
```

```
Alignment: D:\Documentos BASILEA\AMR LAB\Colistin resistance project\Genes\Acinetobacter spp\ba
Seaview [blocks=10 fontsize=10 A4] on Wed Jun 03 20:53:43 2020

                                1
503137-15_04036_S_pmrB/1-446    MGTAFSLRKR LIRYTSIFSI LLGCVLIFSA YKISLEEINE ILDAQMVYLA
503361-5-15_03108_S_pmrB/1-446  MGTAFSLRKR LIRYTSIFSI LLGCVLIFSA YKISLEEINE ILDAQMVYLA
503777-15_03314_S_pmrB/1-401    ---------- ---------- ---------- ---------- -----MVYLA
502814-15_03710_R_pmrB/1-446    MGTAFSLRKR LIRYTSIFSI LLGCVLIFSA YKISLEEINE ILDAQMVYLA

                               51
503137-15_04036_S_pmrB/1-446    ERVELNPRPI QSHFDEHKRY HEEDLFIDVW SYANPQQVPN TPHHVIIEPK
503361-5-15_03108_S_pmrB/1-446  ERVELNPRPI QSHFDEHKRY HEEDLFIDVW SYANPQQVPN TPHHVIIEPK
503777-15_03314_S_pmrB/1-401    ERVELNPRPI QSHFDEHKRY HEEDLFIDVW SYANPQQVPN TPHHVIIEPK
502814-15_03710_R_pmrB/1-446    ERVELNPRPI QSHFDEHKRY HEEDLFIDVW SYANPQQVPN TPHHVIIEPK

                              101
503137-15_04036_S_pmrB/1-446    QKAGFYSQET ANGTWITYIL PTANFQIQIS QQEKVREHLA LELAGSMFLP
503361-5-15_03108_S_pmrB/1-446  QKAGFYSQET ANGTWITYIL PTANFQIQIS QQEKVREHLA LELAGSMFLP
503777-15_03314_S_pmrB/1-401    QKAGFYSQET ANGTWITYIL PTANFQIQIS QQEKVREHLA LELAGSMFLP
502814-15_03710_R_pmrB/1-446    QKAGFYSQET ANGTWITYIL PTANFQIQIS QQEKVREHLA LELAGSMFLP

                              151
503137-15_04036_S_pmrB/1-446    YLLIIPFALL GLVYIIRRSL KPLEDFKSEL AKRDSNSLVA IQNAHYPEEL
503361-5-15_03108_S_pmrB/1-446  YLLIIPFALL GLVYIIRRSL KPLEDFKSEL AKRDSNSLVA IQNAHYPEEL
503777-15_03314_S_pmrB/1-401    YLLIIPFALL GLVYIIRRSL KPLEDFKSEL AKRDSNSLVA IQNAHYPEEL
502814-15_03710_R_pmrB/1-446    YLLIIPFALL GLVYIIRRSL KPLEDFKSEL AKRDSNSLVA IQNAHYPEEL

                              201
503137-15_04036_S_pmrB/1-446    LPTIQEMNHL FERISEAQQE QRQFVADAAH ELRTPITALN LQTKILMSQF
503361-5-15_03108_S_pmrB/1-446  LPTIQEMNHL FERISEAQQE QRQFVADAAH ELRTPITALN LQTKILMSQF
503777-15_03314_S_pmrB/1-401    LPTIQEMNHL FERISEAQQE QRQFVADAAH ELRTPITALN LQTKILMSQF
502814-15_03710_R_pmrB/1-446    LPTIQEMNHL FERISEAQQE QRQFVADAAH ELRTPITALN LRTKILMSQF

                              251
503137-15_04036_S_pmrB/1-446    PEEENLKNLS KGLARMQHLV TQLLALAKQD ASLNHEVQVT QFKLNDVALN
503361-5-15_03108_S_pmrB/1-446  PEEENLKNLS KGLARMQHLV TQLLALAKQD ASLNHEVQVT QFKLNDVALN
503777-15_03314_S_pmrB/1-401    PEEENLKNLS KGLARMQHLV TQLLALAKQD ASLNHEVQVT QFKLNDVALN
502814-15_03710_R_pmrB/1-446    PEEENLKNLS KGLARMQHLV TQLLALAKQD ASLNHEVQVT QFKLNDVALN

                              301
503137-15_04036_S_pmrB/1-446    CVEQLMNLAM EKDIDLGFIR NETVVLKSVE HSLHSIIFNL IDNAIKYTPV
503361-5-15_03108_S_pmrB/1-446  CVEQLMNLAM EKDIDLGFIR NETVVLKSVE HSLHSIIFNL IDNAIKYTPV
503777-15_03314_S_pmrB/1-401    CVEQLMNLAM EKDIDLGFIR NETVVLKSVE HSLHSIIFNL IDNAIKYTPV
502814-15_03710_R_pmrB/1-446    CVEQLMNLAM EKDIDLGFIR NETVVLKSVE HSLHSIIFNL IDNAIKYTPV

                              351
503137-15_04036_S_pmrB/1-446    SGMINISVFA DIHDFAYVLI EDSGPGIDTE MYDKVLKRFY RVHHHLEVGS
503361-5-15_03108_S_pmrB/1-446  SGMINISVFA DIHDFAYVLI EDSGPGIDTE MYDKVLKRFY RVHHHLEVGS
503777-15_03314_S_pmrB/1-401    SGMINISVFA DIHDFAYVLI EDSGPGIDTE MYDKVLKRFY RVHHHLEVGS
502814-15_03710_R_pmrB/1-446    SGMINISVFA DIHDFAYVLI EDSGPGIDTE MYDKVLKRFY RVHHHLEVGS

                              401
503137-15_04036_S_pmrB/1-446    GLGLSIVDKA IQHLGGELLL SRSEELGGLS VLVKVPVQLN IKPIE*
503361-5-15_03108_S_pmrB/1-446  GLGLSIVDKA IQHLGGELLL SRSEELGGLS VLVKVPVQLN IKPIE*
503777-15_03314_S_pmrB/1-401    GLGLSIVDKA IQHLGGELLL SRSEELGGLS VLVKVPVQLN IKPIE*
502814-15_03710_R_pmrB/1-446    GLGLSIVDKA IQHLGGELLL SRSEELGGLS VLVKVPVQLN IKPIE*
```

Alignment: D:\Documentos BASILEA\AMR LAB\Colistin resistance project\Genes\Acinetobacter spp\ph
Seaview [blocks=10 fontsize=10 A4] on Wed Jun 03 21:11:00 2020

```
                                1
503137-15_02638_S_phoP/1-128    MDDKFQNLKV MVIDDSKTIR RTAETLLQRE GCEVVTAVDG FEALSKIAEA
503361-5-15_02406_S_phoP/1-128  MDDKFQNLKV MVIDDSKTIR RTAETLLQRE GCEVVTAVDG FEALSKIAEA
503777-15_01887_S_phoP/1-128    MDDKFQNLKV MVIDDSKTIR RTAETLLQRE GCEVVTAVDG FEALSKIAEA
502814-15_02383_R_phoP/1-128    MDDKFQNLKV MVIDDSKTIR RTAETLLQRE GCEVVTAVDG FEALSKIAEA

                               51
503137-15_02638_S_phoP/1-128    NPDIVFVDIM MPRLDGYQTC ALIKNSQNYQ NIPVIMLSSK DGLFDQAKGR
503361-5-15_02406_S_phoP/1-128  NPDIVFVDIM MPRLDGYQTC ALIKNSQNYQ NIPVIMLSSK DGLFDQAKGR
503777-15_01887_S_phoP/1-128    NPDIVFVDIM MPRLDGYQTC ALIKNSQNYQ NIPVIMLSSK DGLFDQAKGR
502814-15_02383_R_phoP/1-128    NPDIVFVDIM MPRLDGYQTC ALIKNSQNYQ NIPVIMLSSK DGLFDQAKGR

                              101
503137-15_02638_S_phoP/1-128    VVGSDEYLTK PFSKDELLNA IRNHVSA*
503361-5-15_02406_S_phoP/1-128  VVGSDEYLTK PFSKDELLNA IRNHVSA*
503777-15_01887_S_phoP/1-128    VVGSDEYLTK PFSKDELLNA IRNHVSA*
502814-15_02383_R_phoP/1-128    VVGSDEYLTK PFSKDELLNA IRNHVSA*
```

Alignment: D:\Documentos BASILEA\AMR LAB\Colistin resistance project\Genes\Acinetobacter spp\Ip
Seaview [blocks=10 fontsize=10 A4] on Wed Jun 03 20:59:29 2020

```
                                 1
503777-15_00489_S_lpxA/1-263     MSSNDLIHPT AIIDASAVIA PDVQIGPYCI IGPQVTIGAG TKLHSHVVIG
503361-5-15_01141_S_lpxA/1-263   MSSNDLIHPT AIIDASAVIA PDVQIGPYCI IGPQVTIGAG TKLHSHVVIG
503137-15_02996_S_lpxA/1-263     MSSNDLIHPT AIIDASAVIA PDVQIGPYCI IGPQVTIGAG TKLHSHVVIG
502814-15_02133_R_lpxA/1-263     MSSNDLIHPT AIIDASAVIA PDVQIGPYCI IGPQVTIGAG TKLHSHVVIG

                                51
503777-15_00489_S_lpxA/1-263     GFTRIGKNNE IFQFASVGEV CQDLKYQGEE TWLEIGDNNK IREHCSLHRG
503361-5-15_01141_S_lpxA/1-263   GFTRIGKNNE IFQFASVGEV CQDLKYQGEE TWLEIGDNNK IREHCSLHRG
503137-15_02996_S_lpxA/1-263     GFTRIGKNNE IFQFASVGEV CQDLKYQGEE TWLEIGDNNK IREHCSLHRG
502814-15_02133_R_lpxA/1-263     GFTRIGKNNE IFQFASVGEV CQDLKYQGEE TWLEIGDNNK IREHCSLHRG

                               101
503777-15_00489_S_lpxA/1-263     TVQDHGLTKV GSNNLLMVNT HIAHDCQIGN NNIFANNVGI AGHVHIGDFV
503361-5-15_01141_S_lpxA/1-263   TVQDHGLTKV GSNNLLMVNT HIAHDCQIGN NNIFANNVGI AGHVHIGDFV
503137-15_02996_S_lpxA/1-263     TVQDHGLTKV GSNNLLMVNT HIAHDCQIGN NNIFANNVGI AGHVHIGDFV
502814-15_02133_R_lpxA/1-263     TVQDHGLTKV GSNNLLMVNT HIAHDCQIGN NNIFANNVGI AGHVHIGDFV

                               151
503777-15_00489_S_lpxA/1-263     IVGGNSGIHQ FCKIDSYSMI GGASLILKDV PAYVMVSGNP AHAYGMNVEG
503361-5-15_01141_S_lpxA/1-263   IVGGNSGIHQ FCKIDSYSMI GGASLILKDV PAYVMVSGNP AHAYGMNVEG
503137-15_02996_S_lpxA/1-263     IVGGNSGIHQ FCKIDSYSMI GGASLILKDV PAYVMVSGNP AHAYGMNVEG
502814-15_02133_R_lpxA/1-263     IVGGNSGIHQ FCKIDSYSMI GGASLILKDV PAYVMVSGNP AHAYGMNVEG

                               201
503777-15_00489_S_lpxA/1-263     MRRKGWSKNV IQGLRESFKL IYKENLTTEQ AIERIRQEIL PEVEEAQLLI
503361-5-15_01141_S_lpxA/1-263   MRRKGWSKNV IQGLRESFKL IYKENLTTEQ AIERIRQEIL PEVEEAQLLI
503137-15_02996_S_lpxA/1-263     MRRKGWSKNV IQGLRESFKL IYKENLTTEQ AIERIRQEIL PEVEEAQLLI
502814-15_02133_R_lpxA/1-263     MRRKGWSKNV IQGLRESFKL IYKENLTTEQ AIERIRQEIL PEVEEAQLLI

                               251
503777-15_00489_S_lpxA/1-263     DSVLESKRGI VR*
503361-5-15_01141_S_lpxA/1-263   DSVLESKRGI VR*
503137-15_02996_S_lpxA/1-263     DSVLESKRGI VR*
502814-15_02133_R_lpxA/1-263     DSVLESKRGI VR*
```

Alignment: D:\Documentos BASILEA\AMR LAB\Colistin resistance project\Genes\Acinetobacter spp\Ip
Seaview [blocks=10 fontsize=10 A4] on Wed Jun 03 21:02:18 2020

```
                               1
503361-5-15_00773_S_lpxC/1-329  VRFITFFVFY QWLNVLTYSG FENEDRISML KQRTLKRVVK ASGIGLHSGQ
503137-15_01180_S_lpxC/1-329    VRFITFFVFY QWLNVLTYSG FENEDRISML KQRTLKRVVK ASGIGLHSGQ
503777-15_00192_S_lpxC/1-329    VRFITFFVFY QWLNVLTYSG FENEDRISML KQRTLKRVVK ASGIGLHSGQ
502814-15_00913_R_lpxC/1-329    VRFITFFVFY QWLNVLTYSG FENEDRISML KQRTLKRVVK ASGIGLHSGQ

                              51
503361-5-15_00773_S_lpxC/1-329  KVMINFLPHV ADGGIVFRRI DLNPPVDIPA DAMLIQEAFM CSNLVSKETK
503137-15_01180_S_lpxC/1-329    KVMINFLPHV ADGGIVFRRI DLNPPVDIPA DAMLIQEAFM CSNLVSKETK
503777-15_00192_S_lpxC/1-329    KVMINFLPHV ADGGIVFRRI DLNPPVDIPA DAMLIQEAFM CSNLVSKETK
502814-15_00913_R_lpxC/1-329    KVMINFLPHV ADGGIVFRRI DLNPPVDIPA DAMLIQEAFM CSNLVSKETK

                             101
503361-5-15_00773_S_lpxC/1-329  VGTIEHVTSA IAGLGIDNLI IEVSASEIPI MDGSAGPFIY LLMQGELVEQ
503137-15_01180_S_lpxC/1-329    VGTIEHVTSA IAGLGIDNLI IEVSASEIPI MDGSAGPFIY LLMQGELVEQ
503777-15_00192_S_lpxC/1-329    VGTIEHVTSA IAGLGIDNLI IEVSASEIPI MDGSAGPFIY LLMQGELVEQ
502814-15_00913_R_lpxC/1-329    VGTIEHVTSA IAGLGIDNLI IEVSASEIPI MDGSAGPFIY LLMQGELVEQ

                             151
503361-5-15_00773_S_lpxC/1-329  DAPKKFIRIL KPVEALIDDK RAIFSPHDGF QINFTIDFDH PAFAKEYQSA
503137-15_01180_S_lpxC/1-329    DAPKKFIRIL KPVEALIDDK RAIFSPHDGF QINFTIDFDH PAFAKEYQSA
503777-15_00192_S_lpxC/1-329    DAPKKFIRIL KPVEALIDDK RAIFSPHDGF QINFTIDFDH PAFAKEYQSA
502814-15_00913_R_lpxC/1-329    DAPKKFIRIL KPVEALIDDK RAIFSPHDGF QINFTIDFDH PAFAKEYQSA

                             201
503361-5-15_00773_S_lpxC/1-329  TIDFSTETFV YEVSGARTFG FMKDLDYLKA NNLALGASLD NAVGLDDTGV
503137-15_01180_S_lpxC/1-329    TIDFSTETFV YEVSGARTFG FMKDLDYLKA NNLALGASLD NAVGLDDTGV
503777-15_00192_S_lpxC/1-329    TIDFSTETFV YEVSGARTFG FMKDLDYLKA NNLALGASLD NAVGLDDTGV
502814-15_00913_R_lpxC/1-329    TIDFSTETFV YEVSGARTFG FMKDLDYLKA NNLALGASLD NAVGLDDTGV

                             251
503361-5-15_00773_S_lpxC/1-329  VNEEGLRFAD EFVRHKILDA VGDLYLLGHQ IIAKFDGYKS GHALNNQLLR
503137-15_01180_S_lpxC/1-329    VNEEGLRFAD EFVRHKILDA VGDLYLLGHQ IIAKFDGYKS GHALNNQLLR
503777-15_00192_S_lpxC/1-329    VNEEGLRFAD EFVRHKILDA VGDLYLLGHQ IIAKFDGYKS GHALNNQLLR
502814-15_00913_R_lpxC/1-329    VNEEGLRFAD EFVRHKILDA VGDLYLLGHQ IIAKFDGYKS GHALNNQLLR

                             301
503361-5-15_00773_S_lpxC/1-329  NVKSDPSSYE IVTFDDEKLC PIHFVNVT*
503137-15_01180_S_lpxC/1-329    NVKSDPSSYE IVTFDDEKLC PIHFVNVT*
503777-15_00192_S_lpxC/1-329    NVKSDPSSYE IVTFDDEKLC PIHFVNVT*
502814-15_00913_R_lpxC/1-329    NVKSDPSSYE IVTFDDEKLC PIHFVNVT*
```

Alignment: D:\Documentos BASILEA\AMR LAB\Colistin resistance project\Genes\Acinetobacter spp\Ip
Seaview [blocks=10 fontsize=10 A4] on Wed Jun 03 21:04:27 2020

```
                              1
503137-15_02998_S_lpxD/1-357     MRSNQFHLDE LAQLVQGELI GQPKLLLSGL ASLEQAQSHH IAFVNGDKYL
503361-5-15_01139_S_lpxD/1-357   MRSNQFHLDE LAQLVQGELI GQPKLLLSGL ASLEQAQSHH IAFVNGDKYL
503777-15_00487_S_lpxD/1-357     MRSNQFHLDE LAQLVQGELI GQPKLLLSGL ASLEQAQSHH IAFVNGDKYL
502814-15_02131_R_lpxD/1-357     MRSNQFHLDE LAQLVQGELI GQPKLLLSGL ASLEQAQSHH IAFVNGDKYL

                             51
503137-15_02998_S_lpxD/1-357     AEAQNSKAGA LIVTAALKEQ LSNHQNFIVV ANPYLAFAML THQFEIKVTK
503361-5-15_01139_S_lpxD/1-357   AEAQNSKAGA LIVTAALKEQ LSNHQNFIVV ANPYLAFAML THQFEIKVTK
503777-15_00487_S_lpxD/1-357     AEAQNSKAGA LIVTAALKEQ LSNHQNFIVV ANPYLAFAML THQFEIKVTK
502814-15_02131_R_lpxD/1-357     AEAQNSKAGA LIVTAALKEQ LSNHQNFIVV ANPYLAFAML THQFEIKVTK

                            101
503137-15_02998_S_lpxD/1-357     RGIESTAQIS PSAIIADDAY IGHYVVIGED CVIGSNTIVQ SNTRIDDGVE
503361-5-15_01139_S_lpxD/1-357   RGIESTAQIS PSAIIADDAY IGHYVVIGED CVIGSNTIVQ SNTRIDDGVE
503777-15_00487_S_lpxD/1-357     RGIESTAQIS PSAIIADDAY IGHYVVIGED CVIGSNTIVQ SNTRIDDGVE
502814-15_02131_R_lpxD/1-357     RGIESTAQIS PSAIIADDAY IGHYVVIGED CVIGSNTIVQ SNTRIDDGVE

                            151
503137-15_02998_S_lpxD/1-357     IGKDCFVDSQ VTLTGQTKIA DRVRIHANTV IGGEGFGFAP YQGKWNRIAQ
503361-5-15_01139_S_lpxD/1-357   IGKDCFVDSQ VTLTGQTKIA DRVRIHANTV IGGEGFGFAP YQGKWNRIAQ
503777-15_00487_S_lpxD/1-357     IGKDCFVDSQ VTLTGQTKIA DRVRIHANTV IGGEGFGFAP YQGKWNRIAQ
502814-15_02131_R_lpxD/1-357     IGKDCFVDSQ VTLTGQTKIA DRVRIHANTV IGGEGFGFAP YQGKWNRIAQ

                            201
503137-15_02998_S_lpxD/1-357     LGSVRIGNDV RIGSNCSVDR GALDDTIIHD GVIIDNLVQI AHNVQIGENT
503361-5-15_01139_S_lpxD/1-357   LGSVRIGNDV RIGSNCSVDR GALDDTIIHD GVIIDNLVQI AHNVQIGENT
503777-15_00487_S_lpxD/1-357     LGSVRIGNDV RIGSNCSVDR GALDDTIIHD GVIIDNLVQI AHNVQIGENT
502814-15_02131_R_lpxD/1-357     LGSVRIGNDV RIGSNCSVDR GALDDTIIHD GVIIDNLVQI AHNVQIGENT

                            251
503137-15_02998_S_lpxD/1-357     AIAAKCGIAG STIIGKNCIF AGAVGVVGHI KIADNVTITG MSMVTKSISE
503361-5-15_01139_S_lpxD/1-357   AIAAKCGIAG STIIGKNCIF AGAVGVVGHI KIADNVTITG MSMVTKSISE
503777-15_00487_S_lpxD/1-357     AIAAKCGIAG STIIGKNCIF AGAVGVVGHI KIADNVTITG MSMVTKSISE
502814-15_02131_R_lpxD/1-357     AIAAKCGIAG STIIGKNCIF AGAVGVVGHI KIADNVTITG MSMVTKSISE

                            301
503137-15_02998_S_lpxD/1-357     AGSYSSGTGQ FETNHWKRTV VRLRQLADVP LTKLVKQIDH MQAQIESIES
503361-5-15_01139_S_lpxD/1-357   AGSYSSGTGQ FETNHWKRTV VRLRQLADVP LTKLVKQIDH MQAQIESIES
503777-15_00487_S_lpxD/1-357     AGSYSSGTGQ FETNHWKRTV VRLRQLADVP LTKLVKQIDH MQAQIESIES
502814-15_02131_R_lpxD/1-357     AGSYSSGTGQ FETNHWKRTV VRLRQLADVP LTKLVKQIDH MQAQIESIES

                            351
503137-15_02998_S_lpxD/1-357     TLKSRK*
503361-5-15_01139_S_lpxD/1-357   TLKSRK*
503777-15_00487_S_lpxD/1-357     TLKSRK*
502814-15_02131_R_lpxD/1-357     TLKSRK*
```
