# Supplementary material for: Colistin resistance in Gram-negative bacteria analysed by five phenotypic assays and inference of the underlying genomic mechanisms
Source: BMC Microbiol. 2021 Nov 20;21:321. doi: 10.1186/s12866-021-02388-8 (PMC8605564; doi:10.1186/s12866-021-02388-8)
Supplement: Supplementary file 6 — Additional file 6. Species distribution as determined by MALDI-TOF MS. A total of 97 isolates were investigated, including 93 collected collected from the Basel University Hospital (Basel, Switzerland), Cantonal Hospital Luzerne (Luzerne, Switzerland) and Laboratory Viollier (Allschwil, Switzerland) [file 12866_2021_2388_MOESM6_ESM.docx]

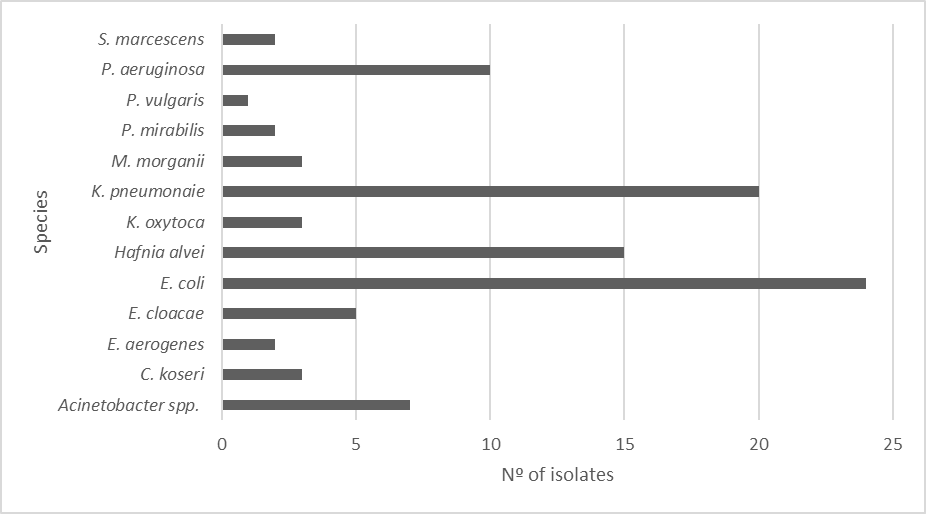


Additional file 6. Species distribution as determined by MALDI-TOF MS*. A total of 97 isolates were investigated, including 93 collected collected from the Basel University Hospital (Basel, Switzerland), Cantonal Hospital Luzerne (Luzerne, Switzerland) and Laboratory Viollier (Allschwil, Switzerland)*
