# Supplementary material for: Colistin resistance in Gram-negative bacteria analysed by five phenotypic assays and inference of the underlying genomic mechanisms
Source: BMC Microbiol. 2021 Nov 20;21:321. doi: 10.1186/s12866-021-02388-8 (PMC8605564; doi:10.1186/s12866-021-02388-8)
Supplement: Supplementary file 7 — Additional file 7. Different phenotypic assays used for colistin susceptibility testing: UMIC, Colistin E-Test MIC strip and Rapid Polymyxin NP Test.docx showing example images of the phenotypic assays using in this study. [file 12866_2021_2388_MOESM7_ESM.docx]

Additional file 7. Different phenotypic assays used for colistin susceptibility testing: UMIC, E-Test and Rapid Polymyxin NP Test.
